# Health burden in type 2 diabetes and prediabetes in The Maastricht Study

**DOI:** 10.1038/s41598-022-11136-5

**Published:** 2022-05-05

**Authors:** Marja G. J. Veugen, Veronica G. Onete, Ronald M. A. Henry, Hans-Peter Brunner-La Rocca, Annemarie Koster, Pieter C. Dagnelie, Nicolaas C. Schaper, Simone J. S. Sep, Carla J. H. van der Kallen, Martin P. J. van Boxtel, Koen D. Reesink, Johannes S. Schouten, Hans H. C. M. Savelberg, Sebastian Köhler, Frans R. Verhey, Joop P. W. van den Bergh, Miranda T. Schram, Coen D. A. Stehouwer

**Affiliations:** 1 Department of Internal Medicine, Maastricht University Medical Centre +, P. Debyelaan 25, P.O. Box 5800, 6202AZ Maastricht, The Netherlands; 2 CARIM School for Cardiovascular Diseases, Maastricht University, Maastricht, The Netherlands; 3 Heart and Vascular Centre, Maastricht University Medical Centre +, Maastricht, The Netherlands; 4 Department of Cardiology, Maastricht University Medical Centre +, Maastricht, the Netherlands; 5 CAPHRI Care and Public Health Research Institute, Maastricht University, Maastricht, The Netherlands; 6 Department of Social Medicine, Maastricht University, Maastricht, The Netherlands; 7 Adelante, Centre of Expertise in Rehabilitation and Audiology, Hoensbroek, The Netherlands; 8 Department of Psychiatry and Neuropsychology and MHeNS School for Mental Health and Neuroscience, Maastricht University, Maastricht, The Netherlands; 9 Department of Biomedical Engineering, Maastricht University Medical Centre +, Maastricht, The Netherlands; 10 Department of Ophthalmology, Maastricht University Medical Centre +, Maastricht, The Netherlands; 11 Canisius-Wilhelmina Hospital, Nijmegen, The Netherlands; 12 Department of Human Movement Sciences, Maastricht University, Maastricht, The Netherlands; 13 Department of Family Medicine, Maastricht University, Maastricht, The Netherlands; 14 Department of Internal Medicine, Subdivision of Rheumatology, Maastricht University Medical Centre+, Maastricht, The Netherlands; 15 Department of Internal Medicine, Subdivision of Endocrinology, VieCuri Medical Center, Venlo, The Netherlands

**Keywords:** Epidemiology, Diabetes, Pre-diabetes

## Abstract

Mortality in type 2 diabetes, is determined not only by classical complications, but also by comorbidities, and is linked to hyperglycaemia and apparent even in prediabetes. We aimed to comprehensively investigate, in a population-based cohort, health burden defined as the presence of comorbidities in addition to classical complications and cardiometabolic risk factors, in not only type 2 diabetes but also prediabetes. Such population-based study has not been performed previously. Extensive phenotyping was performed in 3,410 participants of the population-based Maastricht Study (15.0% prediabetes and 28.6% type 2 diabetes) to assess presence of 17 comorbidities, six classical complications, and ten cardiometabolic risk factors. These were added up into individual and combined sum scores and categorized. Group differences were studied with multinomial regression analyses adjusted for age and sex. Individuals with type 2 diabetes and prediabetes, as compared to normal glucose metabolism (NGM), had greater comorbidities, classical complications, cardiometabolic risk factors and combined sum scores (comorbidities sum score ≥ 3: frequencies (95% CI) 61.5% (57.6;65.4) and 41.2% (36.5;45.9) vs. 25.4% (23.5;27.4), p-trend < 0.001; classical complications ≥ 2 (26.6% (23.1;30.1; *P* < 0.001 vs. NGM) and 10.1% (7.8;12.7; *P* = 0.065 vs NGM) vs. 8.0% (6.9;9.3)); cardiometabolic risk factors ≥ 6 (39.7% (35.9;43.4) and 28.5% (24.5;32.6) vs. 14.0% (12.5;15.6); p-trend < 0.001); combined ≥ 8 (66.6% (62.7;70.5) and 48.4% (43.7;53.1) vs. 26.0%(24.1;28.0), p-trend < 0.001). Type 2 diabetes and prediabetes health burden was comparable to respectively 32 and 14 years of ageing. Our population-based study shows, independently of age and sex, a considerable health burden in both type 2 diabetes and prediabetes, which to a substantial extent can be attributed to comorbidities in addition to classical complications and cardiometabolic risk factors. Our findings emphasize the necessity of comorbidities’ awareness in (pre)diabetes and for determining the exact role of hyperglycaemia in the occurrence of comorbidities.

## Introduction

Recent data show that excess mortality in type 2 diabetes is caused not only by its classical complications (i.e. cardiovascular disease, nephropathy and neuropathy)^1–4^, but also by other, non-classical, comorbidities^5^. This pattern of excess mortality is closely linked to hyperglycaemia, and is apparent even in the prediabetic range^5^. These findings underline the need for detailed study of a broad range of disease outcomes and health determinants, i.e. health burden,in type 2 diabetes and prediabetes as compared to normal glucose metabolism (NGM)^5,6^.

To the best of our knowledge*,* health burden underlying excess mortality in type 2 diabetes and prediabetes, as defined by the presence of comorbidities, classical complications, and cardiometabolic risk factors, has not been comprehensively and quantitatively examined and compared to NGM in a population-based study. Previous population-based studies that have compared disease outcomes and health determinants in individuals with and without type 2 diabetes have focused on multiple classical complications and cardiometabolic risk factors, but not comorbidities^7–9^; have examined only one or a few comorbidities, such as depression and anxiety disorder^10^. Other such previous population-based studies have reported a count measure including comorbidities to quantify health burden in type 2 diabetes^11–15^, but have not comprehensively investigated multiple comorbidities^12,13^, and/or have not considered prediabetes^11–15^ (for a complete overview see the supplementary material).

In the view of the above, we aimed to comprehensively investigate, in a well-characterized population-based cohort, health burden as defined by the presence of a diverse set of non-classical comorbidities in addition to the presence of classical diabetes complications and cardiometabolic risk factors, in not only type 2 diabetes but also prediabetes.

## Methods

### The Maastricht Study: population and design

We used data from The Maastricht Study, an observational prospective population-based cohort study. The rationale and methodology have been described previously^6^. In brief, the study focuses on the etiology, pathophysiology, complications and comorbidities of type 2 diabetes and is characterized by an extensive phenotyping approach^6^. Eligible for participation were all individuals aged between 40 and 75 years living in the southern part of the Netherlands. Participants were recruited through mass media campaigns and from the municipal registries and the regional Diabetes Patient Registry via mailings. Recruitment was stratified according to known type 2 diabetes status, with an oversampling of individuals with type 2 diabetes, for reasons of efficiency, i.e. to increase statistical power to identify any potential contrasts between individuals with and without type 2 diabetes^6^. The present report includes cross-sectional data from the first 3,451 participants, who completed the baseline survey between November 2010 and September 2013. After excluding individuals with type 1 diabetes and other type of diabetes (e.g. pancreatectomy, latent autoimmune diabetes of adults or steroid-induced) the study population consisted of 3,410 individuals. The examinations of each participant were performed within a time window of three months. The study has been approved by the institutional medical ethical committee (NL31329.068.10) and the Minister of Health, Welfare and Sports of the Netherlands (Permit 131,088–105,234-PG) and follows the Declaration of Helsinki. All participants gave written informed consent. The present study was reported as per the STROBE statement for observational cohort studies (and assessed with the STROBE checklist).

### Glucose metabolism status

Glucose metabolism status was assessed and classified according to the World Health Organization 2006 criteria as described previously^6^. Impaired fasting glucose and impaired glucose tolerance were combined into prediabetes.

### Health burden

Health burden was assessed by the presence of 17 (non-classical) comorbidities, six classical complications, and ten cardiometabolic risk factors defined and collected as described in detail in the electronic supplement and summarized below.

### Comorbidities

We aimed to investigate comorbidities in a comprehensive manner based on their association with (pre)diabetes as reported in previous studies^5,16–22^ and the data currently available in our study, which excluded nonhepatic digestive system disorders^5^, periodontal disease^16^, psoriasis^22^, osteopenia and osteoporosis^23^, and hypogonadism^24^.

Dyspnoea as a proxy for pulmonary and cardiac disease was defined as self-reported dyspnoea complaints based on Rose Questionnaire items, or self-reported dyspnoea complaints based on Rose Questionnaire items complemented with self-reported treatment for dyspnoea complaints by a doctor. Limitations in mobility were defined as having mild self-reported difficulties walking 500 m or climbing the stairs, or severe self-reported difficulties walking 500 m or climbing the stairs, as obtained from the Short Form Health Survey (SF-36) questionnaire. Prior skin malignancy was defined as self-reported medical history of skin malignancy which was treated by a doctor. Prior malignancy was defined as self-reported medical history of malignancy which was treated by a doctor. Any thyroid disorder was defined as use of chronic thyroid medication. Prior bone fracture was defined as having had a fracture. Recent acute infection was defined as self-reported symptoms of a lower respiratory, gastrointestinal or urinary tract infection in the previous two months. Polypharmacy was defined as use of five or more different chronic Anatomical Therapeutic Chemical (ATC)-3-medication groups according to the Dutch College of General Practitioners multidisciplinary guideline on polypharmacy in the elderly. Hearing loss was defined as possible mild or moderate to severe hearing loss based on the best ear, as assessed by the HearCheck Navigator (Siemens, Erlangen, Germany), and/or self-reported use of bilateral hearing aids. Cognitive impairment was defined as the need for additional cognitive diagnostics after the first cognitive test battery based on either having an age-, sex- and educational level-adjusted score below 1.5 SD on either the immediate recall, delayed recall, or STROOP III test; having a MMSE score below 24; or when two or more tests were not performed because of known cognitive impairment. Current depression was defined as current major depressive disorder as assessed with the Mini-International Neuropsychiatric Interview (MINI). Anxiety disorder was defined as having a score of ten or greater on the Generalized Anxiety Disorder 7 (GAD-7) questionnaire. Atrial fibrillation was defined as persistent atrial fibrillation or atrial flutter as classified by The Minnesota Code Classification System for electrocardiographic findings code 8–3-1 or 8–3-2. Ocular hypertension was defined as having an intra-ocular pressure higher than 21 mmHg in either the right or the left eye, and/or the use of medication for glaucoma. Anaemia was defined as plasma hemoglobin < 13 g/dL (< 8.1 mmol/L) in men or < 12 g/dL (< 7.5 mmol/L) in women and/or supplemental medication use for anaemia. Non-alcoholic fatty liver disease was defined as an intrahepatic lipid content ≥ 5.56% as quantified on abdominal 3 T-Dixon-MRI^25,26^. Obstructive sleep apnoea was defined as a high risk of obstructive sleep apnoea as determined with an adapted version of the Berlin questionnaire^27^.

Further details on the assessments of comorbidities are described in the supplementary methods.

### Classical complications

Prior coronary heart disease (CHD) was defined as probable coronary heart disease as classified by the Whitehall Criteria by use of The Minnesota Code Classification System for electrocardiographic findings, and/or angina pectoris as assessed by the Rose questionnaire, and/or self-reported prior myocardial infarction, and/or self-reported medical history of coronary percutaneous angioplasty and/or coronary bypass surgery. Prior cerebrovascular disease was defined as ischemic stroke on brain MRI, and/or self-reported medical history of prior stroke (cerebral haemorrhage or infarction), and/or self-reported medical history of carotid percutaneous angioplasty and/or surgery. Prior peripheral artery disease was defined as an ankle-brachial index below 0.9 or above 1.3, and/or intermittent claudication as assessed by the Rose questionnaire, and/or self-reported medical history of leg angioplasty and/or leg surgery; or a self-reported medical history of amputation. Diabetic retinopathy was assessed with fundus photography. Chronic kidney disease was defined as an eGFR below 60 ml/min/1.73 m^2^, (micro)albuminuria, or both; and (or) a self-reported medical history of kidney transplantation or dialysis. Diabetic sensoric neuropathy was defined as neuropathic pain based on the DN4-questionnaire, impaired uni- or bilateral peripheral vibration perception as tested with a Horwell Neurothesiometer at the distal phalanx of the hallux of the right and left foot (Scientific Laboratory Supplies, Nottingham, UK), or both. Impaired peripheral vibration perception was defined as a neurothesiometer score greater than the predicted 97.5 percentile for the individual’s sex and height. Further details on the assessments of classical complications are described in the supplementary methods.

### Cardiometabolic risk factors

Smoking was defined as self-reported former smoker or current smoker. Alcohol use was defined as self-reported low-consumer (≤ 7 glasses per week for women; ≤ 14 glasses per week for men) or high-consumer (> 7 glasses per week for women; > 14 glasses per week for men). Obesity was defined as a BMI of 30 kg/m^2^ or greater according to the International Classification of Diseases 11 (ICD-11). Hypertension was defined as office systolic pressure ≥ 140 mmHg, diastolic pressure ≥ 90 mmHg and/or the use of antihypertensive medication. Non-compliance with physical activity guidelines was defined as less than 2.5 h per week moderate to vigorous physical activity as measured by the mean time in minutes spent in higher intensity physical activity per day (minutes with a step frequency of > 110 steps/min during waking time), by use of the activPAL3 physical activity monitor. Sedentary behaviour was defined as the mean time in minutes spent in a sedentary position during waking time per day above the age- and sex-specific 90th percentile in individuals with NGM, by use of the activPAL3 physical activity monitor (PAL Technologies, Glasgow, UK). Subclinical atherosclerosis was defined as a carotid intima-media thickness above the 90th percentile of the age- and sex-specific normal values according to The Reference Values for Arterial Measurements Collaboration in individuals without prior cardiovascular disease. Aortic stiffness was defined as a carotid-to-femoral pulse wave velocity above the 90th percentile of the age-specific normal values according to The Reference Values for Arterial Stiffness’ Collaboration in individuals without prior cardiovascular disease. In this definition, prior cardiovascular disease was defined as presence of prior coronary heart disease, cerebrovascular disease, and/or peripheral artery disease, and/or dyspnoea. Dyslipidaemia was defined as HDL cholesterol level < 40 mg/dL (< 1.03 mmol/L) in men or < 50 mg/dL (< 1.29 mmol/L) in women, LDL cholesterol level > 100 mg/dL (> 2.5 mmol/L), and/or use of statins, fibrates, or other lipid-modifying medication. Hyperuricaemia was defined as uric acid > 6 mg/dL (0.357 mmol/L) and/or use of urate-lowering therapy. Further details on the assessments of cardiometabolic risk factors are described in the supplementary methods.

### Statistical analyses

All statistical analyses were performed using IBM SPSS Statistics version 23 (IBM Corp, Armonk, NY, USA). A two-sided *P*-value < 0.05 was considered statistically significant.

First, in individuals with prediabetes and type 2 diabetes, all prevalences were directly age- and sex-standardized according to the population structure of the NGM group. For large numbers of events (i.e. age- and sex standardized proportions ≥ 5%) 95% confidence intervals (95% CI) were calculated according to the normal approximation for weighted sums of binomially distributed variables^28^. For small numbers of events (< 5%) the method described by Dobson et al. for the Poisson distribution^29^ was used instead. Similar principles were used for the approximation of 95% CI of proportions in the NGM group.

Age- and sex-adjusted linear trends and differences in the prevalences of comorbidities, classical complications, and cardiometabolic risk factors among groups of glucose metabolism status were tested with (multinomial) logistic regression analyses with NGM or prediabetes as reference category. We chose logistic regression analyses over a Cochran-Mantel–Haenszel test because of the ability to correct for age as a continuous variable (and both approaches give similar results)^30^.

Second, the numbers of comorbidities, classical complications, and cardiometabolic risk factors (as dichotomized variables) present were added up to obtain, per individual, sum scores of comorbidities, classical complications, and cardiometabolic risk factors, as well as a combined health burden sum score. Linear trends and differences in crude sum scores among groups of glucose metabolism status were tested with analyses of variance or independent t-tests as appropriate. Age- and sex-adjusted linear trends and differences in sum scores among groups of glucose metabolism status were tested with linear regression analyses. We used the categorical variable glucose metabolism status (NGM = 0, prediabetes = 1, and type 2 diabetes = 2), or dummy variables for prediabetes and type 2 diabetes with NGM as a reference category, as appropriate. Age-and sex-adjusted mean values (standard error) were calculated with analyses of covariance.

Third, we arranged all sum scores into numeric categories with the cut-offs at the 80th and 50th percentile of the respective sum score in the NGM group (under the assumption that all sum scores were divided equally in the NGM (reference) group), and a separate category for the sum score zero if appropriate (comorbidities 0, 1, 2, ≥ 3; classical complications 0, 1, ≥ 2; cardiometabolic risk factors, 0–4, 4–6, and ≥ 6; and health burden, 0–6, 6–8, and ≥ 8, respectively). These sum score frequencies were age- and sex-standardized as described above. Age- and sex-adjusted linear trends and differences in categories of sum scores among groups of glucose metabolism status were tested with multinomial logistic regression analyses with NGM or prediabetes and the lowest sum score category as reference group.

We performed analyses in the (total) study population (N = 3,410) under the conservative assumption that each classical complication, comorbidity or risk factor was not present unless there was data to meet the criteria of each classical complication, comorbidity or risk factor. Missing data were thus considered as indicating absence of an abnormal result. To test this assumption, we repeated the analyses in the complete case study population for all sum scores (comorbidities, N = 906; classical complications, N = 2,286; cardiometabolic risk factors, N = 2,539; and health burden, N = 653). Finally, we used interaction terms in the regression analyses to examine whether the associations of type 2 diabetes and prediabetes, as compared to NGM, with the sum scores were modified by sex (P_interaction_ < 0.10 was considered statistically significant).

### Ethics approval

This study was performed in line with the principles of the Declaration of Helsinki. The study has been approved by the institutional medical ethical committee (NL31329.068.10) and the Minister of Health, Welfare and Sports of the Netherlands (Permit 1 31 088–1 05 234 PG).

### Consent to participate

Informed consent was obtained from all individual participants included in the study.

### Consent for publication

Patient consent for publication was not required. The participants were invited on yearly events where the most recent findings were discussed, but they had no role in the design of the study.

### Transparency statement

The main guarantor C.S. affirms that the manuscript is an honest, accurate, and transparent account of the study being reported; that no important aspects of the study have been omitted; and that any discrepancies from the study as planned (and, if relevant, registered) have been explained.

## Results

### Characteristics of the study population

Table 1 shows the prevalences of comorbidities, classical complications, and cardiometabolic risk factors according to glucose metabolism status. The study population (N = 3,410; mean (± SD) age, 60 ± 8 years, 1,756 (51%) men) consisted of 1,924 individuals with NGM, 511 with prediabetes, and 975 with type 2 diabetes.

**Table 1:** Age- and sex- adjusted prevalences of comorbidities, classical complications and cardiometabolic risk factors according to glucose metabolism status.

	Normal glucose metabolism (N = 1,924)		Prediabetes (N = 511)		Type 2 diabetes (N = 975)			Numbers available according to glucose metabolism status
**Descriptive variables**							**P-linear**	
Men, n (%)	821 (42.7)		275 (53.8)		660 (67.7)		< 0.001^a,b,c^	1,924/511/975
Age, mean years (SD)	57.9 (8.2)		61.6 (7.6)		62.7 (7.7)		< 0.001^a,b,c^	1,924/511/975
Diabetes duration, median years [IQR]	–		–		7.0 [3.0–12.0]		–	–^d^
**Comorbidities**	**%**	**95% CI**	**%**	**95% CI**	**%**	**95%** **CI**	**P-linear**	
Dyspnoea								1,891/501/932
Dyspnoea complaints	19.3	(17.5–21.1)	27.3	(23.1–31.5)	37.2	(33.2–41.2)	< 0.001^a,b,c^	
Dyspnoea complaints treated by doctor	4.8	(3.9–5.9)	3.7	(2.1–6.2)	9.2	(6.7–11.6)	< 0.001^b,c^	
Limitations in mobility								1,885/499/924
Mild difficulties	12.9	(11.4–14.4)	20.6	(16.7–24.5)	31.2	(27.4–35.1)	< 0.001^a,b,c^	
Severe difficulties	0.9	(0.5–1.4)	2.1	(1.0–3.9)	7.4	(5.4–9.4)	< 0.001^a,b,c^	
Prior skin malignancy	5.4	(4.5–6.5)	5.4	(3.5–7.3)	4.9	(3.3–6.8)	0.062^b^	1,894/503/937
Prior malignancy	4.7	(3.8–5.8)	6.9	(4.7–9.1)	7.1	(5.1–9.0)	0.042^a^	1,896/502/937
Any thyroid disorder	3.0	(2.3–3.9)	4.1	(2.3–6.6)	6.1	(3.9–8.3)	0.001^b^	1,922/510/974
Prior bone fracture	37.8	(35.6–40.1)	42.0	(37.1–46.8)	40.8	(36.5–45.1)	0.803	1,820/474/838
Recent acute infection	23.9	(21.9–26.0)	22.6	(18.2–27.0)	30.0	(26.0–34.0)	0.003^b,c^	1,708/445/864
Polypharmacy	7.2	(6.1–8.4)	11.7	(8.9–14.5)	46.8	(42.8–50.8)	< 0.001^a,b,c^	1,922/510/974
Hearing loss	9.0	(7.8–10.4)	12.8	(10.1–15.6)	12.9	(10.8–15.1)	0.001^a,b^	1,892/505/959
Cognitive impairment	12.8	(11.4–14.4)	14.2	(10.8–17.7)	22.0	(18.6–25.4)	< 0.001^b,c^	1,867/498/926
Current depression	2.7	(2.0–3.6)	2.9	(1.4–5.2)	8.4	(5.8–10.9)	< 0.001^b,c^	1,854/495/918
Anxiety disorder	4.3	(3.4–5.4)	4.6	(2.5–7.7)	8.0	(5.5–10.6)	0.003^b,c^	1,743/457/825
Atrial fibrillation	0.6	(0.3–1.0)	0.5	(0.1–1.4)	1.1	(0.5–1.8)	0.115	1,884/496/949
Ocular hypertension	4.3	(3.3–5.6)	6.9	(4.0–9.7)	7.2	(4.9–9.5)	0.011^b^	1,412/363/693
Anaemia	4.4	(3.5–5.5)	3.6	(1.9–6.0)	11.2	(8.5–13.9)	< 0.001^b,c^	1,864/500/920
Non-alcoholic fatty liver disease	16.5	(14.7–18.6)	37.2	(31.5–42.8)	51.6	(46.2–57.1)	< 0.001^a,b,c^	1,353/342/507
Obstructive sleep apnoea	18.9	(16.9–20.9)	38.8	(33.3–44.2)	51.7	(46.3–57.1)	< 0.001^a,b,c^	1,450/357/592
**Classical complications**	**%**	**95% CI**	**%**	**95% CI**	**%**	**95% CI**	**P-linear**	
Prior coronary heart disease	8.9	(7.7–10.3)	12.3	(9.2–15.5)	23.4	(20.0–26.8)	< 0.001^a,b,c^	1,788/470/902
Prior cerebrovascular disease	2.8	(2.1–3.6)	4.1	(2.4–6.4)	5.0	(3.2–7.1)	0.025^b^	1,916/507/957
Prior peripheral artery disease								1,922 /510/972
Ankle-brachial index < 0.9 or > 1.3 or intermittent claudication complaints or medical history of leg angioplasty/ surgery	19.0	(17.3–20.8)	13.4	(10.5–16.3)	20.7	(17.5–23.9)	0.771^a,c^	
Medical history of amputation	0.4	(0.2–0.8)	0.4	(0.1–1.3)	1.3	(0.6–2.3)	0.007^b^	
Diabetic retinopathy	0.1	(0.0–0.4)	0.2	(0.0–0.8)	4.1	(2.6–6.0)	< 0.001^b,c^	1,531/416/876
Chronic kidney disease								1,885/504/951
eGFR < 60 ml/min/1.73 m^2^ or albuminuria	4.8	(3.8–5.9)	6.2	(4.3–8.2)	17.2	(14.4–20.1)	< 0.001^b,c^	
Both, or a history of kidney transplantation or haemodialysis	0.7	(0.4–1.3)	0.9	(0.3–2.1)	2.4	(1.3–3.7)	< 0.001^b,c^	
Diabetic sensory neuropathy								1,655/428/831
Neuropathic pain or disturbed bilateral peripheral vibration perception	12.1	(10.6–13.7)	17.7	(13.9–21.6)	27.4	(23.5–31.2)	< 0.001^a,b,c^	
Both	0.8	(0.5–1.4)	0.5	(0.1–1.4)	4.7	(3.2–6.6)	< 0.001^b,c^	
**Cardiometabolic risk factors**	**%**	**95% CI**	**%**	**95% CI**	**%**	**95% CI**	**P-linear**	
Smoking								1,903/504/942
Never	39.0	(36.9–41.2)	32.2	(27.7–36.7)	32.7	(28.8–36.6)		
Former	48.1	(45.8–50.3)	53.3	(48.5–58.0)	49.2	(45.1–53.2)	0.005^a,b^	
Current	12.9	(11.4–14.5)	14.6	(11.0–18.1)	18.2	(14.9–21.5)	< 0.001^b^	
Alcohol use								1,898/503/942
None	13.7	(12.2–15.2)	18.4	(14.5–22.3)	39.0	(35.0–42.9)		
Low	58.5	(56.3–60.7)	52.8	(48.0–57.6)	45.4	(41.5–49.4)	< 0.001^a,b,c^	
High	27.8	(25.8–29.8)	28.8	(24.5–33.0)	15.6	(12.9–18.3)	< 0.001^b,c^	
Obesity	11.0	(9.7–12.5)	25.7	(21.4–29.9)	46.6	(42.6–50.6)	0.004^a,b,c^	1,923/511/973
Hypertension	40.7	(38.5–42.9)	57.3	(52.6–62.0)	76.4	(72.8–80.0)	< 0.001^a,b,c^	1,921/509/974
Non-compliance with physical activity guidelines	47.0	(44.4–49.5)	56.0	(50.4–61.6)	74.1	(69.7–78.4)	< 0.001^a,b,c^	1,441/400/766
Sedentary behaviour	9.9	(8.4–11.5)	15.0	(11.1–18.9)	27.6	(23.3–32.0)	< 0.001^a,b,c^	1,441/400/766
Subclinical atherosclerosis, N of CVD- (%)	76.6	(74.2- 79.6)	59.4	(54.8–63.9)	76.6	(70.7–82.5)	0.560^a^	923/210/299
Aortic stiffness, N of CVD- (%)	7.1	(5.7–9.1)	14.3	(9.0–19.6)	18.4	(13.3–23.5)	< 0.001^a,b,c^	928/209/294
Dyslipidaemia	89.9	(88.5–91.2)	92.1	(89.3–95.0)	96.0	(94.2–97.8)	< 0.001^b^	1,921/510/974
Hyperuricaemia	26.2	(24.3–28.2)	41.5	(37.3- 45.7)	45.3	(41.4–49.2)	< 0.001^a,b^	1,921/511/972

The descriptive variables are presented as mean ± SD, median [interquartile range (IQR)], or frequencies (in %) as appropriate. Linear trend was tested with an analysis of variance or a chi-square test as appropriate. Differences among groups of glucose metabolism status were tested with an independent t-test, or chi-square test, as appropriate. The comorbidities, classical complications and cardiometabolic risk factors are presented as age- and sex-adjusted prevalences (with normal glucose metabolism as reference category) in percentages with their 95% confidence interval (CI). Age- and sex-adjusted linear trend and differences among groups of glucose metabolism status were tested with a (multinomial) logistic regression analyses (with normal glucose metabolism or prediabetes and the lowest category as reference group).

^a^Prediabetes versus normal glucose metabolism, *P* < 0.05.

^b^Type 2 diabetes versus normal glucose metabolism, *P* < 0.05.

^c^Type 2 diabetes versus prediabetes, *P* < 0.05. Other *P*-values > 0.05.

^d^Diabetes duration was available in 664 individuals with type 2 diabetes; 133 of 975 individuals with type 2 diabetes had newly diagnosed type 2 diabetes.

Individuals with type 2 diabetes, as compared to individuals with NGM, had higher age- and sex-adjusted prevalences of thirteen out of seventeen comorbidities (i.e. dyspnoea, mild and severe limitations in mobility, any thyroid disorder, recent acute infection, polypharmacy, hearing loss, cognitive impairment, current depression, anxiety disorder, ocular hypertension, anaemia, non-alcoholic fatty liver disease, and obstructive sleep apnoea; Table 1); had higher age- and sex-adjusted prevalences of five out of six classical complications (i.e. all except for peripheral artery disease; Table 1); and had higher age- and sex-adjusted prevalences of nine out of ten cardiometabolic risk factors (i.e. current smoking, alcohol use, obesity, hypertension, non-compliance with physical activity guidelines, sedentary behaviour, aortic stiffness, dyslipidaemia, and hyperuricaemia; Table 1). In addition, individuals with prediabetes, as compared to individuals with NGM, had higher age- and sex-adjusted prevalences of seven out of seventeen comorbidities (i.e. dyspnoea, mild and severe limitations in mobility, prior malignancy, polypharmacy, hearing loss, non-alcoholic fatty liver disease, and obstructive sleep apnoea; Table 1); had higher age- and sex-adjusted prevalences of two out of six classical complications (i.e. coronary heart disease and sensory neuropathy; Table 1); and had higher age- and sex-adjusted prevalences of eight out of ten cardiometabolic risk factors (i.e. former smoking, obesity, hypertension, non-compliance with physical activity guidelines, sedentary behaviour, subclinical atherosclerosis, aortic stiffness, and hyperuricaemia; Table 1).

### Comorbidities

**Figure 1:**
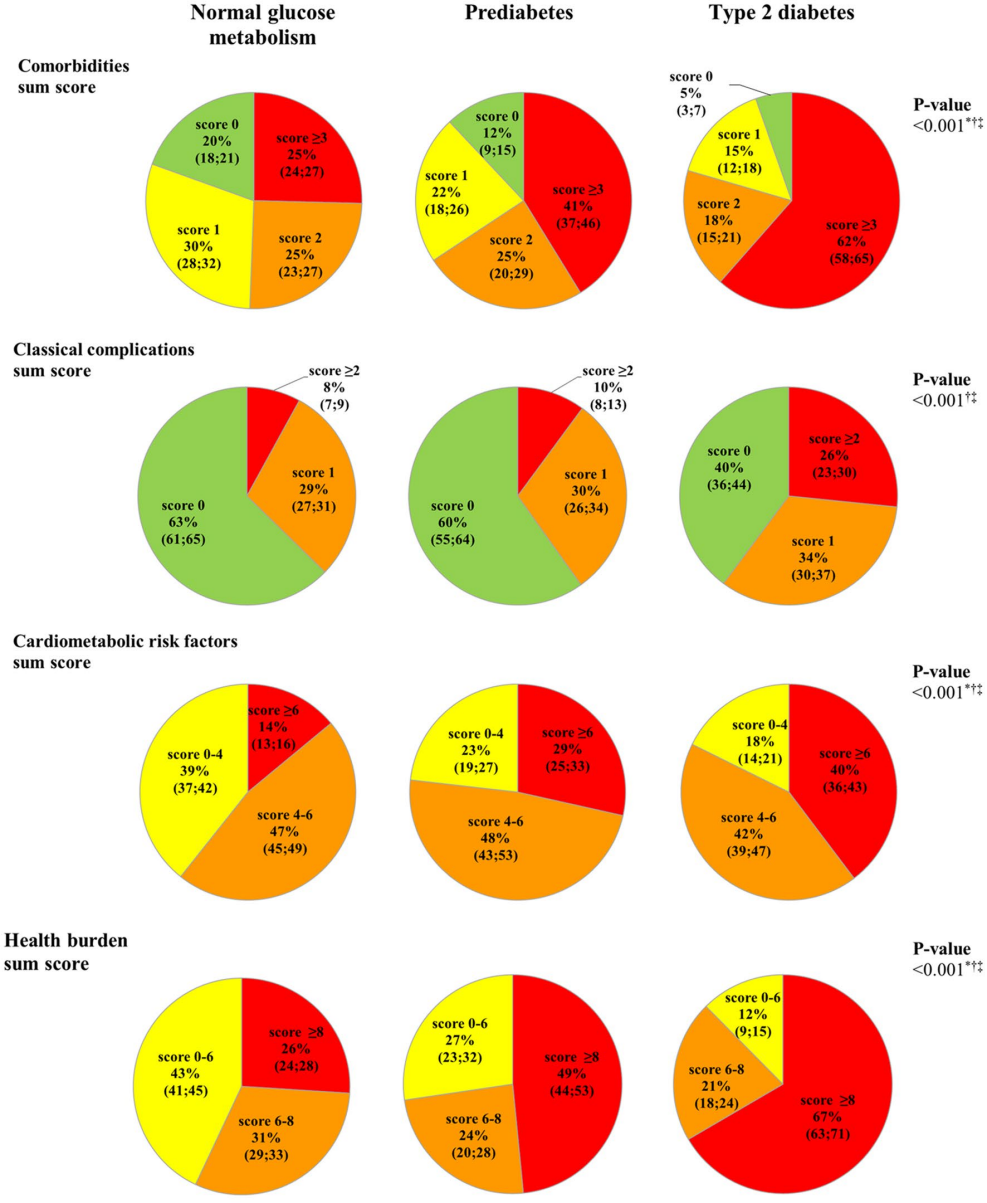
Age- and sex-adjusted sum scores of comorbidities, classical complications, cardiometabolic risk factors, and health burden according to glucose metabolism status. The health burden sum score (0–33) is the total of the number of comorbidities (0–17), classical complications (0–6), and cardiometabolic risk factors (0–10). The numeric categories of the comorbidities, classical complications, cardiometabolic risk factors and health burden sum scores are presented as age- and sex-adjusted frequencies (percentages with their 95% confidence intervals ; with normal glucose metabolism as reference category). Normal glucose metabolism (N = 1,924), prediabetes (N = 511), type 2 diabetes (N = 975). Age- and sex-adjusted linear trend and differences in categories of sum scores among groups of glucose metabolism status were tested with multinomial logistic regression analyses with normal glucose metabolism or prediabetes and the lowest sum score category as reference group. P-linear represents the trend with deteriorating glucose metabolism status for the highest sum score category, and ^*, †, ‡^ represent the differences between groups of glucose metabolism status per highest sum score category. *Prediabetes versus normal glucose metabolism *P* < 0.001; ^†^type 2 diabetes versus normal glucose metabolism *P* < 0.001; ^‡^type 2 diabetes versus prediabetes *P* < 0.005. Other *P*-values > 0.05. Five individuals with normal glucose metabolism had a cardiometabolic risk factors sum score of zero. No individual had a health burden sum score of zero.

Table 2 shows the sum scores for comorbidities, classical complications, cardiometabolic risk factors and health burden for individuals with type 2 diabetes, prediabetes, and NGM. After adjustment for age and sex, type 2 diabetes and prediabetes, as compared to NGM, were associated with a greater mean comorbidities sum score (P-linear < 0.001, Table 2). In addition, individuals with type 2 diabetes and prediabetes, as compared to individuals with NGM, more often had a comorbidities sum score of ≥ 3 (age-and sex-adjusted frequencies (95% confidence interval): 61.5% (57.6; 65.4) and 41.2% (36.5; 45.9), versus 25.4% (23.5; 27.4), Fig. 1), and less often had a comorbidities sum score of 0 (5.4% (3.5; 7.4) and 12.0% (8.7; 15.2), versus 19.5% (17.8; 21.3)).

**Table 2:** Sum scores of comorbidities, classical complications, cardiometabolic risk factors, and health burden according to glucose metabolism status.

	Normal glucose metabolism	Prediabetes	Type 2 diabetes	P-linear
**Comorbidities sum score (0–17)**				
Mean (SD)	1.76 (1.45)	2.43 (1.65)	3.33 (1.97)	< 0.001^a,b,c^
Mean (SE), adjusted for age and sex	1.81 (0.04)	2.38 (0.07)	3.26 (0.05)	< 0.001^a,b,c^
**Classical complications sum score (0–6)**^d^				
Mean (SD)	0.47 (0.70)	0.61 (0.77)	1.14 (1.08)	< 0.001^a,b,c^
Mean (SE), adjusted for age and sex	0.52 (0.02)	0.57 (0.04)	1.06 (0.03)	< 0.001^b,c^
**Cardiometabolic risk factors sum score (0–10)**				
Mean (SD)	3.96 (1.39)	4.78 (1.49)	5.21 (1.50)	< 0.001^a,b,c^
Mean (SE), adjusted for age and sex	4.07 (0.03)	4.72 (0.06)	5.03 (0.05)	< 0.001^a,b,c^
**Health burden sum score (0–33)**				
Mean (SD)	6.19 (2.40)	7.82 (2.74)	9.69 (3.14)	< 0.001^a,b,c^
Mean (SE), adjusted for age and sex	6.40 (0.06)	7.67 (0.11)	9.34 (0.08)	< 0.001^a,b,c^

N = 3,410; 1,924/511/975. Data are presented as mean (standard deviation) or adjusted mean (standard error). Crude linear trends and differences in sum scores among groups of glucose metabolism status were tested with an analysis of variance or an independent t-test as appropriate. Age-and sex-adjusted mean values (standard error) were calculated with an analysis of covariance. Age- and sex-adjusted linear trends and differences in sum scores among groups of glucose metabolism status were tested with linear regression analyses.

^a^Prediabetes versus normal glucose metabolism *P* < 0.005;

^b^Type 2 diabetes versus normal glucose metabolism *P* < 0.001.

^c^Type 2 diabetes versus prediabetes *P* < 0.05. Other *P*-values > 0.05.

^d^Numbers (normal glucose metabolism/ prediabetes/ type 2 diabetes) for the analyses with the classical complications sum score are 1,924/511/974.

### Classical complications

After adjustment for age and sex, type 2 diabetes, as compared to NGM, was associated with a greater mean classical complications sum score (P-linear < 0.001, Table 2). In addition, individuals with type 2 diabetes, as compared to individuals with NGM, more often had a classical complications sum score of ≥ 2 (26.6% (23.1; 30.1) versus 8.0% (6.9;9.3));  Fig. 1), and less often had a classical complications sum score of 0 (39.8% (35.8;43.8) versus 62.7% (60.5; 64.8)). In contrast, after adjustment for age and sex, prediabetes, as compared to NGM, was not associated with a greater mean classical complications sum score, and individuals with prediabetes, as compared to individuals with NGM, did not significantly more often have a classical complications sum score of ≥ 2 (Table 2 and Fig. 1). In addition, individuals with prediabetes, as compared to individuals with NGM, equally often had a classical complications sum score of 0 (59.8% (55.3; 64.4) versus 62.7% (60.5; 64.8)).

### Cardiometabolic risk factors

After adjustment for age and sex, type 2 diabetes and prediabetes, as compared to NGM, were associated with a greater mean cardiometabolic risk factor sum score (P-linear < 0.001, Table 2). In addition, individuals with type 2 diabetes and prediabetes, as compared to individuals with NGM, more often had a cardiometabolic risk factors sum score of ≥ 6 (39.7% (35.9; 43.4) and 28.5% (24.5; 32.6), versus 14.0% (12.5; 15.6),  Fig. 1), and less often had a cardiometabolic risk factors sum score of 0–4 (17.7 (14.4; 21.0) and 23.2 (19.0;27.4) versus 39.4 (37.2;41.5)).

### Health burden

After adjustment for age and sex, type 2 diabetes and prediabetes, as compared to NGM, were associated with a greater mean health burden sum score (P-linear < 0.001, Table 1). In addition, individuals with type 2 diabetes and prediabetes, as compared to individuals with NGM, more often had a health burden sum score of ≥ 8 (66.6% (62.7; 70.5) and 48.4% (43.7; 53.1), versus 26.0% (24.1; 28.0),  Fig. 1), and less often had a health burden sum score of 0–6 (12.4% (9.4; 15.3) and 27.3% (22.9; 31.8) versus 43.0% (40.8; 45.3)). For details on the distribution of the sum scores of comorbidities, classical complications, cardiometabolic risk factors and health burden, see supplementary Fig. S1.

### Sensitivity analyses

Results were similar when we repeated the analyses in individuals with complete data on comorbidities (N = 906), classical complications (N = 2,286), and cardiometabolic risk factors (N = 2,539) and health burden (N = 653; Table S1; for details on study populations and missing values see supplementary Fig. S2). Note that the study population with complete data and that with missing values were comparable with regard to the prevalences of classical complications, comorbidities and cardiometabolic risk factors, as compared to the total study population (Table S2). Analyses stratified for sex are shown in Tables S3–S5 and in percentages in supplementary Figs. S3 and S4. For type 2 diabetes and prediabetes, all associations with categories of sum scores were similar in women and men (all P_interaction_ > 0.10). In linear regression analyses, for type 2 diabetes, the association with the comorbidities sum score was slightly stronger in women (regression coefficient interaction term (β) and 95% confidence interval: 0.37 (0.10; 0.63) point, P_interaction_ = 0.006); with the classical complications sum score, slightly stronger in men (0.18 (0.05; 0.31)), P_interaction_ = 0.007); and with the cardiometabolic risk factors sum score, similar in women and men (P_interaction_ > 0.10). Results with regard to health burden were similar in women and men (P_interaction_ > 0.10). In linear regression analyses, for prediabetes, the association with the cardiometabolic risk factor sum score was slightly stronger in men (0.26 (-0.01; 0.53)) point, (P_interaction_ = 0.06), and associations with comorbidities, classical complications, and health burden were similar in women and men (all P_interaction_ > 0.10).

If we specified prediabetes into individuals with impaired fasting glucose and impaired glucose tolerance, as compared to NGM and after adjustment for age and sex, individuals with impaired glucose tolerance had higher prevalences of five comorbidities, two classical complications and eight cardiometabolic risk factors; and individuals with impaired fasting glucose had higher prevalences of three comorbidities and five cardiometabolic risk factors (Table S6). Moreover, if we investigated the health burden prevalences according to diabetes type 2 disease duration, after adjustment for age and sex and as compared to individuals with newly diagnosed type 2 diabetes, individuals with type 2 diabetes with longer disease duration had higher prevalences of five comorbidities in addition to four classical complications and six cardiometabolic risk factors (P-linear < 0.001; Table S7).

As some health burden variables were age-specified, we explored potential overadjustment for age by repeating analyses with age-independent definitions of sedentary behaviour, atherosclerosis and aortic stiffness; results indicated that overadjustment by age was not present (Tables S8 and S9). Results with regard to health burden and cardiometabolic risk factors sum scores were similar after additional adjustment for the potential confounders mean arterial blood pressure and heart rate (Table S10).

### Comparison with age

In analyses with use of age-independent cut-offs in the definition of health burden variables, the association between age and health burden was 0.09 (0.08; 0.10) per one year of ageing. Thus, age- and sex-adjusted associations of type 2 diabetes and prediabetes with health burden were comparable to 32 (30; 34), and 14 (11; 16) years of ageing, respectively (Table S9).

## Discussion

Health burden in type 2 diabetes and prediabetes, as defined and quantified by the presence of comorbidities, classical complications, and cardiometabolic risk factors, is substantially greater than in NGM. Three novel findings serve to illustrate this. Firstly, after adjustment for age and sex, 62% of individuals with type 2 diabetes had three or more comorbidities, as compared to 25% of individuals with NGM. Secondly, 67% of individuals with type 2 diabetes had a health burden sum score of 8 or more (out of a maximum of 31), as compared to 26% of individuals with NGM. Thirdly, the excess of comorbidities and health burden sum score in prediabetes was about one third to one half of that in type 2 diabetes. Health burden in individuals 40 to 75 years of age with type 2 diabetes or prediabetes was comparable to respectively 32 and 14 years of ageing. Taken together, these data underline the concept that greater health burden in both type 2 diabetes and prediabetes is substantial, and can be attributed, in part, to the presence of non-classical comorbidities in addition to classical complications and cardiometabolic risk factors. Moreover, these findings support the hypothesis that hyperglycaemia can play a role in the development of a greater health burden early in the course of type 2 diabetes and indeed in prediabetes^5^, notably in those with impaired glucose tolerance.

Previous population-based studies have reported, in individuals with versus without diabetes, and to a lesser extent in individuals with versus without prediabetes, greater prevalences of individual or a limited set of comorbidities, classical complications and/or cardiometabolic risk factors (see the supplementary material for an overview of the literature). In addition, previous population-based studies in individuals with versus without diabetes have reported greater count measures of health burden, including a broad range of comorbidities, in diabetes. For example, 29.0% of individuals with diabetes versus 9.7% without diabetes had a Charlson comorbidity index of ≥ 3^11^, and the mean number of comorbidities (SD) was 3.7 ± 2.4 versus 2.2 ± 2.0^14^. Count measures of health burden including comorbidities have not been previously studied in prediabetes. Our study extends previous findings to quantification of health burden in type 2 diabetes and prediabetes through a detailed study of a diverse set of comorbidities in addition to classical complications and/or cardiometabolic risk factors; to a population-based cohort of individuals aged 40–75 years with both type 2 diabetes and prediabetes as compared to NGM; and to comparisons of health burden sum scores between these groups adjusted for age and sex.

Although we aimed to investigate comorbidities, classical complications and cardiometabolic risk factors in a comprehensive manner^5,16^, we made inevitable choices, which may be questioned. Firstly, however, the 17 comorbidities investigated are well-known disease outcomes according to the international classification of disease^31^, with the exception of dyspnoea (which we used as a proxy for pulmonary and cardiac disease) and polypharmacy. Nevertheless, dyspnoea symptoms and the use of five or more chronic medications reasonably may contribute to health burden. Secondly, we added ten cardiometabolic risk factors to the health burden sum score, which may contribute to health burden through disease awareness and/or the use of medication (e.g. hypertension and dyslipidaemia), or may have prognostic implications (e.g. subclinical atherosclerosis). Thirdly, data on the presence of other than self-reported cancers were not available, which may have caused us to underestimate health burden due to the healthy participant effect. Lastly, we had no data available on some important other diabetes-associated disease outcomes such as nonhepatic digestive system disorders^5^, periodontal disease^16^, psoriasis^22^, osteopenia and osteoporosis^23^, hypogonadism^24^ and heart failure^32^. However, the addition of these disease outcomes would have resulted in a greater health burden in type 2 diabetes as compared to NGM^5,16^, and thus will likely have caused us, if anything, to underestimate the differences between type 2 diabetes and NGM observed.

The descriptive and cross-sectional design of our study with adjustment for only age and sex does not allow us to further elucidate underlying mechanisms in the association between (pre)diabetes and greater comorbidity and health burden. However, the mechanisms that underlie these associations, although they may be multifactorial, quite possibly include hyperglycaemia. Previous prospective findings (with extensive adjustments for potential confounders) have shown that excess mortality in (pre)diabetes is closely associated with level of glycaemia and is attributable to a considerable extent by death not due to cardiovascular disease or cancer^5^, and thus in part to the comorbidities considered here. Therefore, further research on the natural history of hyperglycaemia and occurrence of comorbidities is warranted, as are interventions to investigate whether reducing hyperglycaemia can reduce comorbidities and health burden.

The association between type 2 diabetes and sum score of comorbidities was slightly stronger in women, while that with classical complications was slightly stronger in men. These findings might be explained by a differential impact of biological and lifestyle factors on risk of comorbidities and classical complications of diabetes in women and men^33^. Analyses of sum scores, however, do not exclude differences at the level of single outcomes (which we had limited power to detect). Indeed, women with type 2 diabetes have been reported to have greater increases of myocardial infarction and stroke mortality than men with type 2 diabetes, as compared to individuals without type 2 diabetes^33^.

Strengths of our study include its population-based design with oversampling of individuals with type 2 diabetes, which enabled an accurate comparison of individuals with type 2 diabetes and NGM; consideration of prediabetes; and the use of extensive phenotyping.

Our study also had limitations. Firstly, the focus of our study was to quantify health burden and not to investigate potential causal relationships between (pre)diabetes and health burden. Therefore, further prospective research of health burden determinants in (pre)diabetes is needed. Secondly, we made the conservative assumption that missing data indicated absence of an abnormal result, which may have caused us to underestimate the differences observed. However, results of analyses in the subpopulation with complete data were similar to those in the total study population. Thirdly, we may have underestimated differences in health burden between (pre)diabetes and NGM because we did not consider some disease outcomes (e.g. nonhepatic digestive system disorders^5^) that are known to be more prevalent in (pre)diabetes than in NGM; because the healthy participant effect (i.e. sicker potential participants are less likely to participate) is likely to be stronger in (pre)diabetes than in NGM; and because, among individuals with type 2 diabetes, those with poor glycaemic control are likely to have been underrepresented to some extent. Lastly, data were obtained in a Caucasian population and therefore it remains to be established whether these quantitative results can be generalized to other ethnicities.

In conclusion, our population-based study shows, independently of age and sex, a considerably greater health burden in not only type 2 diabetes, but also prediabetes, which to an important extent is related to non-classical comorbidities in addition to classical complications and cardiometabolic risk factors. These findings emphasize the need for health care providers’ awareness of comorbidities in individuals with (pre)diabetes. Future studies should focus on the natural history of hyperglycaemia and occurrence of comorbidities, and on interventions to investigate whether reducing hyperglycaemia can reduce comorbidities and health burden.

## Supplementary Information

Supplementary Information.

## Data Availability

Data are unsuitable for public deposition due to ethical restrictions and privacy of participant data according to the approved study protocol by the institutional medical ethical committee (Medisch-ethische toetsingscommissie azM/UM, NL31329.068.10) and the Minister of Health, Welfare and Sports of the Netherlands (Permit 131,088–105,234-PG). Nevertheless, data are available from The Maastricht Study for any interested researcher who meets the criteria for access to confidential data. The Maastricht Study Management Team (research.dms@mumc.nl) and the corresponding author (C. Stehouwer) may be contacted to request data.

## Abbreviations

CI: Confidence interval
CVD: Cardiovascular disease
CHD: Coronary heart disease
eGFR: Estimated glomerular filtration rate
HDL: High density lipoprotein
LDL: Low density lipoprotein
NGM: Normal glucose metabolism
