# Supplementary material for: Health burden in type 2 diabetes and prediabetes in The Maastricht Study
Source: Sci Rep. 2022 May 5;12:7337. doi: 10.1038/s41598-022-11136-5 (PMC9072328; doi:10.1038/s41598-022-11136-5)
Supplement: Supplementary file 1 — Supplementary Information. [file 41598_2022_11136_MOESM1_ESM.pdf]

ONLINE SUPPLEMENT TO:

Health burden in type 2 diabetes and prediabetes – The Maastricht Study -

Authors

Marja G.J. Veugen[1,2] (ORCID 0000-0003-1665-6090), Veronica G. Onete[1,2], Ronald M.A. Henry[1,2,3], Hans-Peter Brunner-La Rocca[2,4], Annemarie Koster[5,6], Pieter C. Dagnelie[1,2], Nicolaas C. Schaper[1,2,5], Simone J.S. Sep[1,2,7], Carla J.H. van der Kallen[1,2], Martin P.J. van Boxtel[8], Koen D Reesink[2,9], Johannes S. Schouten[10], Hans H.C.M. Savelberg[11], Sebastian Köhler[8], Frans R. Verhey[8], Joop P. W. van den Bergh[12,13,14], Miranda T. Schram[1,2,3], Coen D.A. Stehouwer[1,2]

[1]Department of Internal Medicine, Maastricht University Medical Centre +, Maastricht, the Netherlands; [2]CARIM School for Cardiovascular Diseases, Maastricht University, Maastricht, the Netherlands; [3]Heart and Vascular Centre, Maastricht University Medical Centre +, Maastricht, The Netherlands; [4]Department of Cardiology, Maastricht University Medical Centre +, Maastricht, the Netherlands; [5]CAPHRI Care and Public Health Research Institute, Maastricht University, Maastricht, the Netherlands; [6]Department of Social Medicine, Maastricht University, Maastricht, the Netherlands; [7]Adelante, Centre of Expertise in Rehabilitation and Audiology, Hoensbroek, the Netherlands; [8]Department of Psychiatry and Neuropsychology and MHeNS School for Mental Health and Neuroscience, Maastricht University, Maastricht, the Netherlands; [9]Department of Biomedical Engineering, Maastricht University Medical Centre +, Maastricht, the Netherlands; [10]Department of Ophthalmology, Maastricht University Medical Centre +, Maastricht, the Netherlands, and, Canisius-Wilhelmina Hospital, Nijmegen, the Netherlands; [11]Department of Human Movement Sciences, Maastricht University, Maastricht, the Netherlands; [12]Department of Family Medicine, Maastricht University, Maastricht, The Netherlands; [13]Department of Internal Medicine, Subdivision of Rheumatology, Maastricht University Medical Centre+, Maastricht, The Netherlands; [14]Department of Internal Medicine, Subdivision of Endocrinology, VieCuri Medical Center, Venlo, The Netherlands.

SUPPLEMENTARY MATERIAL: OVERVIEW OF CURRENT LITERATURE

Evidence before this study

We searched PubMed with the search terms "type 2 diabetes mellitus", "type 2 diabetes" "diabetes", "impaired glucose metabolism", "impaired fasting glucose", "impaired glucose tolerance", "prediabetes", "normal glucose metabolism", or "glucose metabolism status"; and "normal glucose metabolism", "glucose metabolism status", "normal glucose", "without diabetes", "no diabetes", "impaired glucose metabolism", or "prediabetes"; and "health burden", "burden of disease", "complications", "comorbidities", "multimorbidity", "polymorbidity", "co-morbidity", "comorbidity", "risk factors", "symptom distress score", "DCSI", or "diabetes complication severity index", appropriate search terms for the individual comorbidities, classical complications and cardiometabolic risk factors, and population-based study (see supplementary material) before February 1st, 2022 and after February 1st, 2008 (to find relevant studies in the last 10 years at the start of the study), to find studies that had assessed health burden defined as comorbidities, classical complications, and(or) cardiometabolic risk factors in individuals with (pre)diabetes and normal glucose metabolism in a population-based setting (for the complete search see the supplementary material) in a mainly Caucasian adult population. In addition, we added a limited number (n=5) of studies we found by studying the literature or as a reference reported in an included study.

We identified forty-eight population-based studies (see supplementary material) that reported data on health burden of comorbidities, classical complications, and/or cardiometabolic risk factors in individuals with and without (pre)diabetes. These studies reported results consistent with the concept that health burden in diabetes is greater by showing higher prevalences of individual or a limited set of comorbidities, classical complications and/or cardiometabolic risk factors in (pre)diabetes or an association of (pre)diabetes with individual or a limited set of comorbidities, classical complications and/or cardiometabolic risk factors. However, only five studies reported a count measure to quantify health burden in diabetes, of which only three comprehensively investigated a diverse set of comorbidities in addition to classical complications and/or cardiometabolic risk factors, and none of those studies considered prediabetes. Furthermore, given the heterogeneous way of data collection no quantitative measure can be drawn from studies that investigated individual disease outcomes and health determinants to quantify the health burden of comorbidities in addition to classical complications and/or cardiometabolic risk factors. Therefore, the quantitative (pre)diabetes-associated health burden including comorbidities in a population-based setting is not known.

Search Pubmed

Search ("Humans"[Mesh]) AND ((("Diabetes Mellitus, Type 2"[Mesh] OR "Prediabetic State"[Mesh] OR "Glucose Intolerance"[Mesh] OR "Glucose Metabolism Disorders"[Mesh]) OR "type 2 diabetes mellitus"[All Fields] OR "type 2 diabetes"[All Fields] OR "diabetes"[All Fields] OR "impaired glucose metabolism"[All Fields] OR "prediabetes"[All Fields] OR "glucose metabolism status"[All Fields] OR "impaired fasting glucose"[All Fields] OR "impaired glucose tolerance"[All Fields]) AND ("normal glucose metabolism"[All Fields] OR "glucose metabolism status"[All Fields] OR "normal glucose"[All Fields] OR "without diabetes"[All Fields] OR "no diabetes"[All Fields] OR "without"[All Fields]) AND ("Morbidity"[Mesh] OR "Diabetes Complications"[Mesh] OR "Comorbidity"[Mesh] OR "Risk Factors"[Mesh] OR "health burden"[All Fields] OR "Burden of disease"[All Fields] OR "complications"[All Fields] OR "comorbidities"[All Fields] OR "multimorbidity"[All Fields] OR "co-morbidity"[All Fields] OR "comorbidity"[All Fields] OR "risk factors"[All Fields] OR "symptom distress score"[All Fields] OR "DCSI"[All Fields] OR "diabetes complication severity index"[All Fields] OR polymorbidity[All Fields] OR ("Dyspnea"[Mesh] OR "dyspnea"[All Fields] OR "Respiratory Tract Diseases"[Mesh] OR "respiratory disease"[All Fields] OR "respiratory diseases"[All Fields] OR "Pulmonary Disease, Chronic Obstructive"[Mesh] OR "chronic obstructive pulmonary disease"[All Fields] OR "Asthma"[Mesh] OR "asthma"[All Fields]) OR ("Mobility Limitation"[Mesh] OR (limitation[All Fields] AND mobility[All Fields]) OR (limitations[All Fields] AND mobility[All Fields]) OR "mobility limitation"[All Fields]) OR ("Neoplasms"[Mesh] OR "malignancy"[All Fields]) OR ("Thyroid Diseases"[Mesh] OR "thyroid disorder"[All Fields] OR "thyroid dysfunction"[All Fields]) OR ("Fractures, Bone"[Mesh] OR "fracture"[All Fields] OR "fractures"[All Fields]) OR ("Communicable Diseases"[Mesh] OR "infectious disease"[All Fields] OR "infectious diseases"[All Fields] OR "infection"[All Fields] OR "infections"[All Fields]) OR ("Polypharmacy"[Mesh] OR "polypharmacy"[All Fields]) OR ("Hearing Loss"[Mesh] OR "hearing loss"[All Fields]) OR ("Cognitive Dysfunction"[Mesh] OR "cognitive impairment"[All Fields] OR "cognitive decline"[All Fields] OR "cognitive dysfunction"[All Fields]) OR ("Depression"[Mesh] OR "Depressive Disorder"[Mesh] OR "depression"[All Fields] OR "depressive disorder"[All Fields]) OR ("Anxiety Disorders"[Mesh] OR "anxiety disorder"[All Fields] OR "anxiety"[All Fields] OR "anxiety disorders"[All Fields]) OR ("Mental Health"[Mesh] OR "Mental Disorders"[Mesh] OR "mental health"[All Fields] OR "mental disorder"[All Fields]) OR ("Atrial Fibrillation"[Mesh] OR "atrial fibrillation"[All Fields] OR "Atrial Flutter"[Mesh] OR "atrial flutter"[All Fields]) OR ("Ocular Hypertension"[Mesh] OR "Glaucoma"[Mesh] OR "ocular hypertension"[All Fields] OR "glaucoma"[All Fields]) OR ("Anemia"[Mesh] OR "anemia"[All Fields] OR "anaemia"[All Fields] OR "Anemia, Iron-Deficiency"[Mesh] AND "Folic Acid Deficiency"[Mesh]) OR ("Sleep Apnea, Obstructive"[Mesh] OR "obstructive sleep apnea"[All Fields]) OR ("Liver Diseases"[Mesh] OR "liver disease"[All Fields] OR "liver diseases"[All Fields]) OR ("Gastrointestinal Diseases"[Mesh] OR "gastrointestinal diseases"[All Fields] OR "gastrointestinal disease"[All Fields]) OR ("Periodontal Diseases"[Mesh] OR "periodontal diseases"[All Fields] OR "periodontal disease"[All Fields] OR "periodontitis"[All Fields]) OR (“Psoriasis"[Mesh] OR “psoriasis”[All Fields]) OR ((((((("Coronary Disease"[Mesh] OR "coronary heart disease" OR "coronary disease")) OR ("Cerebrovascular Disorders"[Mesh] OR "cerebrovascular disease" OR "cerebral vascular disease" OR "stroke")) OR ("Peripheral Arterial Disease"[Mesh] OR "peripheral arterial disease" OR "peripheral artery disease")) OR ("Cardiovascular Diseases"[Mesh] OR "cardiovascular disease" OR "cardiovascular diseases")) OR

("Diabetic Retinopathy"[Mesh] OR "diabetic retinopathy")) OR ("Renal Insufficiency, Chronic"[Mesh] OR "chronic kidney disease" OR "renal insufficiency" OR "albuminuria")) OR ("sensory neuropathy" OR "diabetic sensory neuropathy") OR ((((((((("Smoking"[Mesh] OR "smoking")) OR ("Alcohol Drinking"[Mesh] OR "alcohol use" OR "alcohol drinking")) OR ("Obesity"[Mesh] OR "obesity")) OR ("Hypertension"[Mesh] OR "hypertension")) OR ("Exercise"[Mesh] OR "physical activity")) OR ("Sedentary Lifestyle"[Mesh] OR "sedentary behaviour" OR "sedentary behavior")) OR ("Atherosclerosis"[Mesh] OR "atherosclerosis")) OR ("Vascular Stiffness"[Mesh] OR "arterial stiffness" OR "vascular stiffness" OR "aortic stiffness")) OR ((("Dyslipidemias"[Mesh] OR "dyslipidemia" OR "dyslipidaemia")) OR "Cholesterol"[Mesh])) OR ("Hyperuricemia"[Mesh] OR "hyperuricemia" OR "hyperuricaemia" OR "uric acid")) AND (("population-based"[All Fields] OR "population based"[All Fields] OR "general population"[All Fields] OR "community-based"[All Fields] OR "community based"[All Fields] OR "community-dwelling"[All Fields] OR "community dwelling"[All Fields]))) Filters: published in the last 10 years

Table S-A. Overview of population-based studies of comorbidities, classical complications and/or cardiometabolic risk factors in individuals with and without (pre)diabetes

Reference	Data source	Study population, N	Comorbidities, classical complications, cardiometabolic risk factors specified	Findings in individuals with versus without (mainly) type 2 diabetes‡	Findings in individuals with versus without prediabetes	Adjustments reported	Conclusion paper
Zghebi et al. (2020)[1]	Population-based cohort study with primary care and linked hospitalization data in individuals aged ≥ 16 years with T2D and matched comparators; Read and ICD-10 codes.	T2D 108,588 / 528,667 matched comparators without T2D	CM: asthma, chronic obstructive pulmonary disease, hypothyroidism, osteoarthritis, anxiety, depression, schizophrenia, epilepsy, atrial fibrillation, congestive heart failure, cancer. CC: chronic kidney disease, peripheral vascular disease, myocardial infarction, transient ischaemic attack, stroke. CMRF: hypertension, hyperlipidaemia. Count measure: 0,1,2,3,4, or ≥5 CM/CC/CMRF.	0 CM/CC/CMRF 24% and 44%. 1 CM/CC/CMRF: 27% and 24%. 2 CM/CC/CMRF: 20% and 15%. 3 CM/CC/CMRF: 14% and 8%. 4 CM/CC/CMRF: 7% and 4%. ≥5 CM/CC/CMRF: 4% and 2%.	n/a	Annual prevalence %, odds ratio, count score: comparators were categorized by age, gender and social deprivation.	In T2D vs without: greater health burden of all investigated comorbidities, classical complications and cardiometabolic risk factors; prediabetes data NA; count measure available: investigated eleven CMs, five CCs, two CMRFs.
Iranfar et al. (2018)[2]	Serial cross-sectional design with Behavioral Risk Factor Surveillance System (BRFSS) survey data in individuals aged ≥18 years; telephone interview (self-	Diabetes 215,441 / prediabetes 63,657 / NGM 1,420,656.	CM: depressive disorder, cancer. CC: cardiovascular disease (≥1, considered chronic heart disease, heart attack, stroke), kidney disease. CMRF: obese, smoking, regular alcohol use; regular exercise.	CM: depressive disorder 26.1% and 17.7%; cancer 19.3% and 11.7%. CC: cardiovascular disease 25.3% and 8.7%; kidney disease 8.9% and 2.7%. CMRF: obese 54.0% and 29.1%; smoking 52.7% and 43.7%; regular alcohol use 2.6% and 6.3%; regular exercise 62.8% and 76.1%.	CM: depressive disorder 27.5% and 17.7%; cancer 17.3% and 11.7%. CC: cardiovascular disease 15.4% and 8.7%; kidney disease 3.7% and 2.7%. CMRF: obese 47.6% and 29.1%; smoking 5.4% and 6.3%; regular alcohol use 5.4% and 6.3%; regular	Prevalence % unadjusted; age- and sex-adjustment NA.	In diabetes and prediabetes vs. NGM: greater health burden of depressive disorder, cancer, cardiovascular disease, kidney disease and CMRFs; age-and sex-adjustment NA; count measure NA (questionnaire contained eight chronic conditions and four CMRFs);

	reported diagnoses).				exercise 70.4% and 76.1%.		investigated two CMs, ≥1 cardiovascular disease and kidney disease as CCs and four CMRFs.
Castro-Rodriguez et al. (2016)[3]	Toledo Study of Healthy Aging (TSHA), prospective cohort study in individuals aged ≥65 years (98.1% community-dwelling, 1.9% institutionalized); home interviews (self-reported diagnoses).	T2D 363 / without T2D 1,462.	CM/CC: Charlson index (CCI 0/1/2/3/>3). CC: cardiovascular disease (≥1, considered ischemic heart disease and stroke), cerebrovascular disease.	CM/CC: Charlson index 0/49.0/ 22.0/12.3/16.7% and 57.4 /20.0/12.9/4.5/5.2% (p<0.0001). CC: cardiovascular disease 23.7% and 11.1% (p<0.0001); cerebrovascular disease 7.7% and 4.7% (p=0.0193).	n/a	Prevalence % unadjusted; age- and sex-adjustment NA.	In T2D vs. without: greater health burden of Charlson index, cardiovascular disease and cerebrovascular disease; age- and sex-adjustment NA; prediabetes data NA; count measure available (questionnaire contained sixteen conditions and two CMRFs); investigated Charlson index, and ≥1 cardiovascular disease and cerebrovascular disease as CCs.
Ervasti et al. (2016)[4]	Nationwide population-based Insurance Medicine All-Sweden (IMAS) research database of which individuals aged 25–59 years were selected with incident diabetes in 2006 and as reference 1% random sample without diabetes; hospital records, ICD-codes.	Incident diabetes 14,198 / without diabetes 39,204.	CM: depression, CC: cardiovascular disease (≥1, considered cardiac and cerebrovascular disease) CMRF: hypertension.	CM: prevalent depression 17.8% and 9.3%; incident depression 7.6% and 5.8%. CC: prevalent cardiovascular disease (≥1) 15.9% and 7.1%; incident cardiovascular disease 11.7% and 6.9%. CMRF: prevalent hypertension 9.0% and 0.9%, incident hypertension 13.4% and 2.3%.	n/a	Prevalence % unadjusted (comparisons were not tested); age- and sex-adjustment NA.	In diabetes vs. without: greater health burden of depression, cardiovascular disease and hypertension in diabetes; prediabetes data NA; age-and sex-adjustment NA; count measure NA; investigated three CMs (also other psychiatric, and musculoskeletal disorders); for ≥1 cardiovascular disease as CC and one CMRF.
Graham et al. (2015)[5]	English Longitudinal Study of Aging based on the Health Survey	T2D 346 / prediabetes 893 / NGM 2,929.	CM: ≥1 non-cardiovascular condition (8 considered: asthma, chronic lung disease,	CM: ≥1 non-cardiovascular condition 60.7% and 46.1%; high depressive symptoms 36.3% and 24.6%.	CM: ≥1 non-cardiovascular condition 53.7% and 46.1% (P<0.001), RR (95% CI) 1.08 (0.91-1.29);	Prevalence % unadjusted (comparisons between T2D and NGM were not tested); only age	In T2D vs. NGM: greater health burden of ≥1 non-cardiovascular condition, depression, ≥1 cardiovascular condition

	for England in adults aged 50–75 years; questionnaire (self-reported diagnoses) and clinical examination by nurse.		arthritis, cancer or a malignant tumor, osteoporosis, Parkinson's disease, Alzheimer's disease, and dementia or another serious memory impairment), high depressive symptoms.<br>CC: ≥1 cardiovascular condition (7 considered: hypertension, angina, congestive heart failure, abnormal heart rhythm, heart murmur, heart attack, and stroke).<br>CMRF: smoking, (never/former/current) physical activity (none/moderate/vigorous), alcohol use (never/rarely/1-4 times a week/≥5times a week), obesity.	CC: ≥1 cardiovascular condition 81.5% and 42.2%.<br>CMRF: smoking 27.9/55.9/16.3% and 38.9/47.1/14.1%; physical activity 30.4/48.3/21.3% and 13.5/50.5/36.1%; alcohol use 16.7/34.5/34.8/14.1% and 7.1/24.4/41.9/26.6%; obesity 52.3% and 23.3%.	high depressive symptoms 27.3% and 24.6% (P<0.001), RR 0.99 (0.93-1.06).<br>CC: ≥1 cardiovascular condition 49.7% and 42.2% (P<0.001), RR 1.10 (0.93-1.30).<br>CMRF: smoking 34.7/41.1/24.2% and 38.9/47.1/14.1% (P<0.001), RR former 0.99 (0.082-1.18)/ RR current 2.21 (1.74-2.80); physical activity 20.3/50.9/28.8% and 13.5/50.5/36.1% (P<0.001), RR moderate 0.84 (0.67-1.06); RR vigorous 0.80 (0.62-1.04); alcohol use 11.9/30.6/40.2/17.3% and 7.1/24.4/41.9/26.6% (P<0.001), RR 1-4 times a week 0.76 (0.56-1.02) RR ≥5times a week 0.51 (0.37-0.71);<br>obesity 37.0% and 23.3% (P<0.001), RR per kg/m^2 increase BMI 1.08 (1.06-1.10).	and sex NA; RR adjusted for age, sex, marital status, wealth, educational qualifications, employment, smoking, physical activity level, alcohol in the past 12 months, BMI, self-related health, and CESD symptoms, presence of non-cardiovascular and/or cardiovascular condition as appropriate.	and cardiometabolic risk factors.<br>In prediabetes vs. NGM: greater health burden of ≥1 non-cardiovascular condition, depression, ≥1 cardiovascular condition and cardiometabolic risk factors, which lies in between the health burden in T2D vs. NGM, but did not remain significant after adjustment except for current smoking, alcohol use ≥5times a week and obesity; only age-and sex-adjustment NA; count measure NA; investigated eight CMs for ≥1 non-cardiovascular condition, depression as additional CM, six conditions (three CCs, three CMs) and hypertension (CMRF) for ≥1 cardiovascular condition, and in addition four CRMFs.
Comino et al. (2015)[6]	Sax's Institute 45 and Up Study in random samples of adults aged ≥45 years registered on medicare Australia database; chronic health conditions and risk factors by baseline questionnaire	Diabetes 23,779 / without diabetes 239,703.	CM/CC: number of chronic conditions (0/1/2/≥3, five considered: cancer, heart disease, high blood pressure, stroke, anxiety, and depression).<br>CM: depression, anxiety.<br>CC: heart disease.<br>CMRF: high blood pressure, obese, alcohol consumption	CM/CC: Number of chronic conditions 41.2/32.1/15.7/11.0% and 54.7/28.7/11.7/4.8% (overall p<0.001).<br>CM: depression 17.9% and 14.1 % (p<0.001); anxiety: 11.5% and 9.8% (p<0.001).<br>CC: heart disease 3.3% and 10.6% (p<0.001).<br>CMRF: high blood pressure 45.0% and 22.2%; obese 39.6% and 19.6% (p<0.001); alcohol	n/a	Prevalence % unadjusted; age and sex-adjustment NA.	In diabetes vs. without: greater health burden of in number of chronic conditions, depression, anxiety, heart disease and cardiometabolic risk factors; prediabetes data NA; age-and sex-adjustment NA; investigated count measure of five conditions: three CMs, one CC and one CMRF,

	(self-reported diagnoses).		(0/1-6/ ≥7 drinks/week), smoking status (never/former/current, physical activity (sedentary/insufficient /sufficient).	consumption 46.6/24.3/26.3% and 31.2/29.1/38% (p<0.001); smoking status 49.8/43.0/7.3% and 57.1/35.4/7.5% (p<0.001); physical activity 13.0/21.2/56.6% and 37.1/36.8/19.6% (p<0.001).			and in addition four other CMRFs.
Limongi et al. (2014)[7]	Italian Longitudinal Study on Aging (ILSA), a prospective community-based cohort study in individuals aged 65–84 years; interview, clinical examination, laboratory and diagnostic tests.	T2D 431 / without T2D 2,700.	CC: myocardial infarction, angina, stroke, distal symmetrical neuropathy.<br>CMRF: hypertension, current smoker.	CC: myocardial infarction 12.1% and 7.9%;<br>angina 11.6% and 7.0%;<br>stroke 9.3% and 5.6%;<br>distal symmetrical neuropathy 23.7% and 5.1%.<br>CMRF: hypertension 74.2 % and 63.3%; current smoker 12.5% and 14.8%.	n/a	Prevalence % unadjusted (comparisons were not tested); age- and sex-adjustment NA	In T2D vs without: greater health burden of myocardial infarction, angina, stroke, distal symmetrical neuropathy and CMRFs; prediabetes data NA; age- and sex-adjustment NA; count measure NA; investigated four conditions as CCs and 2 CMRFs.
Greiver et al. (2014)[8]	The Canadian Primary Care Sentinel Surveillance Network (CPCSSN) which included records of patients aged ≥10 years; a large, validated national primary care Electronic Medical Records-based database.	Diabetes 25,425 / without diabetes 247,044.	CM: number of comorbid conditions (7 considered: hypertension, COPD, depression, osteoarthritis, dementia, parkinsonism, epilepsy), depression.	CM: mean number of comorbid conditions 1.06 and 0.49, a difference of 0.57 (95% CI 0.56 to 0.58, p<0.001), prevalence ratio 1.29 (95% CI 1.27 to 1.31, p<0.0001);<br>depression prevalence ratio 1.31 (1.28-1.35), p<0.001).	n/a	mean, prevalence ratio unadjusted; age and sex-adjustment NA.	In diabetes vs. without: greater health burden of number of comorbid conditions and depression; prediabetes data NA; age-and sex-adjustment NA; investigated count measure of seven conditions: six CMs and one CMRF.
Sluik et al (2014)[9]	European Prospective Investigation into Cancer and Nutrition	Diabetes 6,384 / without diabetes 258,911.	CM: cancer.<br>CC: heart disease, stroke.<br>CMRF: hypertension, physical activity	CM: cancer 4% and 3%.<br>CC: heart disease 7% and 1%; stroke 4% and 1%.<br>CMRF: hypertension 55% and 27%; physical activity	n/a	Prevalence % unadjusted (comparisons were not tested); age and sex-adjustment NA.	In diabetes vs. without: greater health burden of heart disease, stroke and hypertension, and a similar health burden of

	(EPIC), an ongoing multicentre prospective study in ten European Countries in individuals aged 35-70 years; questionnaires (self-reported diagnoses).		(low/medium/high/very high), smoking status (never/former/current).	26/23/25/26% and 22/21/23/24%; smoking status 39/28/25% and 42/29/28%.			cancer, physical activity and smoking status; prediabetes data NA; age-and sex-adjustment NA; count measure NA; investigated one CM, two CCs and three CMRFs.
Fano et al. (2013)[10]	All residents in the Local Health Authority 'Roma D' aged ≥35 years; record linkage with hospital discharge registry, ICD-codes.	Total diabetes 27,642 / without diabetes 304,281; Men diabetes 13,948 / without diabetes 137,214; Women diabetes 13,694 / without diabetes 167,067.	CM: all cancers.<br>CC: retinopathy, cardiovascular diseases (≥1, ICD-codes 390-459), ischaemic diseases, myocardial infarction, stroke, renal disease, lower limb amputations.	Men<br>CM: all cancers RR 1.43 (1.34-1.54).<br>CC: retinopathy 3.59 (2.93-4.39);<br>cardiovascular diseases (≥1) 1.81 (1.71-1.91);<br>ischaemic diseases 2.23 (2.04-2.44);<br>myocardial infarction 1.90 (1.65-2.18);<br>stroke 2.63 (2.32-2.98);<br>renal diseases 3.11 (2.69-3.59);<br>lower limb amputations 4.11 (3.51-4.81).<br>Women<br>CM: all cancers 0.96 (0.88-1.04).<br>CC: retinopathy 2.82 (2.28-3.48);<br>cardiovascular diseases (≥1) 1.84 (1.73-1.95);<br>ischaemic diseases 2.96 (2.59-3.38);<br>myocardial infarction 2.62 (2.20-3.13);<br>stroke 2.40 (2.10-2.74);<br>renal diseases 4.04 (3.38-4.82); lower limb amputations 3.85 (3.18-4.66).	n/a	Relative risk (RR) (95% CI) adjusted for gender, age, deprivation index and nationality.	In men and women, in diabetes vs. without (adjusted): greater health burden of retinopathy, cardiovascular diseases, ischaemic diseases, myocardial infarction, stroke, renal diseases and lower limb amputations and greater health burden of cancer only in men; prediabetes data NA; age-and sex-adjustment NA; count measure NA; investigated one CM and seven conditions as CCs.

Du et al. (2013)[11]	German Health Update 2009 in non-institutionalized German adult population aged ≥50 years; telephone interview (self-reported diagnoses).	Diabetes 1,035 / without diabetes 8,098.	CM/CC: mean number of comorbidities. CM: cancer, depression, severe hearing impairment, digestive disorders, chronic liver disease. CC: coronary heart disease (angina pectoris and myocardial infarction), stroke, chronic renal disease. CMRF: hypertension, hyperlipidaemia.	CM//CC: mean number of comorbidities 3.7±2.4 and 2.2±2.0, p <0.001. CM: cancer 16.8% and 11.4%, OR 1.37 (1.07-1.77), OR2 1.43 (1.10-1.85); depression 8.6% and 7.0%, OR1 1.44 (1.06-1.95), OR2 1.24 (0.90-1.70); severe hearing impairment 5.5% and 5.2%, OR1 0.86 (0.57-1.29), OR2 0.72 (0.47-1.11); digestive disorders 7.3% and 0.7%, OR1 1.60 (1.13-2.25), OR2 1.58 (1.12-2.24); chronic liver disease 5.7% and 1.8%, OR1 3.30 (2.10-5.18), OR2 2.72 (1.76-4.19). CC: coronary heart disease 31.0% and 13.0%, OR1 2.52 (2.03-3.13), OR2 2.24 (1.77-2.84); stroke 7.9% and 3.8%, OR1 1.80 (1.25-2.59), OR2 1.69 (1.13-2.54); chronic renal disease 8.3% and 1.8%, OR 1 4.33 (2.79-6.72), OR 3.72 (2.30-6.03). CMRF: hypertension 73.5% and 40.8%, 3.60 (2.95-4.40), OR2 2.57 (2.08-3.18); hyperlipidaemia 52.8% and 32.4%, OR1 2.21 (1.84-2.64), OR2 2.00 (1.65-2.43).	n/a	Mean ±SD unadjusted; OR1: adjusted for age and sex. OR2 : adjusted for age, sex, region of residence, body mass index, smoking status, sports activities and educational attainment	In diabetes vs. without: greater health burden of in mean number of comorbidities, and of cancer, depression, digestive disorders, chronic liver disease, coronary heart disease, stroke, chronic kidney disease, hypertension and hyperlipidaemia, but not from severe hearing impairment; prediabetes data NA; investigated count measure and individual associations of twenty individual chronic conditions of which twelve CMs (also asthma, chronic bronchitis, gastritis/duodenitis, gastric duodenal ulcers, osteoarthritis, rheumatoid arthritis, osteoporosis, chronic back pain), six CCs (also congestive heart failure) and two CMRFs.
Ziegler et al. (2009 & 2008)[12]	MONItoring trends and determinants in Cardiovascular/ Cooperative	Diabetes 195 / IGT 46, IFG 71 / NGM 81; controls without	CC: neuropathic pain (Michigan Neuropathy Screening Instrument (MNSI),	CC: neuropathic pain 13.3% (8.9–18.9) and 1.2% (0.03–6.7);	CC: neuropathic pain IGT 8.7% (2.4–20.0); IFG 4.2% (0.9–11.9); and NGM 1.2% (0.03–6.7);	Prevalence % (95% CI) unadjusted.	In diabetes vs. NGM: greater health burden of neuropathic pain, polyneuropathy, stroke, peripheral artery disease

	Research in the Region of Augsburg (MONICA) study in individuals aged 25-74 years; questionnaires (self-reported diagnoses), clinical examination, laboratory tests.	diabetes were matched for age and sex.	polyneuropathy (MNSI >2), stroke, peripheral artery disease (ankle-brachial index <0.9). CMRF: current smoking, high alcohol consumption, low physical activity.	polyneuropathy 28.0% (21.5-34.5) and 7.4% (2.8-15.4); stroke 10.4% and 5.1%; peripheral artery disease 16.2% and 3.7%. CMRF: current smoking 9.7% and 7.4%; high alcohol consumption 6.7% and 10.0%; low physical activity 20.0% and 45.7%.	polyneuropathy IGT 13.0% (4.9-26.3), IFG 11.3% (5.0-31.0), prediabetes 12.0%, and NGM 7.4% (2.8-15.4); stroke IGT 4.3%, IFG 2.8%, prediabetes 5.1%, and NGM 5.1%; peripheral artery disease IGT 2.2%, IFG 8.5%, prediabetes 6.0%, and NGM 3.7%; CMRF: current smoking IGT 2.2%, IFG 18.3%, prediabetes 12.0%, and NGM 7.4%; high alcohol consumption IGT 8.7%, IFG 26.8%, prediabetes 23.0% and NGM 10.0%; low physical activity IGT 32.6%, IFG 32.4%, prediabetes 32.5%, and NGM 45.7%.		and CMRFs; in prediabetes vs. NGM: greater burden of neuropathic pain, peripheral artery disease and CMRF and these percentages lie between the burden of diabetes and NGM; age- and sex-adjustment NA; count measure NA; investigated four conditions as CCs and three CMRFs.
Varas-Lorenzo et al. (2006)[13]	Third National Health and Nutrition Survey (NHANES III) in adults aged ≥65 years; questionnaires (self-reported diagnoses), clinical examination, laboratory tests.	Diabetes 783 (non-diagnosed diabetes 97) / prediabetes 199 (IGT 159, IFG 40) / NGM 315; multi-ethnic, mainly caucasian.	CC: heart attack, coronary heart disease, stroke, cardiovascular disease (≥1, considered coronary heart disease, stroke, heart failure, peripheral arteriopathy). CMRF: hypertension, obesity, LDL-C ≥160 mg/dl, HDL-C <40mg/dl, triglycerides ≥200 mg/dl, current smokers.	CC: heart attack 22.1% and 7.5%; coronary heart disease 30.8% and 13.1%; T2D OR1 2.27 (1.33-3.87), OR2 2.27 (1.32-3.91); non diagnosed diabetes OR1 1.79 (0.97-3.30), OR2 1.62 (0.87-3.01) stroke 16.9% and 2.0%; cardiovascular disease (≥1): 50.6% and 16.7%. T2D OR1 3.22 (2.02-5.12), OR2 3.24 (2.02-5.20); non diagnosed diabetes OR1 1.96 (1.15-3.37), OR2 1.85 (1.07-3.19). CMRF: hypertension 72.5% and 50.1%; obesity 33.5% and 19.4%; LDL-C ≥160 mg/dl 20.5% and 31.4%; HDL-C <40mg/dl 37.0% and 18.1%, triglycerides ≥200 mg/dl	CC: heart attack 10.6% and 7.5%; coronary heart disease 18.6% and 13.1%; IGT OR1 1.83 (1.12-3.00), OR2 1.88 (1.14-3.09); IFG OR1 1.11 (0.41-3.03), OR2 0.95 (0.34-2.63) stroke 2.5% and 2.0%; cardiovascular disease (≥1) 20.6% and 16.7%; IGT OR1 1.28 (0.80-2.04), OR2 1.30 (0.81-2.08); IFG OR1 1.47 (0.65-3.32), OR2 1.39 (0.60-3.19). CMRF: hypertension 62.3% and 50.1%; obesity 25.6% and 19.4%; LDL-C ≥160 mg/dl 30.2% and 31.4%; HDL-C <40mg/dl 18.1% and 18.1%; triglycerides ≥200 mg/dl 23.6% and 11.7%; current smokers 14.6% and 19.9%.	Prevalence % unadjusted and calculated for groups diabetes, prediabetes and NGM (comparisons were not tested); OR1 (95% CI) crude; OR2 (95% CI) adjusted for age, sex and ethnic group; OR1 and OR2 only available for separate groups.	In diabetes vs. NGM: greater health burden of heart attack, coronary heart disease, cardiovascular disease, and CMRFs; in prediabetes vs. NGM: greater health burden of heart attack and coronary heart disease and CMRFs and these percentages lie in between the burden of diabetes and NGM, but results show no greater burden of stroke and cardiovascular disease; age-and sex-adjustment only available for separate groups; investigated four conditions as CC and six measures as CMRF.

				40.3% and 11.7%, current smokers 9.7% and 19.9%.			
Naess et al. (2003)[14]	Health screening Nord-Trøndelag, Norway in individuals aged ≥20 years; questionnaires (self-reported diagnoses).	HUNT 1 77,224. Young (aged <40 years) diabetes 104 / without diabetes 26,266; middle-aged (aged 40-59 years) diabetes 301 / without 23,823; old (aged ≥60 years) diabetes 1,837 / without 24,893.	CM: hearing impairment, mobility impairment. CC: infarction, angina, stroke.	CM: hearing impairment middle-aged 4.0% and 2.6%, OR 1.25; old 14.4% and 10.7% OR 1.09. mobility impairment middle-aged 9.6% and 4.2%, OR 1.96; old 25.7% and 14.6% OR 1.71. CC: infarction middle-aged 5.6% and 0.9%, OR 4.4; old 12.5% and 6.1%, OR 2.2; angina: middle-aged 7.6% 1.6%, OR 3.5; old 21.7% and 10.6%, OR 2.1; stroke middle-aged 2.7% and 0.5%, OR 4.3; old: 12.0% and 4.2% OR 2.5.	n/a	Prevalence % unadjusted (comparisons were not tested); OR adjusted for age and sex (no confidence intervals available)	In diabetes vs. without: greater health burden of hearing impairment, mobility impairment, infarction, angina and stroke; prediabetes data NA; count measure NA; investigated eight morbidities: five CMs (also vision impairment, other physical disease and psychological distress) and three conditions as CCs.

Abbreviations: BMI, body mass index; CC, classical complications; CES-D, Centre for Epidemiologic Studies Depression Scale; CI, confidence interval; CM, comorbidities; CMRF, cardiometabolic risk factors; IFG, impaired fasting glucose; IGT, impaired glucose tolerance; NA, not available; n/a, not applicable; NGM, normal glucose metabolism; OR, odds ratio; RR, relative risk ratio estimate; T2D, type 2 diabetes; vs, versus.
‡ (mainly) type 2 diabetes versus no diabetes or normal glucose metabolism as specified under study population.

Table S-B. Overview of population-based studies of individual comorbidities in individuals with and without (pre)diabetes

Reference	Data source	Study population, N	Comorbidities specified	Findings in individuals with versus without (mainly) type 2 diabetes‡	Findings in individuals with versus without prediabetes	Adjustments reported	Conclusion paper
McGrath et al. (2021)[15]	Community-dwelling adults in Ireland, England and the USA (The Irish Longitudinal Study on Ageing, the English Longitudinal Study on Ageing, and the Health and Retirement Study) in adults aged ≥ 50 years; questionnaires (self-reported diagnoses).	Diabetes (mainly) type 2 / without diabetes: Ireland 629/7,491; England 942/7,691; USA 4,011 / 14,597.	Any depression (self-reported doctor-diagnosed or current depression symptoms)	Ireland 17.5% (14.3-20.8) and 12.4% (11.5-13.3); Men 14.6% (11.7-17.5) and 9.7% (8.7-10.8); Women 21.7% (17.6-25.7) and 14.8% (13.6-16.1). England 27.2% (24.0-30.3) and 18.6% (17.7-19.5); Men 23.3% (20.2-26.3) and 15.0% (13.8-16.3); Women 32.0% (28.3-35.6) and 21.5% (20.2-26.3). USA 34.1% (32.2-36.0) and 25.1% (24.2-26.0); Men (27.3 (25.3-29.3) and 19.0 (18.7-20.1); Women 40.8% (38.5-43.0) and 30.0% (28.9-31.2).	n/a	Prevalence % (95% CI), age-standardised, stratified by sex.	In diabetes vs. without: greater health burden for any depression; count measure NA; investigated one CM.
Huang et al (2018)[16]	Three population-based cohorts, Nurses' Health Study I and II women aged 30-55 years and 25-42 years respectively and the Health Professionals Follow-up Study men aged 40-75 years; questionnaires; self-reported physical examination.	Nurses' Health study I 4,633 with diabetes / without diabetes 52,502; Nurses' Health Study II 2,205 / 70,408; Health Professionals Follow-up Study 1,118 / 17,605.	Incident obstructive sleep apnoea (self-reported if diagnosed by sleep study).	Incident obstructive sleep apnoea: HR1: 2.14 (1.49 – 3.07); HR2: 1.53 (1.32-1.77); and HR 3 1.08 (1.00-1.16).	n/a	Hazard ratio (HR) (95% CI) 1: stratified by age and calendar time; PR 2 additionally adjusted for race/ethnicity, menopausal status in women, family history of diabetes, duration of postmenopausal hormone use, smoking, alcohol consumption, diet quality, regular physical examination, sleep duration, duration of night-shif work, physical activity, hypertension; PR 3 additionally adjusted for BMI, waist circumference.	In diabetes vs. without: greater health burden for obstructive sleep apnoea; count measure NA; investigated one CM.

Carey et al. (2018)[17]	Population-based cohort study using Clinical Practice Research Datalink data from 361 general practices in England with linked hospitalization and mortality records; electronic medical records (Read code) in individuals aged 40-89 years. Individuals with diabetes were age-sex-practice matched with controls.	Diabetes type 2 96,630 / without diabetes (matched controls) 203,518	Incidence of any infection with a prescription as treatment and any infection-related hospitalization.	Any infection with a prescription as treatment:<br>IRR 1.47 (1.46-1.49);<br>Any infection-related hospitalization:<br>IRR 1.88 (1.83-1.92).	n/a	Incidence rate ratio (IRR) (95% CI) adjusted for age, sex, smoking, body mass index and deprivation.	In diabetes vs without: greater health burden for infection; count measure NA; investigated one CM.
Ballotari et al. (2017)[18]	Population-based cohort of Reggio Emilia with use of the diabetes and cancer registry aged 20-84 December 31st 2009; collected health databases.	Diabetes 23,358 / without diabetes 383,799	Overall cancer incidence	Overall cancer incidence IRR 1.22 (1.15-1.29) driven by liver, pancreas, colon, rectum, bladder in all sexes and corpus uteri for females.	n/a	Incidence rate ratio (IRR) (95% CI) adjusted for age, sex and foreign status.	In diabetes vs without: greater health burden for overall cancer; count measure NA; investigated one CM.
Shinkov et al (2018)[19]	Survey of most common endocrine disorders in Bulgaria in individuals aged 20-94 years; questionnaires (self-reported diagnoses), clinical examination, laboratory tests.	Diabetes 166 (new 70, known 96) / prediabetes 221 (IGT 43 / IFG 178) / NGM 1,681.	Depression (self-rating depression scale).<br>Anxiety (self-rating anxiety scale).	Men<br>Depression: 26.1% and 15.0%.<br>Anxiety: 35.2% and 21.0%.<br>Women<br>Depression: 64.1% and 32.3%.<br>Anxiety: 71.8% and 54.0%.	Men<br>Depression: 20.0% and 15.0%.<br>Anxiety: 30.8% and 21.0%.<br>Women<br>Depression: 42.9% and 32.3%<br>Anxiety: 64.8% and 54.0%.	% unadjusted; age- and sex-adjustment NA.	In diabetes and prediabetes vs. without: greater health burden for anxiety and depression; age- and sex-adjustment NA; count measure NA; investigated two CMs.

Hamilton et al. (2017)[20]	Community-based Fremantle Diabetes Study Phase I (Australia) in residents with diabetes (mean age 64.0 years) which were matched with four age-, sex-, and zip code-matched residents without diabetes; linkage with hospital records, ICD-codes.	With diabetes 1,291 / matched residents without diabetes 5,159.	First and all hip fracture.	First and all hip fracture: 1.33 (1.05 — 1.68) and 1.28 (1.02 — 1.59).<br>First incident hip fracture: cause-specific hazard ratio 1.50 (1.19 -1.89).	n/a	Incidence rate (95% CI) unadjusted; hazard ratio (95% CI) adjusted for baseline age, sex and charlson comorbidity index.	In diabetes vs. without: greater health burden for hip fracture; prediabetes data NA; only age- and sex-adjustment NA; count measure NA; investigated one CM.
Deschenes et al. (2016)[21]	Community-based Emotional Well-Being, Metabolic Factors and Health Status (EMHS) study in adults aged 40-69 years without diabetes; questionnaires (self-reported diagnoses) and clinical examination.	Prediabetes 1,058 / no (pre)diabetes 1,428.	High anxiety symptoms (GAD-7). High depressive symptoms (PHQ-9).	n/a	High anxiety: 10.2% and 11.3%.<br>High depression: 14.0% and 12.4%.	Prevalence % unadjusted (comparisons were not tested); age and sex-adjustment NA.	In prediabetes vs. NGM: a similar health burden of anxiety and depression; diabetes data NA; age- and sex-adjustment NA; . count measure NA; investigated two CMs.
Fleiner et al. (2016)[22]	Health screening Nord- Trøndelag, Norway in individuals aged ≥20 years; questionnaires.	HUNT 2 Men T2D 656 / without diabetes 10,192. Women T2D 753 / without diabetes 22,281,	Hypothyroidism and hyperthyroidism (based on TSH level, use of levothyroxine or thionamides and/or self-reported).	Men<br>Hypothyroidism: any 6.3% (4.6-8.4) and 3.1% (2.7-3.5); PR1 1.19 (0.87-1.63); PR2 1.04 (0.75-1.44); treated 1.8 % (1.0-3.2) and 0.9% (0.7-1.1); PR1 1.14 (0.63-2.08); PR2 1.00 (0.53-1.86).<br>Hyperthyroidism: any 3.4% (2.2-5.0) and 1.7% (1.3-2.2); PR1 1.75 (1.12-2.73); PR2 1.95 (1.23-3.10); diagnosed 0.8% (0.3-1.8) and 0.4%	n/a	Prevalence % unadjusted; prevalence ratio (PR1) (95% CI) adjusted for age; PR2 adjusted for age, smoking and BMI.	In men with diabetes vs. without: greater health burden of hyperthyroidism, but not hypothyroidism; in women with diabetes vs. without: greater health burden of treated hypothyroidism but not any hypothyroidism or hyperthyroidism; prediabetes data NA; count measure NA; investigated one CM.

				(0.3-0.5); PR1 1.08(0.42-2.786); PR2 1.01(0.39-2.63).<br>Women<br>Hypothyroidism: any 12.7% (10.6-15.3) and 8.3% (7.7-8.9); PR1 1.00 (0.82-1.21); PR2 0.89 (0.73-1.09); treated 9.6% (7.7-11.9) and 4.6%(4.3-5.0); PR1 1.26 (1.00-1.58); PR2 1.16 (0.92-1.46).<br>Hyperthyroidism: any 6.6% (5.1-8.7) and 4.7% (4.1-5.3); PR1 1.15 (0.87-1.52); PR2 1.21 (0.89-1.63); diagnosed 3.5% (2.4-5.0) and 2.3% (2.0-2.7); PR1 0.99 (0.67-1.47); PR2 0.93 (0.61-1.41).			
Berge et al. (2015)[23]	Cross-sectional sample of the Hordaland Health Study (HUSK) in middle-aged (40-47 years) or older adults (70-72 years); questionnaires and clinical examination.	Total diabetes 353 (of which insulin treated 49 / orally treated 129 / un-medicated 175) / without diabetes 21,492.	Depression (reported HADS-d ≥8 and/or use of antidepressant agents).	Total<br>Depression HADS-d ≥8: 14.1% and 9.6%.<br>Depression HADS-d ≥ 8 and/or use of antidepressant agents: 19.5% and 11.8%.<br>Overall depression (HADS-d ≥ 8 and/or use of antidepressant agents): OR1 1.69 (1.28-2.22).<br>40-47 years<br>Depression HaDS-d≥8: OR1 1.8 (1.3-2.8).<br>70-72 years<br>Depression HaDS-d≥8: no significant OR (not reported).	n/a	Prevalence % unadjusted (comparison was not tested in total group); OR1 (95% CI) adjusted for age and sex as appropriate; differences between age groups were not significant.	In diabetes vs. without: greater health burden for depression; prediabetes data NA; count measure NA; investigated one CM.
Peeters et al. (2015)[24]	Population-based cohort study within the Clinical Practice Research Datalink of which all patients aged ≥18 years with diabetes and 1 reference matched by birth year, sex,	T2D 300,039 / without diabetes 300,039.	Colorectal cancer.	Colorectal cancer: HR1 1.32 (1.25-1.40); HR2 1.26 (1.18-1.33).	n/a	Hazard ratio (HR) 1 (95% CI) adjusted for age and sex; HR2 additionally adjusted statin use in the previous 6 months, smoking and alcohol consumption.	In diabetes vs. without: greater health burden for colorectal cancer; prediabetes data NA; count measure NA; investigated one CM.

	practice were selected; electronic medical records.						
Zhao et al. (2014)[25]	2005-2008 National health and Nutrition Examination Survey (NHANES) in individuals aged ≥40 years; home interview with questionnaires (self-reported diagnose), clinical examination, laboratory tests.	Diabetes 517 / prediabetes 1483 / NGM 1,026; multi-ethnic, mainly caucasian.	Glaucoma.	Glaucoma: 9.5% and 2.6%; OR1: 2.28 (1.37-3.81); OR2: 2.12 (1.23, 3.67).	Glaucoma: 2.5% and 2.6%; OR1: 1.02 (0.63-1.66); OR2: 1.01 (0.57, 1.82)	Prevalence % unadjusted; odds ratio (OR) 1 (95% CI) adjusted for age, sex, ethnicity; OR2 additional adjusted for smoking, physical activity, alcohol intake, education, and BMI.	In diabetes vs. NGM: greater health burden of glaucoma; in prediabetes vs. NGM: comparable health burden of glaucoma; count measure NA; investigated one CM.
Wandell et al. (2014)[26]	All living individuals who resided in Stockholm County, Sweden, on January 1, 2011 (all ages); registry data (electronic medical records) primary and specialist outpatient care, ICD-codes	Total diabetes 96,103 / without diabetes 1,962,305 Men diabetes 54,439 / without diabetes 963,676. Women diabetes 41,664 / without diabetes 998,629.	Depression. Anxiety disorder.	Men<br>Depression:<br>all ages 6.32% and 3.65%;<br>age 35-65 7.58% and 4.57%;<br>age 65-74 4.69% and 3.29%;<br>OR 1.5 (1.5-1.6).<br>Anxiety disorder:<br>all ages 4.04% and 3.35%;<br>age 35-65 5.24% and 3.72%;<br>age 65-75 2.51% and 2.30%;<br>OR 1.4 (1.3-1.4).<br>Women<br>Depression:<br>all ages 10.52% and 7.21%;<br>age 35-65 13.88% and 9.07%; age 65-74 8.14% and 6.61%;<br>OR 1.4 (1.4-1.5)<br>Anxiety disorder:<br>all ages 7.36% and 6.41%;<br>age 35-65 9.50% and 7.01%;<br>age 65-75 5.57% and 4.86%;<br>OR 1.3 (1.2-1.3).	n/a	Prevalence % unadjusted (comparisons were not tested); odds ratio (OR) (95% CI) adjusted for age.	In men and women with diabetes vs. without: greater burden of depression and anxiety; prediabetes data NA; count measure NA; investigated two CMs. .
Icks et al. (2013)[27]	Population-based Heinz Nixdorf Recall study in randomly selected	Diabetes 449 (of which 194 undetected)	Incident high depressive symptoms (CES-D).	High depressive symptoms: Cumulative 5-year incidences diagnosed, undetected, and without	n/a	Cumulative 5-year incidence (95% CI) unadjusted; OR1 adjusted for age,	In diagnosed and undiagnosed diabetes vs. without: not greater health burden of

	men and women aged 45–75 years; self-administered questionnaires, face-to-face interviews; laboratory tests, clinical examination.	/ without diabetes 3,184.		diabetes were 7.1 (4.2-10.9), 4.1 (1.8-8.0), and 6.5 (5.6-7.4), respectively. Diagnosed diabetes OR1 1.22 (0.74-2.03); OR2 1.00 (0.59-1.68); undiagnosed diabetes OR1 0.72 (0.35–1.48); OR2 0.62 (0.30–1.30).		sex; OR2 additionally adjusted for BMI, myocardial infarction, stroke, no regular physical activity, education.	depressive symptoms; prediabetes data NA; count measure NA; investigated one CM.
Redaniel et al. (2012)[28]	Cohort study within the UK General Practice Research Database in women aged ≥35 years in which diabetes was diagnosed between 1987 and 2007 and women without diabetes were randomly selected; registry data.	T2D 52,657 / without diabetes 30,210.	Breast cancer.	Breast cancer: HR1 1.29 (1.16–1.44); HR2 1.25 (1.12-1.40); HR3 1.12 (0.98–1.29).	n/a	HR1 (95% CI) unadjusted; HR2 adjusted for age, period of cohort entry, region; HR3: additionally adjusted for BMI.	In T2D vs. without T2D: greater health burden of breast cancer; prediabetes data NA; count measure NA; investigated one CM.
Kengne et al. (2012)[29]	Seven population-based cohorts in the United Kingdom comprising both Scottish Health Surveys (2) and the Health Surveys for England (5) (mean age 54.4-68.8); interviews, clinical examination, laboratory tests.	Diabetes 946 / without diabetes 25,534.	Anaemia (haemoglobin concentrations <13 g/dl (men) and <12 g/dl (women)).	Anaemia: 14.3% and 8.2% (p<0.001).	n/a	Prevalence % unadjusted (comparisons were not tested); age- and sex-adjustment NA.	In diabetes vs. without: greater health burden of anaemia; prediabetes data NA; age- and sex-adjustment NA; count measure NA; investigated one CM.
Plantinga et al. (2012)[30]	2005-2008 National health and Nutrition Examination Survey (NHANES) in	Diabetes 1,424 / without diabetes 8,424; multi-ethnic,	Sleep apnoea. Depression (PHQ-9).	Sleep apnoea: OR 1.45 (1.06-1.98). Depression: 7.2 % (5.4-9.4) and 4.9% (4.2-5.7) (p=0.02).	n/a	Depression prevalence % unadjusted; age- and sex-adjustment NA.	In diabetes vs. without: greater health burden of sleep apnoea and depression; prediabetes data NA; age- and sex-adjustment NA; count

	individuals aged ≥20 years; questionnaires (self-reported diagnoses), clinical examination, laboratory tests, home interview.	mainly caucasia.				Sleep apnoea only age and sex adjustment NA; OR adjusted for demographics, BMI, cardiovascular and kidney disease, alcohol use.	measure NA; investigated two CMs.
Bouwman et al. (2010)[31]	The New Hoorn Study in which individuals aged 40-65 years were included; questionnaires, clinical examination, laboratory tests.	T2D 181 / prediabetes (IGM) 425 / NGM 2,061.	Depression (CES-D, score ≥16). Anxiety (HADS-A, score ≥8).	Total<br>Depression: 21.0% and 12.5%. OR1 2.02 (1.35-3.04); OR2 1.77 (1.13-2.78).<br>Anxiety: 19.9% and: 15.0% (n-s).<br>Men<br>Depression: 19.6% and 7.7%, OR1 3.16 (1.78-5.62), OR2 2.55 (1.34-4.86).<br>Anxiety: 16.5% and 10.2%, OR1 1.91 (1.06-3.47), OR2 1.58 (0.82-3.06).<br>Women<br>Depression: 22.6% and 16.4%, OR1 1.39 (0.78-2.49), OR2 1.37(0.71-2.62).<br>Anxiety: 23.8% and 18.9%, OR1 1.33 (0.76-2.31), OR2 1.36 (0.74-2.51).	Total<br>Depression: 12.2% and 12.5%; OR1 1.13 (0.81-1.57); OR2 1.09 (0.78–1.54).<br>Anxiety 15.3% and 15.0% (n-s).<br>Men<br>Depression: 9.5% and 7.7%; OR1 1.26 (0.76-2.09); OR2 1.14 (0.67-1.94).<br>Anxiety: 11.6% and 10.2%; OR1 1.11 (0.70-1.78); OR2 1.07 (0.65-1.76).<br>Women<br>Depression: 15.8% and 16.4%; OR 1.07 (0.69-1.66); OR2 1.05 (0.67-1.67).<br>Anxiety: 20.1% and 18.9%; OR1 1.16 (0.78-1.74); OR2 1.14 (0.74-1.75).	Prevalence % unadjusted; OR1 (95% CI) adjusted for education, sex, age, family history of T2D; OR2 additionally adjusted for triglycerides, HDL cholesterol, total cholesterol, hypertension, smoking, waist circumference.	In T2D, but not prediabetes, vs. NGM: greater health burden of depression, and, in men only greater burden of anxiety; only age- and sex adjustment NA; count measure NA; investigated two CMs.
Gagnon et al. (2010)[32]	Austrialian, Diabetes, Obesity and Lifestyle Study (AusDiab) in individuals aged ≥40 years; questionnaires (self-reported diagnoses), clinical	Prediabetes 1,400 / NGM 4,855.	Fractures.	n/a	Men<br>no significant results (not reported).<br>Women<br>Low trauma fracture: 0.75 (0.53-1.05).<br>All incident fractures: 0.70 (0.52-0.95).	Odds ratio (OR) (95% CI) adjusted for age and BMI.	In women with prediabetes vs. NGM, but not in men, there was a lower health burden of fractures; diabetes data NA; count measure NA; investigated one CM.

	examination, laboratory tests.						
Mitchell et al. (2009)[33]	The Blue Mountains Hearing Study in individuals aged ≥49 years; interviews, questionnaire, clinical examination, laboratory tests, audiological examination.	T2D 210 / without T2D 1,648.	Hearing loss ($PTA_{0.5–4kHz}$ > 25 dB HL in the better ear).	Hearing loss: 50% vs. 38.2%; OR1 1.64 (1.19-2.26); OR2 1.55 (1.11-2.17).	n/a	Prevalence % unadjusted (comparisons were not tested); OR1 (95% CI) adjusted for age, sex; OR2 additionally adjusted for low educational attainment, smoking, work in noisy industry.	In T2D vs. without T2D: greater health burden of hearing loss; prediabetes data NA; count data NA; investigated one CM.
Lin et al. (2008)[34]	Dutch data of worldwide survey in household-residing adults (mean age 45.0); World Mental Health–Composite International Diagnostic Interview.	Diabetes 68 / without diabetes 1,026.	Major depression. Generalized anxiety disorder. Agoraphobia or panic disorder.	Major depression: 3.3% and 5.4%, OR 0.9 (0.3-2.5); Generalized anxiety disorder: 0.3% and 1.1%, OR 0.3 (0.1-2.3); Agoraphobia or panic disorder: 2.4% and 1.6%, OR 2.1 (0.5-9.7).	n/a	Prevalence % unadjusted (comparisons were not tested); OR (95% CI) adjusted for age and sex.	In diabetes vs. without: a comparable health burden of major depression; prediabetes data NA; count measure NA; investigated two CM.
Li et al. (2008)[35]	2006 Behavioral Risk Factor Surveillance System survey data in individuals aged ≥18; standardized telephone survey of key behavioral risk factors and chronic conditions (self-ported diagnoses).	Diabetes 20,142 / without diabetes 181,433; multi-ethnic, mainly caucasian.	Lifetime anxiety.	Total<br>Lifetime anxiety:<br>19.5% and 10.9%; PR1 1.39 (1.29-1.50); PR2 1.20 (1.12-1.30).<br>Men<br>Lifetime anxiety: PR1 1.37 (1.19-1.56); PR2 1.24 (1.08-1.41).<br>Women<br>Lifetime anxiety: PR1 1.44 (1.32-1.58); PR2 1.19 (1.08-1.30).	n/a	Prevalence ratio 1 % age-adjusted; (PR1) (95% CI) unadjusted; PR2 additionally adjusted for gender, race/ethnicity, age, educational levels, marital status, employment status, current smoking, leisure-time physical activity, and body mass index.	In diabetes vs. without: greater health burden of anxiety disorder; prediabetes data NA; only age- and sex-adjustment NA; only age- and sex-adjustment NA; count measure NA; investigated one CM.
Adriaanse et al. (2008)[36]	The Hoorn Study in individuals aged 50-75 years; questionnaires (self-reported	Men<br>T2D 60 / IGM 86 / NGM 130;	Depression (CES-D score ≥16). Ischaemic heart disease.	Men<br>Depression: 15.0% and 7.7%; OR1 2.04 (0.75-5.49); OR2 1.95 (0.71-5.34).	Men<br>Depression: 7.0% and 7.7%; OR1 0.90 (0.32-2.57); OR2 0.90 (0.31-2.58).	Prevalence % unadjusted (comparisons were not tested); OR1 (95% CI) adjusted	In (pre)diabetes vs. without: greater health burden of depression in women, but not in men; In men and women with

	diagnoses), clinical examination, laboratory tests.	Women T2D 66 / IGM 78 / NGM 130.		Ischaemic heart disease: 38.2% and 31.8%. Women Depression: 19.7% and 7.7%; OR1 3.18 (1.31-7.74); OR2 3.18 (1.26-8.02). Ischaemic heart disease 50.0% and 33.1%.	Ischaemic heart disease: 32.9% and 31.8%. Women Depression: 23.1% and 7.7%; OR1 3.60 (1.57-8.28); OR2 3.04 (1.28-7.21). Ischaemic heart disease 39.7% and 33.1%.	for age, low education; OR2 additionally adjusted for cardiovascular risk factors (triglycerides, HDL cholesterol, total cholesterol, waist circumference, hypertension and smoking). (comparable additional data available with adjustment for cardiovascular disease, and diabetes symptoms).	diabetes and women with prediabetes, but not men, vs. NGM: greater health burden of ischaemic heart disease; only sex- and age adjustment NA; count measure NA; investigated 1 CM and 1 CC.
Kornum et al. (2008)[37]	Population-based case-control study in Denmark in individuals aged ≥15 years which for each case 10 sex- and age-matched population control subjects were selected from Civil Registration System; ICD-codes.	T2D 32,687 / without diabetes 343,654.	Pneumonia.	Pneumonia: OR1 1.65 (1.59-1.71); OR2 1.23 (1.19-1.28).	n/a	Odds ratio 1 (OR1) (95% CI) unadjusted; OR2 level of comorbidity (Charlson index), history of alcoholism-related conditions, preadmission use of antibiotics or immunosuppressants, marital status, household presence of small children attending day care centers, and degree of urbanization	In T2D vs. without: greater health burden of pneumonia; prediabetes data NA; only age- and sex-adjustment NA; count measure NA; investigated one CM.

Abbreviations: BMI, body mass index; CC, classical complications; CES-D, Centre for Epidemiologic Studies Depression Scale; CI, confidence interval; CM, comorbidities; CMRF, cardiometabolic risk factors; dB HL, decibel hearing level; GAD, generalized anxiety disorder; HADS-A, Hospital Anxiety and Depression Scale—Anxiety Subscale; HR, hazard ratio; IFG, impaired fasting glucose; IGT, impaired glucose tolerance; NA, not available; n/a, not applicable; NGM, normal glucose metabolism; OR, odds ratio; PHQ, patient health questionnaire; PR, prevalence ratio; PTA, pure tone average; T2D, type 2 diabetes; vs, versus.
‡ (mainly) type 2 diabetes versus no diabetes or normal glucose metabolism as specified under study population.

Table S-C. Overview of population-based studies of individual classical complications in individuals with and without (pre)diabetes

Reference	Data source	Study population, N	Classical complications specified	Findings in individuals with versus without (mainly) type 2 diabetes‡	Findings in individuals with versus without prediabetes	Adjustments reported	Conclusion paper
Hicks et al. (2021)[38]	Two population-based studies National health and Nutrition Examination Survey (NHANES) aged 40-85 years and Atherosclerosis Risk in Communities (ARIC) in individuals aged 70-89 years; questionnaires and monofilament testing.	Diabetes >10 years/ diabetes <10 years/prediabetes/no diabetes: Aged 40-69 years (NHANES) 110/224/486/2,758. Aged ≥ 70 years NHANES 127/143/390/952; ARIC 295/773/833/1,459.	Peripheral neuropathy	Diabetes >10 years/ diabetes <10 years Aged 40-69 years (NHANES): 5.26 (3.27-8.46)/ 1.82 (1.07-3.10). Aged ≥ 70 years NHANES 2.17 (1.33-3.54)/ 1.50 (1.00-2.27); ARIC 1.68 (1.29-2.20)/ 1.32 (1.09-1.60).	Aged 40-69 years (NHANES): 1.53 (1.01-2.30). Aged ≥ 70 years NHANES 0.89 (0.64-1.22); ARIC 1.19 (0.99-1.44).	Odds ratio (OR) (95% CI) age, sex- and race-adjusted.	In diabetes versus without: greater health burden of peripheral neuropathy. In prediabetes versus without: only in age group 40-69 years greater health burden of peripheral neuropathy; count measure NA; investigated one CC.
Boulanger et al. (2017)[39]	Retrospective population-based cohort study of individuals aged 40-89 years in Scotland.	T2D 189,769 / without diabetes 2,427,087	Incident intracerebral haemorrhage	Intracebral haemorrhage IRR 1.06 (0.99-1.12)	n/a	Incidence rate ratio (IRR) (95% CI) adjusted for age, sex, social economic status.	In T2D vs without: greater but not significant health burden of intracerebral haemorrhage.
Wright et al. (2017)[407]	Population-based cohort study using Clinical Practice Research Datalink data from 383 general practices in England with linked hospitalization and mortality records; electronic medical records (Read code) in individuals aged ≥30 years.	White population incident T2D 143,724 / without T2D (matched controls by year of birth, sex, general practice, index date of diabetes diagnosis)	Cardiovascular disease (≥1 item in Read code). Renal disease (CKD stage ≥4; defined by Read code).	Cardiovascular disease: 22.4% and 8.7%. Renal disease: 1.4% and 0.3%.	n/a	Prevalence % unadjusted (comparisons were not tested); age- and sex-adjustment NA.	In diabetes vs. without: greater health burden of cardiovascular disease and renal disease; prediabetes data NA; age- and sex-adjustment NA; count measure NA; investigated one CC and ≥1 cardiovascular disease item as CC.

		653,390; multi-ethnic (76.5% and 72.0% white ethnicity).					
Melsom et al. (2016)[41]	Prospective cohort study based on the Renal Iohexol Clearance Survey in Tromsø 6 (RENIS-T6) and the RENIS Follow-Up Study in individuals aged 50-62 years; questionnaires, clinical examination, laboratory tests.	Without diabetes 1,261 of which 169 prediabetes.	Glomerular hyperfiltration (defined as GFR > age-, sex-, weight-, height-adjusted 90th percentile).<br>High-normal urinary albumin-creatinin ratio (ACR, >10 mg/g).<br>Chronic kidney disease (CKD, defined as GFR <60 ml/min/1.73m^2).	n/a	Glomerular hyperfiltration:<br>OR1 2.03 (1.27-3.25);<br>OR2 1.95 (1.20-3.17);<br>OR3 1.79 (1.03-3.12).<br>High-normal ACR:<br>OR1 1.92 (1.11-3.32),<br>OR2 1.83 (1.04-3.22),<br>OR3 1.71 (0.93-3.16).<br>CKD:<br>OR M1 0.39 (0.09-1.69),<br>OR M2 0.50 (0.11-2.27).<br>OR M3 0.53 (0.09-3.22).	Odds ratio 1 (OR1) (95% CI) adjusted for baseline age, sex, and use of angiotensin-converting enzyme inhibitor or angiotensin receptor blocker; OR2 additionally adjusted for baseline BMI, daytime systolic ambulatory blood pressure, BMI, smoking, fasting insulin, physical exercise, and in case of CKD for ACR; OR3: additionally adjusted for hyperfiltration status or GFR at baseline OR ACR at baseline as appropriate, change in fasting glucose, and use of antihypertensive medication including angiotensinconverting enzyme inhibitor or angiotensin receptor blocker from baseline to follow-up.	In prediabetes vs. without diabetes: greater health burden of albuminuria and glomerular hyperfiltration; diabetes data NA; only age- and sex-adjustment NA; no count measure; investigated one CC.
Wyld et al. (2015)[42]	National longitudinal population-based	Diabetes 5,587 /	Chronic kidney disease (presence of	Chronic kidney disease: 27.4% and 9.0%.	n/a	Prevalence % unadjusted	In diabetes vs. without: greater

	Austrialian, Diabetes, Obesity and Lifestyle Study (AusDiab) study, in non-institutionalised Australian adults aged ≥25 years; questionnaires; clinical examination; laboratory tests.	without diabetes 551.	eGFR <60 mL/min/1.73 m^2 and/or microalbuminuria).			(comparisons were not tested); age- and sex-adjustment NA.	health burden of chronic kidney disease; prediabetes data NA; age- and sex-adjustment NA; count measure NA; investigated one CC.
Hunger et al. (2014)[43]	KORA S4/F4 cohort study in individuals aged 55-74 years; questionnaires (self-reported diagnoses), clinical examination, laboratory tests.	Previously known diabetes 71 / previously unknown diabetes 80 / prediabetes 442 / NGM 453.	Myocardial infarction. Angina. Stroke.	Myocardial infarction: 6.0% and 4.2%. Angina: 11.3% and 7.8%. Stroke: 2.6% and 2.0%.	Myocardial infarction: 3.2% and 4.2%. Angina: 9.1% and 7.8%. Stroke 2.0% and 2.0%.	Prevalence % unadjusted (comparisons were not tested); age- and sex-adjustment NA.	In diabetes vs. without: greater health burden of myocardial infarction, angina and stroke, but for prediabetes only for angina; age- and sex-adjustment NA; count measure NA; investigated two CCs.
Hernandez et al. (2013)[44]	Multidisciplinary Intervention in Primary Care study in a random sample of the adult Mediterranean population aged 18-80 years; clinical interview (self-reported diagnoses), clinical examination and laboratory tests.	Diabetes 201 / without diabetes 2,069.	Chronic kidney disease. Cardiovascular disease (≥1 of the following: previous coronary heart disease, heart failure, cerebrovascular disease, aortic aneurysm or peripheral arterial disease).	Chronic kidney disease: 19.4% and 5.2%. Cardiovascular disease (≥1 item): 15.4% and 4%.	n/a	Prevalence % unadjusted (comparisons were not tested); age- and sex-adjustment NA.	In diabetes vs. without: greater health burden of chronic kidney disease and cardiovascular disease; age- and sex-adjustment NA; count measure NA; investigated one CC and ≥1 cardiovascular disease item as CC.
Bongaerts et al. (2012)[45]	Cooperative Health Research in the Region of Augsburg (KORA) F4 Survey (2006–2008) non-institutionalized individuals aged 61-82-years; questionnaires,	Diabetes 239 (of which 62 undiagnosed) / prediabetes 284 (of which 46 IFG-IGT, 183 i-IGT and 55 i-IFG) / NGM 577.	Distal sensorimotor polyneuropathy (DSPN, the presence of an impaired bilateral foot-vibration perception and/or an impaired bilateral foot pressure sensation).	DSPN: diabetes all 20.5%, OR 1.54 (1.01-2.87); known diabetes 22.0% (16.2-28.9), OR 1.77 (1.20-2.87); undiagnosed diabetes 16.1% (8.0-27.7); OR 1.22 (0.57-2.61); NGT 11.1% (8.6-13.9).	DSPN: prediabetes all 14.4%; 1.22 (0.78-1.90). IFG-IGT 23.9% (12.6-38.8), OR 2.82 (1.29-6.10); i-IGT 14.8% (10.0-20.7), i-IGT 1.26 (0.76-2.08); i-IFG 5.5% (1.1-15.1), 0.33 (0.10-1.33);	Prevalence % (calculated 95% CI) unadjusted; OR adjusted for age, sex, height, waist circumference, diastolic blood pressure, level of physical activity,	In diabetes vs. NGM: greater health burden of DSPN; in prediabetes vs. NGM: greater health burden of DSPN, but not always significant; only age- and sex-

	clinical examination, laboratory tests.				NGT 11.1% (8.6-13.9).	and alcohol consumption.	adjustment NA; count measure NA; investigated one CC.
Sundling et al. (2012)[46]	Sample from population-based screening survey for hyperglycaemia (second Nord-Trondelag Health study (HUNT2)) in individuals aged ≥20 years who were invited for follow-up eye examination; questionnaires, clinical examination; laboratory tests.	Diabetes 56 (of which 36 newly diagnosed) / IGT 38 / NGM 32.	Diabetic retinopathy (fundoscopy).	Diabetic retinopathy: diabetes all 6.5%, known diabetes 11.1%, undiagnosed diabetes 3.6%, NGM 10.3%.	Diabetic retinopathy: 2.9% and 10.3%	Prevalence % unadjusted (comparisons were not tested); age- and sex-adjustment NA.	In known diabetes vs NGM: similar health burden of diabetic retinopathy; in prediabetes prevalence of diabetic retinopathy was low; age- and sex-adjustment NA; count measure NA; investigated one CC.
Kurcharska -Newton et al. (2010)[47]	Atherosclerosis Risk in Communities in individuals aged 45-64 years; questionnaires (self-reported diagnoses), clinical examination and laboratory tests; baseline and follow-up.	Diabetes 1,550 (43.5% men) / without diabetes 12,428 (42.7% men); multi-ethnic (white and black).	Non-fatal myocardial infarction (at follow-up)	Total<br>Non-fatal myocardial infarction in whites: HR1 2.28 (1.91-2.73); HR2 2.11 (1.76-2.53); HR3 1.60 (1.32-1.94).<br>Men (all race)<br>Non-fatal myocardial infarction:<br>HR1 1.83 (1.51-2.23);<br>HR2 1.83 (1.51-2.23);<br>HR3 1.48 (1.21-1.82).<br>Women (all race)<br>Non-fatal myocardial infarction:<br>HR1 2.91 (2.31-3.68);<br>HR2 2.97 (2.34-3.76);<br>HR3 1.93 (1.49-2.49).	n/a	Cox proportional hazard ratio (HR1) (95% CI) adjusted for age; HR2 additionally adjusted for sex, race/ARIC centre, smoking status and cigarette years of smoking; HR3 additionally adjusted for systolic blood pressure, antihypertensive medication use, gender-by-systolic blood pressure, HDL-cholesterol; data did not differ with race.	In diabetes vs. without: greater health burden of myocardial infarction; prediabetes data NA; count measure NA; investigated one CC.
Rogers et al. (2008)[48]	National longitudinal population-based Austrialian, Diabetes, Obesity and Lifestyle Study (AusDiab) study, in non-institutionalized	Diabetes 250 (7 with type 1 diabetes) / IGT or IFG 455 / NGM 201.	Incident diabetic retinopathy (fundoscopy).	Diabetic retinopathy incidence: 8.0% and 2.0%.	Diabetic retinopathy incidence: 0.7% and 2.0%.	Incidence % unadjusted (comparisons were not tested); age- and sex-adjustment NA.	In diabetes, but not in prediabetes, vs. without: greater health burden of diabetic retinopathy; age- and sex-adjustment

Australian adults aged ≥25 years; questionnaires; clinical examination; laboratory tests; 5-year follow-up.	NA; count measure<br>NA; investigated one CC.

Abbreviations: ACR, albumin-creatinin ratio; BMI, body mass index; CC, classical complications; CI, confidence interval; CKD, chronic kidney disease; CM, comorbidities; CMRF, cardiometabolic risk factors; DSPN, distal sensorimotor polyneuropathy; HR, hazard ratio; i-, isolated; IFG, impaired fasting glucose; IGT, impaired glucose tolerance; NA, not available; n/a, not applicable; NGM, normal glucose metabolism; OR, odds ratio; T2D, type 2 diabetes; vs, versus.
‡ (mainly) type 2 diabetes versus no diabetes or normal glucose metabolism as specified under study population.

References

1. Zghebi SS, Steinke DT, Rutter MK, Ashcroft DM. Eleven-year multimorbidity burden among 637 255 people with and without type 2 diabetes: a population-based study using primary care and linked hospitalisation data. BMJ Open. 2020;10(7):e033866. doi:10.1136/bmjopen-2019-033866.
2. Iranfar N, Smith TC. When Should "Pre" Carry as Much Weight in the Diabetes Comorbidity Debate? Insights From a Population-Based Survey. *Prev Chronic Dis* 2018; **15**: E36.
3. Castro-Rodriguez M, Carnicero JA, Garcia-Garcia FJ, et al. Frailty as a Major Factor in the Increased Risk of Death and Disability in Older People With Diabetes. *J Am Med Dir Assoc* 2016; **17**(10): 949-55.
4. Ervasti J, Virtanen M, Lallukka T, et al. Contribution of comorbid conditions to the association between diabetes and disability pensions: a population-based nationwide cohort study. *Scand J Work Environ Health* 2016; **42**(3): 209-16.
5. Graham E, Gariepy G, Burns RJ, Schmitz N. Demographic, lifestyle, and health characteristics of older adults with prediabetes in England. *Prev Med* 2015; **77**: 74-9.
6. Comino EJ, Harris MF, Islam MD, et al. Impact of diabetes on hospital admission and length of stay among a general population aged 45 year or more: a record linkage study. *BMC Health Serv Res* 2015; **15**: 12.
7. Limongi F, Noale M, Crepaldi G, Maggi S, Group IW. Prevalence of diabetes and depressive symptomatology and their effect on mortality risk in elderly Italians: The Italian Longitudinal Study on Aging. *Diabetes Metab* 2014; **40**(5): 373-8.
8. Greiver M, Williamson T, Barber D, et al. Prevalence and epidemiology of diabetes in Canadian primary care practices: a report from the Canadian Primary Care Sentinel Surveillance Network. *Can J Diabetes* 2014; **38**(3): 179-85.
9. Sluik D, Boeing H, Li K, et al. Lifestyle factors and mortality risk in individuals with diabetes mellitus: are the associations different from those in individuals without diabetes? *Diabetologia* 2014; **57**(1): 63-72.
10. Fano V, Pezzotti P, Gnavi R, et al. The role of socio-economic factors on prevalence and health outcomes of persons with diabetes in Rome, Italy. *Eur J Public Health* 2013; **23**(6): 991-7.
11. Du Y, Heidemann C, Gosswald A, Schmich P, Scheidt-Nave C. Prevalence and comorbidity of diabetes mellitus among non-institutionalized older adults in Germany - results of the national telephone health interview survey 'German Health Update (GEDA)' 2009. *BMC Public Health* 2013; **13**: 166.
12. Ziegler D, Rathmann W, Dickhaus T, Meisinger C, Mielck A, Group KS. Neuropathic pain in diabetes, prediabetes and normal glucose tolerance: the MONICA/KORA Augsburg Surveys S2 and S3. *Pain Med* 2009; **10**(2): 393-400.
13. Varas-Lorenzo C, Rueda de Castro AM, Maguire A, Miret M. Prevalence of glucose metabolism abnormalities and cardiovascular co-morbidity in the US elderly adult population. *Pharmacoepidemiol Drug Saf* 2006; **15**(5): 317-26.
14. Naess S, Eriksen J, Midthjell K, Tambs K. Diabetes mellitus and comorbidity. Change between 1984-1986 and 1995-1997: results of the Nord-Trondelag Health Study. *J Diabetes Complications* 2003; **17**(6): 323-30.
15. McGrath N, K ON, McHugh SM, Toomey E, Kearney PM. Epidemiology of undiagnosed depression in people with diabetes mellitus: a comparative analysis of Ireland, England and the USA. BMJ Open. 2021;11(10):e049155. doi:10.1136/bmjopen-2021-049155.
16. Huang T, Lin BM, Stampfer MJ, Tworoger SS, Hu FB, Redline S. A Population-Based Study of the Bidirectional Association Between Obstructive Sleep Apnea and Type 2 Diabetes in Three Prospective U.S. Cohorts. *Diabetes care* 2018; 41(10): 2111-9.
17. Carey IM, Critchley JA, DeWilde S, Harris T, Hosking FJ, Cook DG. Risk of Infection in Type 1 and Type 2 Diabetes Compared With the General Population: A Matched Cohort Study. *Diabetes care* 2018; 41(3): 513-21.
18. Ballotari P, Vicentini M, Manicardi V, et al. Diabetes and risk of cancer incidence: results from a population-based cohort study in northern Italy. *BMC Cancer* 2017; 17(1): 703.
19. Shinkov A, Borissova AM, Kovatcheva R, et al. Increased prevalence of depression and anxiety among subjects with metabolic syndrome and known type 2 diabetes mellitus - a population-based study. *Postgrad Med* 2018; **130**(2): 251-7.

20. Hamilton E, Davis WA, Bruce DG, Davis TM. Influence of Premature Mortality on the Link Between Type 2 Diabetes and Hip Fracture: The Fremantle Diabetes Study. *The Journal of clinical endocrinology and metabolism* 2017; **102**(2): 551-9.
21. Deschenes SS, Burns RJ, Graham E, Schmitz N. Prediabetes, depressive and anxiety symptoms, and risk of type 2 diabetes: A community-based cohort study. *J Psychosom Res* 2016; **89**: 85-90.
22. Fleiner HF, Bjoro T, Midthjell K, Grill V, Asvold BO. Prevalence of Thyroid Dysfunction in Autoimmune and Type 2 Diabetes: The Population-Based HUNT Study in Norway. *The Journal of clinical endocrinology and metabolism* 2016; **101**(2): 669-77.
23. Berge LI, Riise T, Tell GS, et al. Depression in persons with diabetes by age and antidiabetic treatment: a cross-sectional analysis with data from the Hordaland Health Study. *PLoS One* 2015; **10**(5): e0127161.
24. Peeters PJ, Bazelier MT, Leufkens HG, de Vries F, De Bruin ML. The risk of colorectal cancer in patients with type 2 diabetes: associations with treatment stage and obesity. *Diabetes care* 2015; **38**(3): 495-502.
25. Zhao D, Cho J, Kim MH, Friedman D, Guallar E. Diabetes, glucose metabolism, and glaucoma: the 2005-2008 National Health and Nutrition Examination Survey. *PLoS One* 2014; **9**(11): e112460.
26. Wandell P, Ljunggren G, Wahlstrom L, Carlsson AC. Diabetes and psychiatric illness in the total population of Stockholm. *J Psychosom Res* 2014; **77**(3): 169-73.
27. Icks A, Albers B, Haastert B, et al. Risk for high depressive symptoms in diagnosed and previously undetected diabetes: 5-year follow-up results of the Heinz Nixdorf Recall study. *PLoS One* 2013; **8**(2): e56300.
28. Redaniel MT, Jeffreys M, May MT, Ben-Shlomo Y, Martin RM. Associations of type 2 diabetes and diabetes treatment with breast cancer risk and mortality: a population-based cohort study among British women. *Cancer Causes Control* 2012; **23**(11): 1785-95.
29. Kengne AP, Czernichow S, Hamer M, Batty GD, Stamatakis E. Anaemia, haemoglobin level and cause-specific mortality in people with and without diabetes. *PLoS One* 2012; **7**(8): e41875.
30. Plantinga L, Rao MN, Schillinger D. Prevalence of self-reported sleep problems among people with diabetes in the United States, 2005-2008. *Prev Chronic Dis* 2012; **9**: E76.
31. Bouwman V, Adriaanse MC, van 't Riet E, Snoek FJ, Dekker JM, Nijpels G. Depression, anxiety and glucose metabolism in the general dutch population: the new Hoorn study. *PLoS One* 2010; **5**(4): e9971.
32. Gagnon C, Magliano DJ, Ebeling PR, et al. Association between hyperglycaemia and fracture risk in non-diabetic middle-aged and older Australians: a national, population-based prospective study (AusDiab). *Osteoporos Int* 2010; **21**(12): 2067-74.
33. Mitchell P, Gopinath B, McMahon CM, et al. Relationship of Type 2 diabetes to the prevalence, incidence and progression of age-related hearing loss. *Diabet Med* 2009; **26**(5): 483-8.
34. Lin EH, Von Korff M, Alonso J, et al. Mental disorders among persons with diabetes--results from the World Mental Health Surveys. *J Psychosom Res* 2008; **65**(6): 571-80.
35. Li C, Barker L, Ford ES, Zhang X, Strine TW, Mokdad AH. Diabetes and anxiety in US adults: findings from the 2006 Behavioral Risk Factor Surveillance System. *Diabet Med* 2008; **25**(7): 878-81.
36. Adriaanse MC, Dekker JM, Heine RJ, et al. Symptoms of depression in people with impaired glucose metabolism or Type 2 diabetes mellitus: The Hoorn Study. *Diabet Med* 2008; **25**(7): 843-9.
37. Kornum JB, Thomsen RW, Riis A, Lervang HH, Schonheyder HC, Sorensen HT. Diabetes, glycemic control, and risk of hospitalization with pneumonia: a population-based case-control study. *Diabetes care* 2008; **31**(8): 1541-5.
38. Hicks CW, Wang D, Windham BG, Matsushita K, Selvin E. Prevalence of peripheral neuropathy defined by monofilament insensitivity in middle-aged and older adults in two US cohorts. Sci Rep. 2021;11(1):19159. doi:10.1038/s41598-021-98565-w.
39. Boulanger M, Al-Shahi Salman R, Kerssens J, Wild SH, Scottish Diabetes Research Network Epidemiology G. Association between diabetes mellitus and incidence of intracerebral haemorrhage and case fatality rates: A retrospective population-based cohort study. *Diabetes Obes Metab* 2017; 19(8): 1193-7.

40. Wright AK, Kontopantelis E, Emsley R, et al. Life Expectancy and Cause-Specific Mortality in Type 2 Diabetes: A Population-Based Cohort Study Quantifying Relationships in Ethnic Subgroups. *Diabetes care* 2017; **40**(3): 338-45.
41. Melsom T, Schei J, Stefansson VT, et al. Prediabetes and Risk of Glomerular Hyperfiltration and Albuminuria in the General Nondiabetic Population: A Prospective Cohort Study. *Am J Kidney Dis* 2016; **67**(6): 841-50.
42. Wyld ML, Lee CM, Zhuo X, et al. Cost to government and society of chronic kidney disease stage 1-5: a national cohort study. *Intern Med J* 2015; **45**(7): 741-7.
43. Hunger M, Holle R, Meisinger C, Rathmann W, Peters A, Schunk M. Longitudinal changes in health-related quality of life in normal glucose tolerance, prediabetes and type 2 diabetes: results from the KORA S4/F4 cohort study. *Qual Life Res* 2014; **23**(9): 2515-20.
44. Hernandez D, Espejo-Gil A, Bernal-Lopez MR, et al. Association of HbA1c and cardiovascular and renal disease in an adult Mediterranean population. *BMC Nephrol* 2013; **14**: 151.
45. Bongaerts BW, Rathmann W, Kowall B, et al. Postchallenge hyperglycaemia is positively associated with diabetic polyneuropathy: the KORA F4 study. *Diabetes care* 2012; **35**(9): 1891-3.
46. Sundling V, Platou CG, Jansson RW, Bertelsen G, Wollo E, Gulbrandsen P. Retinopathy and visual impairment in diabetes, impaired glucose tolerance and normal glucose tolerance: the Nord-Trondelag Health Study (the HUNT study). *Acta Ophthalmol* 2012; **90**(3): 237-43.
47. Kucharska-Newton AM, Couper DJ, Pankow JS, et al. Diabetes and the risk of sudden cardiac death, the Atherosclerosis Risk in Communities study. *Acta Diabetol* 2010; **47 Suppl 1**: 161-8.
48. Rogers SL, Tikellis G, Cheung N, et al. Retinal arteriolar caliber predicts incident retinopathy: the Australian Diabetes, Obesity and Lifestyle (AusDiab) study. *Diabetes care* 2008; **31**(4): 761-3.

SUPPLEMENTARY METHODS

Assessments

Additional information on assessments used in the definitions for classical complications, comorbidities and cardiometabolic risk factors is described here.

Glucose metabolism status and disease duration

As described previously[1], to assess glucose metabolism status, all participants (except those who used insulin) underwent a standardized 2-hour 75-g oral glucose tolerance test after an overnight fast. For safety reasons, participants with a fasting glucose level above 11.0 mmol/L, as determined by a finger prick, did not undergo the oral glucose tolerance test. For these individuals, fasting glucose level and information about diabetes medication use were used to assess glucose metabolism status. Glucose metabolism status was defined according to the World Health Organization 2006 criteria as normal glucose metabolism (NGM), impaired fasting glucose, impaired glucose tolerance, and type 2 diabetes[1,2]. For the present study impaired fasting glucose and impaired glucose tolerance were combined into prediabetes.

Diabetes disease duration was categorized into newly diagnosed type 2 diabetes, type 2 diabetes duration 6-12 years and type 2 diabetes duration ≥ 12 years.

Questionnaires

As described previously[1], all participants underwent an extensive web-based questionnaire which included medical history, smoking behaviour, alcohol consumption, symptoms of infection, Short Form Health Survey (SF-36)[3], Rose questionnaire[4], Douleur Neuropathique 4 (DN-4)[5], Generalized Anxiety Disorder 7-item scale[6], the International Outcome Inventory for Hearing Aids[7] and a sleep questionnaire.

The medical history questionnaire included medical history of myocardial infaction; cerebral haemorrhage or infarction; coronary, carotid or leg percutaneous angioplasty and(or) coronary (bypass), carotid, or leg surgery; amputation; kidney transplantation or dialysis; (skin) malignancy which was treated by a doctor; and prior bone fracture.

As described previously[1], the smoking behaviour and alcohol consumption questionnaires included questions to define never, former and current smoking, and no, low (≤7 glasses per week for women; ≤14 glasses per week for men), or high (>7 glasses per week for women; >14 glasses per week for men) alcohol consumer[8], respectively.

A structured questionnaire on symptoms of infection was used to obtain information on recent acute infection. Participants were asked whether, in the previous two months, they had suffered from sudden symptoms such as a cough, runny nose, sore throat, fever, vomiting with fever, or pain when

urinating. They were also asked whether they had suffered from sudden onset of influenza, pneumonia, urinary tract infection, middle ear infection, diarrhea, or skin infection in the previous two months. A principal component analysis with orthogonal rotation (varimax) was conducted on the infectious symptoms to assess which symptoms could be pooled as an infectious cluster. An initial analysis was run to obtain eigenvalues for each component in the data. Four components had eigenvalues over Kaiser's criterion of 1 and the scree plot showed justification for 4 components, which is the number of components that were retained. We obtained one component each clustering the symptoms of upper respiratory infection, lower respiratory infections, gastrointestinal infections and urinary tract infections, respectively. Based on the principal component analysis, we pooled symptoms of a runny nose and sore throat as indicators of upper respiratory infection. Influenza, pneumonia and fever were pooled as indicators of lower respiratory infection. Pain when urinating and urinary tract infection were pooled as urinary tract infection. Vomiting with fever and diarrhea were pooled as gastrointestinal infection. We excluded cough from the analysis because it is strongly related to smoking and asthma, and not a specific indicator for infection. In the present study, recent acute infection was defined as self-reported symptoms of a lower respiratory, gastrointestinal or urinary tract infection in the previous two months.

A Dutch version of the SF-36 questionnaire was used to obtain limitations in mobility defined as having difficulty walking 500m or climbing the stairs.

A Dutch version of the Rose questionnaire was used to obtain information on angina pectoris, intermittent claudication and dyspnoea complaints. Presence of angina pectoris[4] was defined as having ever experienced pain or discomfort, pressure or heavy feeling, or an episode of tightness in the chest; the pain or discomfort occurs when walking the stairs, or hurrying, or walking against the wind (including individuals who do not perform these activities anymore), or when walking at an ordinary pace on the street; the individual stops or slows down or takes a sublingual nitrate when getting the pain or discomfort; the pain disappears soon (within ten minutes) after stopping or taking the sublingual nitrate; and the pain is located in the upper part of the sternum, in the lower part of the sternum, in the left front chest, or in the left arm. Presence of intermittent claudication[4,9] was defined as presence of pain in the leg(s) on walking; the pain starts when walking the stairs or hurrying (including individuals who do not perform these activities anymore) or the pain occurs when walking on the street in an ordinary pace (including individuals who do not perform these activities anymore); the pain does not start when standing still or sitting; the pain occurs in the calf or amongst others in the calf (but not somewhere else in the leg); the pain does not disappear while still walking; the individuals stops or slows down when getting the pain when walking; and the pain disappears within 10 minutes after stopping. Presence of dyspnoea complaints was defined as presence of dyspnoea with walking faster or walking the stairs; dyspnoea when walking on street with other people; or (sometimes) dyspnoea with washing or dressing yourself. Presence of

dyspnoea complaints for which they received treatment was defined as presence of dyspnoea complaints complemented with self-reported treatment for dyspnoea complaints by a doctor.

A Dutch version of the DN-4 interview[5] was used to determine presence of neuropathic pain. It consists of seven items grouped into two questions, which describe the neuropathic pain (burning, painful cold, electric shocks) and its associated abnormal sensations (tingling, pins and needles, numbness, itching). A score greater than 3 is considered indicative of neuropathic pain.

As described previously[10], a validated Dutch version of the 7-item Generalized Anxiety Disorder scale (GAD-7)[6] was used to assess anxiety symptoms. The GAD-7 is a self-administered questionnaire based on the DMS-IV criteria for a generalized anxiety disorder (GAD). It comprises seven items rated on a four-point scale, ranging from 0= “not at all” to 3 = “nearly every day”. Total scores range from 0 to 21, with higher scores indicating the presence of more (severe) anxiety symptoms. The presence of anxiety disorder was defined as a GAD score of ten or greater.

A Dutch version of The International Outcome Inventory for Hearing Aids (IOI-HA) questionnaire[7] included hearing aid use and the number of hearing aids. The self-reported use of bilateral hearing aids was included in the definition of hearing loss.

As described previously[11], an adapted version of the Berlin questionnaire was used to identify participants who are at high risk to have obstructive sleep apnoea[12,13]. Briefly, this is a 10-item questionnaire distributed in 3 categories related to snoring, tiredness and presence of comorbidities. Each category can be rated as positive or negative and a high risk of obstructive sleep apnoea is defined by two or three positive categories. Of note, three of the 10 items of the Berlin questionnaire were not available and were assigned zero points (i.e., not present). For a more detailed description please see table S1 of Lisan et al[11].

Medication use

Medication use was registered during a medication interview by trained staff. All reported medication was retrospectively classified by Anatomical Therapeutic Chemical (ATC) codes by 2 independent researchers. ATC codes were used to select medication groups. ATC codes considered for the variables were based on the “Farmacotherapeutisch Kompas 2016” (in Dutch) as provided by the Dutch healthcare institute (https://www.farmacotherapeutischkompas.nl).

Use of medication for glaucoma was defined as use of brimonidine (S01EA05), dorzolamide (S01EC03), brinzolamide (S01EC04), timolol (S01ED01), betaxolol (S01ED02), levobunolol (S01ED03), timolol combinations (S01ED51), latanoprost (S01EE01), bimatoprost (S01EE03), travoprost (S01EE04) or tafluprost (S01EE05). No individuals used acetazolamide (S01EC01), apraclonidine (S01EA03), carteolol (S01ED05) or brinzolamide combinations (S01EC54).

Use of chronic thyroid medication was defined as use of levothyroxine (H03AA01), liothyronine (H03AA02), thyroid gland preparations (H03AA05) or thiamazole (H03BB02). No individuals used carbimazole (H03BB01), propylthiouracil (H03BA02) or potassium iodide (V03AB21).

Use of supplemental medication for anaemia was defined as use of ferrous fumarate (B03AA02), ferrous sulphate (B03AA07), or folic acid (B03BB01) (but not in combination with methotrexate (L04AX03)). No individuals used ferrous gluconate (B03AA03) or ferrous chloride (B03AA05). Parental iron suppletion was not recorded. Due to its broad description pattern, parental hydroxocobalamin (B03BA03) was not taken into account for the definition of supplemental medication for anaemia (no individuals used cyanocobalamin (B03BA01)).

Use of statins was defined as use of HMG CoA reductase inhibitors, i.e. use of simvastatin (C10AA01), pravastatin (C10AA03), fluvastatin (C10AA04), atorvastatin (C10AA05), ezetimibe/simvastatin (C10BA02). No individuals used pravastin/fenofibrate (C10BA03), ezetimibe/atorvastatin (C10BA05) or other combinations with HMG CoA reductase inhibitors (C10BX).

Use of fibrates was defined as use of bezafibrate (C10AB02), gemfibrozil (C10AB04) or ciprofibrate (C10AB08). No individuals used pravastatin/fenofibrate (C10BA03).

Use of other lipid-modifying medication was defined as use of nicotinic acid (subscribed by physician or over-the-counter medication), acipimox (C10AD06), omega-3-fatty acids (C10AX06), ezetimibe (C10AX09), ezetimibe/simvastatin (C10BA02), and colestyramine (C10AC01). No individuals used alirocumab (C10AX14), evolocumab (C10AX13), lomitapide (C10AX12), colesevelam (C10AC04), or ezetimibe/atorvastatin (C10BA05).

Use of urate-lowering therapy was defined as use of allopurinol (M04AA01) or benzbromarone (M04AB03). No individuals used febuxostat (M04AA03) or rasburicase (V03AF07).

Use of antihypertensive medication was defined as use of atenolol (C07AB03), bisoprolol (C07AB07), metoprolol (C07AB02), pindolol (C07AA03), propranolol (C07AA05), carvedilol (C07AG02), nebivolol (C07AB12), chlorthalidone (C03BA04), hydrochlorothiazide (C03AA03), indapamide (C03BA11), amiloride/hydrochlorothiazide (C03EA01), epitizide/triamterene (C03EA03), hydrochlorothiazide/triamterene (C03EA01), bumetanide (C03CA02), furosemide (C03CA01), captopril (C09AA01), enalapril (C09AA02), fosinopril(C09AA09), lisinopril (C09AA03), perindopril (C09AA04), quinapril (C09AA06), ramipril (C09AA05), zofenopril (C09AA15), candesartan (C09CA06), irbesartan (C09CA04), losartan (C09CA01), olmesartan (C09CA08), telmisartan (C09CA07), valsartan (C09CA03), aliskiren (C09XA02), amlodipine (C08CA01), barnidipine (C08CA12), felodipine (C08CA02), lercanidipine (C08CA13), nifidepine (C08CA05), nitrendipine (C08CA08), diltiazem (C08DB01), verapamil (C08DA01), doxazosin (C02CA04), clonidine (C02AC01), triamterene (C03DB02), amlodipine/valsartan (C09DB01), amlodipine/valsartan/hydrochlorothiazide (C09DX01),

atenolol/chlorthalidone (C07CB03), bisoprolol/hydrochlorothiazide (C07BB07), candesartan/hydrochlorothiazide (C09DA06), captopril/hydrochlorothiazide (C09BA01), enalapril/hydrochlorothiazide (C09BA02), enalapril/lercanidipine (C09BB02), fosinopril/hydrochlorothiazide (C09BA09), irbersartan/hydrochlorothiazide (C09DA04), lisinopril/hydrochlorothiazide (C09BA03), losartan/hydrochlorothiazide (C09DA01), metoprolol/hydrochlorothiazide (C07BB02), olmesartan/amlodipine (C09DB02), olmesartan/amlodipine/hydrochlorothiazide (C09DX03), olmesartan/hydrochlorothiazide (C09DA08), perindopril/amlodipine (C09BB04), perindopril/indapamide (C09BA04), quinapril/hydrochlorothiazide (C09BA06), telmisartan/hydrochlorothiazide (C09DA07), valsartan/hydrochlorothiazide (C09DA03) or use of antihypertensiva not further specified. No individuals used acebutolol (C07AB04), eprosartan (C09CA02), isradipine (C08CA03), lacidipine (C08CA09), nicardipine (C08CA04), nimodipine (C08CA06), celiprolol (C07AB08), labetalol (C07AG01), ketanserin (C02KD01), moxonidine (C02AC05), uradipil (C02CA06), methyldopa (C02AB01), minoxidil (C02DC01), benazepril (C09AA07), aliskiren/amlodipine (C09XA53), aliskiren/hydrochlorothiazide (C09XA52), ramipril/hydrochlorothiazide (C09BA05), eprosartan/diuretics (C09DA02) or trandolapril/verapamil (C09BB10).

Medication use in ATC codes was used to obtain chronic ATC3 medication groups and to define polypharmacy as the use of five or more different chronic ATC3 medication groups (according to the Dutch College of General Practitioners multidisciplinary guideline on polypharmacy in the elderly[14]). Chronic medication was defined as all medication with an ATC code prescribed by a doctor that was indicated for chronic conditions (e.g. use of antibiotics was not included in this definition).

Laboratory measurements

As described previously[1], (fasting) glucose, glycated haemoglobin (HbA1c), total cholesterol, high-density lipoprotein (HDL), low-density lipoprotein (LDL) cholesterol, total-to-HDL cholesterol ratio, triglycerides, serum creatinine, serum cystatin C, haemoglobin, and uric acid were assessed in venous blood samples in the fasting state.

Plasma glucose was measured with a standard hexokinase reference method (Beckman Synchron LX20, Beckman Coulter Inc., Brea, USA till May 9th, 2012; Roche Cobas 6000, F. Hoffmann-La Roche Ltd, Basel, Switzerland from May 9th, 2012 onwards)[1]. HbA1c was measured with ion-exchange high performance liquid chromatography (HPLC) (Variant tm II, Bio-Rad, Hercules, California, USA). Serum total cholesterol, HDL cholesterol, triglycerides, creatinine and uric acid levels were assessed with standard (enzymatic and/or colourimetric) methods using an automatic analyzer (Beckman Synchron LX20, Beckman Coulter Inc., Brea, USA till May 9th, 2012; Roche Cobas 6000, F. Hoffmann-La Roche

Ltd, Basel, Switzerland from May 9th, 2012, onwards)[1]. LDL cholesterol was calculated according to the Friedewald formula when appropriate[1]. Serum creatinine measurement was based on the Jaffé method traceable to isotope dilution mass spectrometry (Synchron LX20, Beckman Coulter Inc., Brea, USA till May 9th, 2012; Roche Cobas 6000, F. Hoffmann-La Roche Ltd, Basel, Switzerland from May 9th, 2012, onwards). Serum cystatine C was measured with a particle enhanced immunotubidimetric assay standardised against ERM-DA471/IFCC (International Federation of Clinical Chemistry and Laboratory Medicine) reference material (Roche Cobas 8000, F. Hoffman-La Roche Ltd, Basel, Switzerland)[15]. Plasma haemoglobin was measured by an automated hematology system (Sysmex XE5000, Kobe, Japan)[1].

As described previously[15], to assess urinary albumin excretion (UAE), participants were requested to collect two 24-hour urine samples. Twenty-four hour UAE was obtained by multiplying urinary albumin concentration, measured with an immunoturbidimetric assay in a sample of the collected 24-hour urine of the participant (Beckman Synchron LX20, Beckman Coulter Inc., Brea, USA till May 9th, 2012; Roche Cobas 6000, F. Hoffmann-La Roche Ltd, Basel, Switzerland from May 9th, 2012, onwards), by the collection volume. Only urine collections with a collection time between 20 and 28 hours were considered valid. The UAE was extrapolated to 24-hour excretion if needed, and was preferably based on the average of two 24-hour urine collections.

Estimated glomerular filtration rate was calculated with the Chronic Kidney Disease Epidemiology Collaboration (CKD-EPI) equation based on both serum creatinine and serum cystatin C[16]. Presence of micro- or macroalbuminuria (a UAE of 30-300mg or of >300mg per 24 hours[17], respectively) was dichotomized. Both an eGFR below 60 ml/min/1.73m^2 and (micro)albuminuria were used in the definition of chronic kidney disease[18]. Cut-offs used were, for anaemia, plasma haemoglobin <13 g/dL (<8.1 mmol/L) in men or <12 g/dL (<7.5 mmol/L) in women[19]; for dyslipidaemia, HDL cholesterol level <40 mg/dL (<1.03 mmol/L) in men or <50 mg/dL (<1.29 mmol/L) in women[20], or LDL cholesterol level >100mg/dL (>2.5 mmol/L)[21]; and for hyperuricaemia, uric acid >6mg/dL (0.357 mmol/L)[22].

Anthropometric and office blood pressure measurements

As described previously[1], height and weight were measured without shoes and wearing light clothing using a calibrated stadiometer and scale to the nearest 0.1 cm or 0.5 kg (Seca, Hamburg, Germany). Body mass index (BMI) for the definition of obesity was calculated as weight in kilograms divided by height in meters squared. Obesity was defined as a BMI of 30 kg/m^2 or greater according to the International Classification of Diseases 11 (ICD-11)[23].

As described previously[1], office blood pressure for the definition of hypertension was determined three times on the right arm after a 10-minute rest period, using the non-invasive blood pressure monitor

Omron 705IT (Omron Healthcare Europe BV, Hoofddorp, The Netherlands). When the difference between measurement two and three is more than 10mmHg, a fourth measurement was performed. Average systolic and diastolic blood pressure were calculated using all available measurements. Hypertension was defined as office systolic pressure ≥140 mmHg, diastolic pressure ≥90 mmHg[24] and/or the use of antihypertensive medication.

Minnesota Code classification

As described previously[1], all participants underwent a 12-lead resting electrocardiogram. The 12-lead resting electrocardiogram was classified according to the Minnesota Code Classification System for Electrocardiographic findings[25]. The relative likelihood of coronary heart disease (CHD) was indicated by assigning a summary code to the Minnesota coding of each record, by use of the Whitehall Criteria[26]. Minnesota codes 1-1-1 to 1-2-8 and 7-1-1 to 7-1-2 were assigned to summary code 3 as probable CHD. Minnesota codes 1-3-1 and 1-3-2 or any of 4-1-1, 4-1-2, 4-2 or 4-3 if accompanied by 5-1, 5-2 or 5-3 were assigned to summary code 2 as possible CHD. All other Minnesota codes and normal codes were assigned to summary code 1 as unlikely CHD.

Persistent atrial fibrillation or atrial flutter was classified by The Minnesota Code Classification System for electrocardiographic findings code 8-3-1 or 8-3-2[25].

Vascular measurements

In the study population without cardiovascular disease, age-specific carotid-to-femoral pulse wave velocity (cfPWV) cut-offs[27] were used in the definition of aortic (arterial) stiffness; age- and sex-specific carotid intima-media thickness (c-IMT) cut-offs[28] were used in the definition of subclinical atherosclerosis; and the ankle brachial index cut-off of below 0.9 or above 1.30[29] was used in the definition of prior peripheral artery disease.

As described previously[30], cfPWV and c-IMT were assessed noninvasively by means of vascular ultrasound and applanation tonometry. All measurements were done by trained vascular technicians unaware of the participants' clinical or diabetes status, in a dark, quiet temperature controlled room (21-23°C). Participants were asked to refrain from smoking and drinking coffee or tea or alcoholic beverages three hours prior to the study. Participants were allowed to have a light meal (breakfast and(or) lunch). All measurements were performed in supine position after ten minutes of rest. Talking or sleeping was not allowed during the examination. During the vascular measurements (approximately 45 minutes), brachial systolic, diastolic and mean arterial pressure (MAP) were determined every five minutes with an oscillometric device (Accutorr Plus, Datascope Inc., Montvale, NJ, USA). The mean MAP and heart rate

(HR) of these measurements were used in the statistical analyses. A three-lead electrocardiogram was recorded continuously during the measurements to facilitate automatic signal processing.

Carotid to femoral pulse wave velocity (cfPWV) was determined according to recent guidelines[31] with the use of applanation tonometry (SphygmoCor, Atcor Medical, Sydney, Australia). Pressure waveforms were determined at the right common carotid and right common femoral arteries. Difference in the time of pulse arrival from the R-wave of the electrocardiogram between the two sites (transit time) was determined with the intersecting tangents algorithm. The pulse wave travel distance was calculated as 80% of the direct straight distance (measured with an infantometer) between the two arterial sites. The median of three consecutive cfPWV (defined as traveled distance/transit time) recordings was used in the analyses.

The left common carotid (10 mm proximal to the carotid bulb) was obtained with the use of an ultrasound scanner equipped with a 7.5-MHz linear probe (MyLab 70, Esaote Europe B.V., Maastricht, the Netherlands). This setup enables the measurement of diameter, distension and intima-media thickness (IMT) as described previously[32,33]. Briefly, during the ultrasound measurements a B-mode image on the basis of 19 M-lines was depicted on screen and an online echo-tracking algorithm showed real-time anterior and posterior arterial wall displacements. The M-mode recordings were composed of 19 simultaneous recordings at a frame rate of 498 Hz. The distance between the M-line recording positions was 0.96 mm, thus, a total segment of 18.24 mm of each artery was covered by the scan plane. For offline processing, the radiofrequency signal was fed into a dedicated PC-based acquisition system (ART.LAB, Esaote Europe B.V. Maastricht, the Netherlands) with a sampling frequency of 50 MHz Data processing was performed in MatLab (version 7.5, Mathworks, Natick, MA, USA). The distension waveforms were obtained from the radio frequency data with the use of a wall track algorithm [32]. Carotid IMT (c-IMT) was defined as the distance of the posterior wall from the leading edge interface between lumen and intima to the leading edge interface between media and adventitia [33]. The median c-IMT of three measurements was used in the analyses.

As described previously[1], the Omron VP2000 (Omron, Kyoto, Japan) was used to automatically determine the ankle-brachial index (ABI) based on blood pressure measurements at the brachial artery of the left and right arm, and above the left and right ankle joint. The ABI at each leg was calculated by dividing the systolic blood pressure (SBP) above the ankle joint by the higher SBP of the right or left arm[34]. An ABI <0.90 or >1.30 in either leg was considered indicative of peripheral artery disease (PAD)[29]. When the ABI was available in only one leg, and its value did not meet the criteria for PAD, the variable was coded as missing.

Peripheral vibration perception neurothesiometer score

As described previously[1], the peripheral vibration threshold was tested three times with a Horwell Neurothesiometer (NTM) at the distal phalanx of the hallux of the right and left foot (Scientific Laboratory Supplies, Nottingham, UK). The mean of the three NTM scores was calculated for each foot. However, if the coefficient of variation (CV) of the three measurements exceeded 40%, the outlying NTM score was excluded and the mean of the remaining two values was computed. For the current analyses, the NTM scores of the right leg were used.

Estimates of normal vibration perception thresholds were calculated in a healthy subpopulation of the Maastricht Study with NTM data available, and without (poly)neuropathy or diseases predisposing to (poly)neuropathy. As exclusion criteria, (poly)neuropathy was defined as either self-reported neuropathic pain (DN4-interview score > 3), and/or a severely asymmetric vibration perception (i.e. an extreme difference (values < or > 2SD) between the right and the left NTM score); and the following diseases which predispose to neuropathy were identified: prediabetes, type 2 diabetes, an ankle brachial index <0.90 in either leg, alcohol abuse (defined as >21 glasses per week for either men or women[35]), reduced kidney function (defined as either as a CKD-EPI below 45 ml/min/1.73m2, kidney transplantation or dialysis), or severe movement limitations (defined as having difficulty walking 500m or climbing the stairs based on the SF-36 questionnaire).

In statistical analyses, the NTM variable was log-transformed due to a skewed distribution. Linear regression analyses were used to determine the 97.5th percentile for the log-transformed NTM score as a function of sex and height[36]. Based on these estimations the 97.5 percentile according to sex and height was computed for each individual in the full dataset. A disturbed vibratory perception was defined as a log-transformed NTM score greater than the predicted value in either leg.

Eye measurements

The Automatic Refractor (Tonoref II) was used to measure intra-ocular pressure in both eyes to be used in the definition of ocular hypertension (an intra-ocular pressure higher than 21 mmHg in either the right or the left eye)[37].

As described previously[1], fundus photographs were performed to assess retinopathy. Once the pupils were dilated with tropicamide 0.5% and phenylephrine 2.5%, fundus photography of both eyes was performed. All fundus photographs were made with an auto fundus camera (Model AFC-230, Nidek, Gamagori, Japan) in 45 degrees of at least three fields: one field centered on the optic disc, one field centered on the macula and one temporal field positioned one disc-diameter from the center of the macula. These fundus photographs were evaluated by a trained and experienced grader (EDC) in a masked fashion and in case of any doubt or an abnormal finding, the fundus photograph was discussed with an experienced ophthalmologist (JS). Based on these fundus photographs, diabetic retinopathy was graded

according to the Diabetic Retinopathy Disease Severity Scale and the International Clinical Diabetic Retinopathy Disease Severity Scale (no diabetic retinopathy (DRP) = no abnormalities; mild non-proliferative DRP = micro-aneurysms only; moderate non-proliferative DRP = more than just micro-aneurysms but less than severe non-proliferative DRP; severe non-proliferative DRP: severe intraretinal haemorrhages and microaneurysms in each of four quadrants or definite venous beading in two or more quadrants or moderate intraretinal microvascular abnormalities in one or more quadrants and no signs of proliferative retinopathy; proliferative DRP: neovascularization and/or vitreous/preretinal haemorrhage)[38]. Fundus photography was implemented some months after the start of The Maastricht Study. In 107 participants with type 2 diabetes without fundus photographs, data could be supplemented by data from the general practitioner. The highest grade of the left or right eye was counted to dichotomize the presence of retinopathy.

Brain magnetic resonance imaging

Brain magnetic resonance imaging (MRI) was used to define ischaemic stroke on brain MRI. Brain MRI measurements were implemented from December 2013 onwards until February 2017 and were available in 2313 out of 3451 participants. Brain MRI was performed on a 3T MRI scanner (Siemens Magnetom Prisma-fit Syngo MR D13D, Erlangen, Germany) by use of a 64-element head coil for parallel imaging. The MRI protocol consisted of a 3D T1-weighted (T1) sequence (TR/TE/TI2300/2.98/900 ms, 1.00 mm isotropic voxel, 176 continuous slices, matrix size of 240x250 and reconstructed matrix size of 512x51) and a T2-weighted fluid-attenuated inversion recovery (FLAIR) (TR/TE/TI 5000/394/1800 ms, 0.98x0.98x1.26 mm acquisition voxel and 0.49x0.49x1.00 mm reconstructed voxel, 176 continuous slices, acquisition matrix size of 250x250 and reconstructed matrix size of 512x51). Contra-indications for MRI assessments were the presence of a cardiac pacemaker or implantable cardioverter-defibrillator, neurostimulator, non-detachable insulin pump, metallic vascular clips or stents in the head, cochlear implant, metal-containing intra-uterine device, metal splinters or shrapnel, dentures with magnetic clip, an inside bracket, pregnancy, epilepsy, and claustrophobia. The presence of ischaemic stroke on brain MRI was systematically assessed by a radiologist and was used in the definition of prior cerebrovascular disease.

Cognitive impairment

As described previously[1,39], all participants underwent a concise battery of neuropsychological tests to assess cognitive performance. In the present study, cognitive impairment was defined as need for additional cognitive diagnostics after the first cognitive test battery based on having an age-, sex- and educational level-adjusted score below 1.5 SD on either the immediate recall or delayed recall from the Verbal Learning

test[40], or from the STROOP III test as part of the Stroop Color-Word test[41]; having a MMSE score below 24[42]; or when two or more tests were not performed because of known cognitive impairment.

Level of education was assessed during the cognitive assessment and was classified into eight categories commonly used in the Netherlands[43]: 1) no formal education; 2) primary education; 3) lower vocational education; 4) intermediate general secondary education; 5) intermediate vocational education; 6) higher general secondary education; 7) higher vocational education; and 8) university level of education. For the calculations of the present study, education level was further classified into low (level 1 to 3), intermediate (level 4 to 6) and high (level 7 to 8)[39].

In the Verbal Learning test[40], fifteen monosyllabic words were presented in five subsequent trials on a computer screen. Each trial was followed by an immediate recall phase. When the fifth trial was completed, a fixed battery of cognitive tests that did not involve the learning of verbal material was administered for about 20 min. This was followed unexpectedly by an additional delayed recall phase. The total number of correctly recalled words immediately after the five trials was calculated to represent immediate word recall whereas the number of correctly recalled words after 20 minutes was calculated to represent delayed word recall.

In the Stroop Color-Word test, which consisted of three subtasks represented by cards, i.e. a card presenting four colour words in random order (red, blue, yellow, green) printed in black ink (Part I); a card presenting solid colour patches in one of these four basic colours (Part II); and a card presenting the four colour words printed in an ink colour incongruent to the colour word (Part III). The participants were instructed to read respectively the words, name the colours and name the ink colour of the printed words as quickly and accurately as possible[41]. The test score was the time need to complete the individual parts. In the present study, only the score of part III was used.

MINI-International Neuropsychiatric Interview

As described previously[1,10], the MINI-International Neuropsychiatric Interview (MINI)[44] was used to assess depression. The MINI is a short diagnostic structured interview, used to assess the presence of major depressive disorder in the preceding two weeks according to the DSM-IV (Diagnostic and Statistical Manual of Mental Disorders, Fourth Edition; for this study questions about lifetime depression were added). A major depressive disorder was diagnosed if participants had at least one core symptom (i.e. depressed mood or loss of interest) and at least four other symptoms of depression (i.e. significant weight change of change in appetite, insomnia or hypersomnia, psychomotor agitation or retardation, fatigue or loss of energy, guilt or worthlessness, diminished ability to think or concentrate or indecisiveness and suicidal thoughts or plans).

HearCheck Navigator

As described previously[1], the HearCheck Navigator (Siemens, Erlangen, Germany) was used to perform a screening for hearing loss in both ears. The HearCheck Navigator is a portable handheld screening audiometer which presents sounds at 35dB, 55dB and 75 dB at test frequencies of 375 Hz, 1kHz and 3kHz. All participants underwent the test in a quiet, temperature-controlled room. The device was held gently to the head of the individual, with the ear cover completely surrounding one ear ensuring skin contact all around. The participant was instructed to raise his/her hand when a tone was heard. When the hand of the participant was raised, the examiner pressed the button on the device. Whether one, three or four tones were presented was dependent of the hearing capacity of the participant. The test for the particular frequency was terminated automatically when the button was not pressed within 12 seconds. The test result was indicated with a green, yellow, or red light, which represents no hearing loss, possible mild hearing loss, and possible moderate to severe hearing loss, respectively. This procedure was repeated for the other ear. In the present study, hearing loss, as assessed by the HearCheck Navigator, was defined as possible mild or moderate to severe hearing loss based on the best ear.

ActivPAL3 physical activity monitor

As described previously[1,45], the activPAL3 physical activity monitor (PAL Technologies, Glasgow, UK) was used to measure physical activity and sedentary behaviour. The activPAL3 is a small (53 × 35 × 7 mm), lightweight (15 g) triaxial accelerometer that records movement in the vertical, anteroposterior, and mediolateral axes, and determines posture (sitting or lying, standing, and stepping) based on acceleration information. The device was attached directly to the skin on the front of the right thigh with transparent 3M Tegaderm™ tape, after the device had been waterproofed using a nitrile sleeve. Participants were asked to wear the accelerometer for eight consecutive days, without removing it at any time. To avoid inaccurately identifying non-wear time, participants were asked not to replace the device once removed. Data were uploaded using the activPAL software and processed using customised software written in MATLAB R2013b (MathWorks, Natick, MA, USA). Data from the first day were excluded from the analysis because participants performed physical function tests at the research centre after the device was attached. In addition, data from the final wear day providing ≤14 hours of data were excluded from the analysis. Participants were included if they provided at least 1 valid day (≥10 h of waking data).

The total amount of sedentary time was based on the sedentary posture (sitting or lying) and calculated as the mean time spent in a sedentary position during waking time per day. The method used to determine waking time has been described elsewhere[46]. In the present study, sedentary behaviour was defined as the mean time in minutes spent in a sedentary position during waking time per day above the age- and sex-specific 90th percentile in individuals with NGM.

The total amount of stepping was based on the stepping posture, and calculated as the mean time stepping during waking time per day. Stepping time (physical activity) was further classified into higher intensity physical activity (minutes with a step frequency >110 steps/min during waking time) and lower intensity physical activity (minutes with a step frequency ≤110 steps/min during waking time). In the present study, non-compliance with physical activity guidelines was defined as less than 2.5 hours per week (or 21.43 minutes per day) moderate to vigorous physical activity (WHO guidelines)[2], as measured by the mean time in minutes spent in higher intensity physical activity per day (minutes with a step frequency of >110 steps/min during waking time)[47].

Abdominal magnetic resonance imaging

As described previously[48], intrahepatic lipid content was assessed through Dixon-MRI using a 3.0 Tesla MRI system (MAGNETOM Prismafit, Siemens Healthineers, Erlangen, Germany) with body matrix and supine radiofrequency coils. Abdominal MRI measurements were implemented from December 2013 onwards and were available in 2229 out of 3451 participants. After a scout scan, transversal two-dimensional T2-weighted True Fast Imaging with Steady-State Free Precession (T2w TRUFI) images were acquired through the liver with the following parameters: voxel size: 1.2×1.2×5.0 mm^3, repetition time (TR): 422 ms, echo time (TE): 1.65 ms, flip angle: 60°, number of signal averages: 1, parallel imaging (GRAPPA) factor: 2. Next, transversal two-dimensional turbo spin echo Dixon-MR images were acquired through the liver during a breath hold using the following parameters: voxel size: 2.0×2.0×6.0 mm^3, number of slices: 4, TR: 500 ms, TE: 31 ms, turbo factor: 5, number of signal averages: 1, parallel imaging (GRAPPA) factor: 3. Three regions-of-interest (ROIs) were drawn in the liver by trained observers on the T2w TRUFI images. Subsequently, these ROIs were copied to the water and fat Dixon-MR images to calculate the intrahepatic lipid fraction.

This method was validated and calibrated against proton magnetic resonance spectroscopy (^{1}H-MRS), the gold standard to non-invasively quantify IHL, in 36 participants. After calibration, the intra-class correlation coefficient between Dixon-MRI and ^{1}H-MRS was 0.989 (95%CI: 0.979;0.994). IHL was expressed as the ratio CH_2/H_2O (*100%). Non-alcoholic fatty liver disease was defined as an intrahepatic lipid content ≥5.56%[49]. This cut-off value, originally expressed as (CH2/(H2O+CH2)) (1), corresponds to 5.89% (=0.0556/(1-0.0556)) when IHL is expressed as CH2/H2O, as was done in the present study.

REFERENCES

1 Schram, M. T. *et al.* The Maastricht Study: an extensive phenotyping study on determinants of type 2 diabetes, its complications and its comorbidities. *European journal of epidemiology* **29**, 439-451, doi:10.1007/s10654-014-9889-0 (2014).

2 World Health Organization. Definition and diagnosis of diabetes mellitus and intermediate hyperglycemia: report of a WHO/IDF consultation. *Geneva, Switzerland* (2006).

3 Ware, J., Snow, K., Kosinski, M. & Gandex, M. SF-36 Health Survey Manual and Interpretation Guide. *The Health Institute, New England Medical Center, Boston MA* (1993).

4 Rose, G., McCartney, P. & Reid, D. D. Self-administration of a questionnaire on chest pain and intermittent claudication. *Br J Prev Soc Med* **31**, 42-48 (1977).

5 Bouhassira, D. *et al.* Comparison of pain syndromes associated with nervous or somatic lesions and development of a new neuropathic pain diagnostic questionnaire (DN4). *Pain* **114**, 29-36, doi:10.1016/j.pain.2004.12.010 (2005).

6 Spitzer, R. L., Kroenke, K., Williams, J. B. & Lowe, B. A brief measure for assessing generalized anxiety disorder: the GAD-7. *Arch Intern Med* **166**, 1092-1097, doi:10.1001/archinte.166.10.1092 (2006).

7 Cox, R. M., Stephens, D. & Kramer, S. E. Translations of the International Outcome inventory for Hearing Aids (IOI-HA). *Int J Audiol* **41**, 3-26 (2002).

8 Health Council of the Netherlands. Guidelines for a healthy diet 2006. *The Hague: Health Council of the Netherlands* **publication no. 2006/21** (2006).

9 Leng, G. C. & Fowkes, F. G. The Edinburgh Claudication Questionnaire: an improved version of the WHO/Rose Questionnaire for use in epidemiological surveys. *J Clin Epidemiol* **45**, 1101-1109 (1992).

10 van Dooren, F. E. *et al.* Psychological and personality factors in type 2 diabetes mellitus, presenting the rationale and exploratory results from The Maastricht Study, a population-based cohort study. *BMC Psychiatry* **16**, 17, doi:10.1186/s12888-016-0722-z (2016).

11 Lisan, Q. *et al.* Sleep Apnea is Associated With Accelerated Vascular Aging: Results From 2 European Community-Based Cohort Studies. *J Am Heart Assoc* **10**, e021318, doi:10.1161/JAHA.120.021318 (2021).

12 Netzer, N. C., Stoohs, R. A., Netzer, C. M., Clark, K. & Strohl, K. P. Using the Berlin Questionnaire to identify patients at risk for the sleep apnea syndrome. *Ann Intern Med* **131**, 485-491, doi:10.7326/0003-4819-131-7-199910050-00002 (1999).

13 Tan, A. *et al.* Using the Berlin Questionnaire to Predict Obstructive Sleep Apnea in the General Population. *J Clin Sleep Med* **13**, 427-432, doi:10.5664/jcsm.6496 (2017).

14 Dutch College of General Practitioners [Nederlands Huisartsen Genootschap]. Multidisciplinary guideline Polypharmacy in Elderly [Multidisciplinaire richtlijn Polyfarmacie bij ouderen]. *Nederlands Huisartsen Genootschap* (2012).

15 Martens, R. J. *et al.* Estimated GFR, Albuminuria, and Cognitive Performance: The Maastricht Study. *Am J Kidney Dis*, doi:10.1053/j.ajkd.2016.04.017 (2016).

16 Inker, L. A. *et al.* Estimating glomerular filtration rate from serum creatinine and cystatin C. *The New England journal of medicine* **367**, 20-29, doi:10.1056/NEJMoa1114248 (2012).

17 K/DOQI clinical practice guidelines for chronic kidney disease: evaluation, classification, and stratification. *Am J Kidney Dis* **39**, S1-266 (2002).

18 National Kidney Foundation. K/DOQI clinical practice guidelines for chronic kidney disease: evaluation, classification, and stratification. *Am J Kidney Dis* **39 (2 suppl 1)**, S1-266 (2002).

19 World Health Organization. Haemoglobin concentrations for the diagnosis of anamia and assessment of severity. *Vitamin and Mineral Nutrition Information System. Geneva, World Health Organization, 2011 (WHO/NMH/NHD/MNM/11.1)*.

20 Grundy, S. M. *et al.* Diagnosis and management of the metabolic syndrome: an American Heart Association/National Heart, Lung, and Blood Institute Scientific Statement. *Circulation* **112**, 2735-2752, doi:10.1161/CIRCULATIONAHA.105.169404 (2005).

21 Executive Summary of The Third Report of The National Cholesterol Education Program (NCEP) Expert Panel on Detection, Evaluation, And Treatment of High Blood Cholesterol In Adults (Adult Treatment Panel III). *Jama* **285**, 2486-2497 (2001).

22 Bardin, T. & Richette, P. Definition of hyperuricemia and gouty conditions. *Curr Opin Rheumatol* **26**, 186-191, doi:10.1097/BOR.0000000000000028 (2014).

23 World Health Organization. ICD-11 MMS: International Classification of Diseases for Mortality and Morbidity Statistics. Eleventh Revision. *The Joint Task Force, World Health Organization*.
24 Mancia, G. *et al.* 2013 ESH/ESC guidelines for the management of arterial hypertension: the Task Force for the Management of Arterial Hypertension of the European Society of Hypertension (ESH) and of the European Society of Cardiology (ESC). *Eur Heart J* **34**, 2159-2219, doi:10.1093/eurheartj/eht151 (2013).
25 Prineas, R., Crow, R. & Zhang, Z. The Minnesota code manual of electrocardiographic findings. 2nd ed. *London: Springer-Verlag* (2010).
26 Keen, H., Morrish, N. & Lee, E. T. An analysis of serial Minnesota ECG code changes in the London cohort of the WHO Multinational Study of Vascular Disease in Diabetes. *Diabetologia* **44 Suppl 2**, S72-77 (2001).
27 Determinants of pulse wave velocity in healthy people and in the presence of cardiovascular risk factors: 'establishing normal and reference values'. *Eur Heart J* **31**, 2338-2350, doi:10.1093/eurheartj/ehq165 (2010).
28 Engelen, L., Ferreira, I., Stehouwer, C. D., Boutouyrie, P. & Laurent, S. Reference intervals for common carotid intima-media thickness measured with echotracking: relation with risk factors. *Eur Heart J* **34**, 2368-2380, doi:10.1093/eurheartj/ehs380 (2013).
29 American Diabetes Association. Peripheral arterial disease in people with diabetes. *Diabetes care* **26**, 3333-3341 (2003).
30 Veugen, M. G. *et al.* The systolic-diastolic difference in carotid stiffness is increased in type 2 diabetes: The Maastricht Study. *Journal of hypertension* **35**, 1052-1060, doi:10.1097/HJH.0000000000001298 (2017).
31 Van Bortel, L. M. *et al.* Expert consensus document on the measurement of aortic stiffness in daily practice using carotid-femoral pulse wave velocity. *Journal of hypertension* **30**, 445-448, doi:10.1097/HJH.0b013e32834fa8b0 (2012).
32 Hermeling, E., Reesink, K. D., Kornmann, L. M., Reneman, R. S. & Hoeks, A. P. The dicrotic notch as alternative time-reference point to measure local pulse wave velocity in the carotid artery by means of ultrasonography. *Journal of hypertension* **27**, 2028-2035, doi:10.1097/HJH.0b013e32832f5890 (2009).
33 Willekes, C. *et al.* Evaluation of off-line automated intima-media thickness detection of the common carotid artery based on M-line signal processing. *Ultrasound Med Biol* **25**, 57-64 (1999).
34 Aboyans, V. *et al.* Measurement and interpretation of the ankle-brachial index: a scientific statement from the American Heart Association. *Circulation* **126**, 2890-2909, doi:10.1161/CIR.0b013e318276fbcb (2012).
35 Lew, J. Q., Chow, W. H., Hollenbeck, A. R., Schatzkin, A. & Park, Y. Alcohol consumption and risk of renal cell cancer: the NIH-AARP diet and health study. *Br J Cancer* **104**, 537-541, doi:10.1038/sj.bjc.6606089 (2011).
36 O'Brien, P. C. & Dyck, P. J. Procedures for setting normal values. *Neurology* **45**, 17-23 (1995).
37 Prum, B. E., Jr. *et al.* Primary Open-Angle Glaucoma Suspect Preferred Practice Pattern((R)) Guidelines. *Ophthalmology* **123**, P112-151, doi:10.1016/j.ophtha.2015.10.055 (2016).
38 American Academy of Opthalmology Retina/Vitreous panel. Preferred Practice Pattern Guidelines. Diabetic Retinopathy. *San Francisco, CA: American Academy of Opthalmology* (2014).
39 Spauwen, P. J. *et al.* Associations of advanced glycation end-products with cognitive functions in individuals with and without type 2 diabetes: the maastricht study. *The Journal of clinical endocrinology and metabolism* **100**, 951-960, doi:10.1210/jc.2014-2754 (2015).
40 Van der Elst, W., van Boxtel, M. P., van Breukelen, G. J. & Jolles, J. Rey's verbal learning test: normative data for 1855 healthy participants aged 24-81 years and the influence of age, sex, education, and mode of presentation. *J Int Neuropsychol Soc* **11**, 290-302, doi:10.1017/S1355617705050344 (2005).
41 Van der Elst, W., Van Boxtel, M. P., Van Breukelen, G. J. & Jolles, J. The Stroop color-word test: influence of age, sex, and education; and normative data for a large sample across the adult age range. *Assessment* **13**, 62-79, doi:10.1177/1073191105283427 (2006).
42 Folstein, M. F., Folstein, S. E. & McHugh, P. R. "Mini-mental state". A practical method for grading the cognitive state of patients for the clinician. *J Psychiatr Res* **12**, 189-198 (1975).
43 De Bie, S. E. Standaardvragen 1987: Voorstellen voor Standaardvragen 1987: Voorstellen voor uniformering van vraagstellingen naar achtergrondkenmerken en interviews [Standard questions 1987: Proposal for uniformization of questions regarding background variables and interviews] *Leiden, The Netherlands: Leiden University Press* (1987).
44 Sheehan, D. V. *et al.* The Mini-International Neuropsychiatric Interview (M.I.N.I.): the development and validation of a structured diagnostic psychiatric interview for DSM-IV and ICD-10. *J Clin Psychiatry* **59 Suppl 20**, 22-33;quiz 34-57 (1998).

45 van der Berg, J. D. *et al.* Associations of total amount and patterns of sedentary behaviour with type 2 diabetes and the metabolic syndrome: The Maastricht Study. *Diabetologia* **59**, 709-718, doi:10.1007/s00125-015-3861-8 (2016).
46 van der Berg, J. D. *et al.* Identifying waking time in 24-h accelerometry data in adults using an automated algorithm. *J Sports Sci* **34**, 1867-1873, doi:10.1080/02640414.2016.1140908 (2016).
47 Tudor-Locke, C. & Rowe, D. A. Using cadence to study free-living ambulatory behaviour. *Sports Med* **42**, 381-398, doi:10.2165/11599170-000000000-00000 (2012).
48 Buziau, A. M. *et al.* Fructose Intake From Fruit Juice and Sugar-Sweetened Beverages Is Associated With Higher Intrahepatic Lipid Content: The Maastricht Study. *Diabetes care*, doi:10.2337/dc21-2123 (2022).
49 Szczepaniak, L. S. *et al.* Magnetic resonance spectroscopy to measure hepatic triglyceride content: prevalence of hepatic steatosis in the general population. *Am J Physiol Endocrinol Metab* **288**, E462-468, doi:10.1152/ajpendo.00064.2004 (2005).

SUPPLEMENTARY TABLES

Table S1. Sum scores of comorbidities, classical complications, cardiometabolic risk factors, and health burden according to glucose metabolism status – study population with complete data

	Normal glucose metabolism	Prediabetes	Type 2 diabetes	P-linear
**Comorbidities sum score (0-17)**				
Mean (SD)	1.63 (1.32)	2.22 (1.64)	3.34 (2.14)	<0.001*†‡
Mean (SE), adjusted for age and sex	1.67 (0.06)	2.17 (0.13)	3.23 (0.12)	<0.001*†‡
**Classical complications sum score (0-6)**				
Mean (SD)	0.50 (0.71)	0.62 (0.77)	1.18 (1.11)	<0.001*†‡
Mean (SE), adjusted for age and sex	0.52 (0.02)	0.57 (0.04)	1.06 (0.03)	<0.001†‡
**Cardiometabolic risk factors sum score (0-10)**				
Mean (SD)	4.08 (1.43)	5.00 (1.48)	5.52 (1.43)	<0.001*†‡
Mean (SE), adjusted for age and sex	4.21 (0.04)	4.93 (0.07)	5.31 (0.05)	<0.001*†
**Health burden sum score (0-33)**				
Mean (SD)	5.79 (2.21)	7.53 (2.49)	9.64 (3.58)	<0.001*†‡
Mean (SE), adjusted for age and sex	6.40 (0.06)	7.67 (0.11)	9.34 (0.09)	<0.001*†‡

Numbers of complete data on the individual sum scores (complete study population/normal glucose metabolism/ prediabetes/ type 2 diabetes) are 906/607/134/165 for the comorbidities score; 2,286/1,252/334/700 for the classical complications sum score; 2,539/1,414/393/733 for the cardiometabolic risk factors sum score; and 653/423/110/120 for the health burden sum score. Data are presented as mean (standard deviation) or adjusted mean (standard error). Crude linear trends and differences in sum scores among groups of glucose metabolism status were tested with an analysis of variance or an independent t-test as appropriate. Age- and sex-adjusted mean values (standard error) were calculated with an analysis of covariance.
Age- and sex-adjusted linear trends and differences in sum scores among groups of glucose metabolism status were tested with linear regression analyses. *Prediabetes versus normal glucose metabolism P<0.05; †type 2 diabetes versus normal glucose metabolism P<0.001; ‡type 2 diabetes versus prediabetes P<0.05. Other P-values >0.05.

**Table S2. Prevalences of classical complications, comorbidities and cardiometabolic risk factors in the total study population and study population with complete data**			
	**Total study population (N=3,410)**	**Study population with complete data (N=653)**	**Study population with missing values (N=2,757)**
**Descriptive Variables**			
Men, N (%)	1756 (51.5)	321 (49.2)	1435 (52.0)
Age, mean yrs (SD)	59.8 ± 8.3	58.7 ± 7.8	60.1 ± 8.3
Glucose metabolism status (normal glucose metabolism/prediabetes/type 2 diabetes), %	56.4/15.0/28.6	64.8/16.8/18.4	54.4/14.5/31.0
**Comorbidities**			
Dyspnoea, N (%)[a]			
Dyspnoea complaints	830 (25.0)	116 (17.8)	714 (26.7)
Dyspnoea complaints treated by doctor	192 (5.8)	24 (3.7)	168 (6.3)
Limitations in mobility, N (%)[b]			
Mild difficulties	613 (18.5)	76 (11.6)	537 (20.7)
Severe difficulties	105 (3.2)	7 (1.1)	98 (3.7)
Prior skin malignancy, N (%)[c]	188 (5.6)	34 (5.2)	154 (5.7)
Prior Malignancy, N (%)[d]	203 (6.1)	28 (4.3)	175 (6.5)
Any thyroid disorder, N (%)[e]	120 (3.5)	19 (2.9)	101 (3.7)
Prior bone fracture, N (%)[f]	1201 (38.3)	205 (37.5)	956 (38.6)
Recent acute infection, N (%)[g]	746 (24.8)	149 (22.8)	597 (25.3)
Polypharmacy, N (%)[h]	694 (20.4)	81 (12.4)	613 (22.3)
Hearing loss, N (%)[i]	453 (13.5)	67 (10.3)	386 (14.3)
Cognitive impairment, N (%)[j]	524 (15.9)	74 (11.3)	450 (17.1)
Current depression, N (%)[k]	121 (3.7)	14 (2.1)	107 (4.1)
Anxiety disorder, N (%)[l]	141 (4.7)	24 (3.7)	117 (4.9)
Atrial fibrillation, N (%)[m]	36 (1.1)	5 (0.8)	31 (1.2)
Ocular hypertension, N (%)[n]	145 (5.9)	35 (5.4)	110 (6.1)
Anaemia, N (%)[o]	203 (6.2)	31 (4.7)	172 (6.5)
Non-alcoholic fatty liver disease, N (%)[p]	613 (27.8)	160 (24.5)	453 (29.2)
Obstructive sleep apnoea, N (%)[q]	746 (31.1)	162 (24.8)	584 (33.4)
**Classical complications**			
Prior coronary heart disease, N (%)r	466 (14.7)	56 (8.6)	410 (16.4)
Prior cerebrovascular disease, N (%)[s]	130 (3.8)	12 (1.8)	118 (4.3)
Prior peripheral artery disease, N (%)[t]			
Ankle-brachial index <0.9 or >1.30 or intermittent claudication complaints or medical history of leg angioplasty/ surgery	678 (19.9)	138 (21.1)	540 (19.6)
Medical history of amputation	23 (0.7)	1 (0.2)	22 (0.8)
Diabetic retinopathy, N (%)[u]	40 (1.4)	5 (0.8)	35 (1.6)
Chronic kidney disease, N (%)[v]			
eGFR < 60 ml/min/1.73m2 or albuminuria	338 (10.1)	41 (6.3)	297 (11.1)
Both, or a history of kidney transplantation or haemodialysis	54 (1.6)	2 (0.3)	52 (1.9)
Diabetic sensory neuropathy, N (%)[w]			
Neuropathic pain or disturbed bilateral peripheral vibration perception	533 (18.3)	84 (12.9)	449 (19.9)
Both	64 (2.2)	3 (0.5)	61 (2.7)
**Cardiometabolic risk factors**			
Smoking[x]			
Never, N (%)	1160 (34.6)	264 (40.4)	896 (33.2)
Former, N (%)	1729 (51.6)	321 (49.2)	1408 (52.2)
Current, N (%)	460 (13.7)	68 (10.4)	392 (14.5)
Alcohol use[y]			

None, N (%)	624 (18.7)	98 (15.0)	526 (19.6)
Low, N (%)	1854 (55.5)	375 (57.4)	1479 (55.0)
High, N (%)	865 (25.9)	180 (27.6)	685 (25.5)
Obesity, N (%)[z]	768 (22.5)	90 (13.8)	678(24.6)
Hypertension, N (%)[aa]	1915 (56.3)	265 (40.6)	1650 (60.0)
Non-compliance with physical activity guidelines, N (%)[ab]	1518 (58.2)	317 (48.5)	1201 (61.5)
Sedentary behaviour, N (%)[ac]	395 (15.2)	77 (11.8)	318 (16.3)
Subclinical atherosclerosis, N (%) of CVD-[ad]	1105 (77.2)	227 (75.4)	878 (77.6)
Aortic stiffness, N (%) of CVD- [ae]	144 (10.1)	33 (10.9)	111 (9.8)
Dyslipidaemia, N (%)[af]	3147 (92.4)	593 (90.8)	2554 (92.8)
Hyperuricaemia, N (%)[ag]	1245 (36.6)	194 (29.7)	1051 (38.2)

Data are presented as mean ± standard deviation (SD) or frequencies (in N(%)) as appropriate.

Numbers for the specific variables (total study population/ study population with complete data/study population with missing values) are-[a]dyspnoea (3,324/653/2,671); [b]limitations in mobility (3,308/653/2,655); [c]prior skin malignancy (3,334/1,245/2,681); [d]prior malignancy (3,335/653/2,682); [e]any thyroid disorder (3,406/653/2,753); [f]prior bone fracture (3,132/653/2,479); [g]recent acute infection (3,014/653/2,361); [h]polypharmacy (3,406/653/2,753); [i]hearing loss (3,356/653/2,703); [j]cognitive impairment (3,291/653/2,638); [k]current depression (3,267/653/2,614); [l]anxiety disorder (3,025/653/2,372); [m]atrial fibrillation (3,329/653/2,676); [n]ocular hypertension (2,468/653/1,815); [o]anaemia (3,298/653/2,645); [p]non-alcoholic fatty liver disease (2,202/653/1,549); [q]obstructive sleep apnoea (2,399/653/1,746); [r]prior coronary heart disease (3,160/653/2,507); [s]prior cerebrovascular disease (3,380/653/2,727); [t]prior peripheral artery disease (3,404/653/2,751); [u]diabetic retinopathy (2,873/653/2,170); [v]chronic kidney disease (3,340/653/2,687); [w]diabetic sensory neuropathy (2,914/653/2,261); [x]smoking (3,349/653/2,696); [y]alcohol use (3,343/653/2,690);[z]obesity (3,407/653/2,754); [aa]hypertension (3,404/653/2,751); [ab]non-compliance with physical activity guidelines (2,607/653/1,954); [ac]sedentary behaviour (2,607/653/1,954); [ad]subclinical atherosclerosis, n of population without cardiovascular disease (n of CVD-; 1,432/301/1,131); [ae]aortic stiffness, n of population without cardiovascular disease (n of CVD-; 1,430/302/1,128); [af]dyslipidaemia (3,405/653/2,752); [ag]hyperuricaemia (3,404/653/2,751).

Table S3. Sum scores of comorbidities, classical complications, cardiometabolic risk factors, and health burden according to glucose metabolism status stratified by sex

Men	Normal glucose metabolism	Prediabetes	Type 2 diabetes	P-linear
**Comorbidities sum score (0-17)**				
Mean (SD)	1.80 (1.43)	2.48 (1.68)	3.23 (1.89)	<0.001*†‡
Mean (SE), adjusted for age	1.87 (0.06)	2.44 (0.10)	3.17 (0.06)	<0.001*†‡ §
**Classical complications sum score (0-6)**				
Mean (SD)	0.55 (0.73)	0.68 (0.78)	1.24 (1.09)	<0.001*†‡
Mean (SE), adjusted for age	0.61 (0.03)	0.64 (0.05)	1.18 (0.03)	<0.001†‡ §
**Cardiometabolic risk factors sum score (0-10)**				
Mean (SD)	4.37 (1.38)	5.23 (1.37)	5.46 (1.45)	<0.001*†‡
Mean (SE), adjusted for age	4.42 (0.05)	5.21 (0.09)	5.41 (0.06)	<0.001*†‡ §
**Health burden sum score (0-33)**				
Mean (SD)	6.72 (2.37)	8.39 (2.68)	9.93 (3.02)	<0.001*†‡
Mean (SE), adjusted for age	6.89 (0.09)	8.29 (0.16)	9.76 (0.10)	<0.001*†‡
**Women**	**Normal glucose metabolism**	**Prediabetes**	**Type 2 diabetes**	**P-linear**
**Comorbidities sum score (0-17)**				
Mean (SD)	1.73 (1.47)	2.38 (1.62)	3.54 (2.12)	<0.001*†‡
Mean (SE), adjusted for age	1.77 (0.05)	2.32 (0.11)	3.45 (0.09)	<0.001*†‡ §
**Classical complications sum score (0-6)**				
Mean (SD)	0.41 (0.66)	0.53 (0.75)	0.93 (1.02)	<0.001*†‡
Mean (SE), adjusted for age	0.43 (0.02)	0.50 (0.05)	0.88 (0.04)	<0.001†‡ §
**Cardiometabolic risk factors sum score (0-10)**				
Mean (SD)	3.65 (1.31)	4.26 (1.45)	4.71 (1.47)	<0.001*†
Mean (SE), adjusted for age	3.69 (0.04)	4.20 (0.09)	4.62 (0.08)	<0.001*† ‖
**Health burden sum score (0-33)**				
Mean (SD)	5.79 (2.34)	7.17 (2.66)	9.17 (3.33)	<0.001*†‡
Mean (SE), adjusted for age	5.89 (0.08)	7.01 (0.17)	8.94 (0.15)	<0.001*†‡

N men= 1,756; 821/275/660. N women=1,654; 1,103/236/315. Numbers (normal glucose metabolism/ prediabetes/ type 2 diabetes) for the analyses with the classical complications sum score are 1,924/511/974.

Data are presented as mean (standard deviation) or adjusted mean (standard error). Crude linear trends and differences in sum scores among groups of glucose metabolism status were tested with an analysis of variance or an independent t-test as appropriate. Age- and sex-adjusted mean values (standard error) were calculated with an analysis of covariance. Age- and sex-adjusted linear trends and differences in sum scores among groups of glucose metabolism status were tested with linear regression analyses. *Prediabetes versus normal glucose metabolism P<0.05; †type 2 diabetes versus normal glucose metabolism P<0.001; ‡type 2 diabetes versus prediabetes P<0.05. Other P-values >0.05.

Interaction between glucose metabolism status (with normal glucose metabolism as reference) and sex in the association with the comorbidities sum score, classical complications sum score, risk factors sum score, and health burden sum score was tested in the model adjusted for age. §P-interaction women as compared to men <0.10 for type 2 diabetes. ‖P-interaction women as compared to men <0.10 for prediabetes. Other P-interaction >0.10.

Table S4. Prevalence of comorbidities classical complications, and cardiometabolic risk factors according to glucose metabolism status in men

	Normal glucose metabolism (N=821)	Prediabetes (N=275)	Type 2 diabetes (N=660)	P-linear	P-linear adjusted for age
**Descriptive Variables**					
Age, mean years (SD)	58.7 (8.4)	62.1 (7.2)	63.0 (7.5)	<0.001*†	
**Comorbidities**					
Dyspnoea, N (%)[a]				<0.001*†‡	
Dyspnoea complaints	130 (16.0)	65 (24.0)	192 (30.4)		<0.001*†‡
Dyspnoea complaints treated by doctor	33 (4.0)	12 (4.4)	49 (7.8)		0.001†‡
Limitations in mobility, N (%)[b]				<0.001*†‡	
Mild difficulties	81 (10.0)	47 (17.4)	162 (25.8)		<0.001*†‡
Severe difficulties	6 (0.7)	6 (2.2)	43 (6.8)		<0.001†‡
Prior skin malignancy, N (%)[c]	46 (5.7)	23 (8.5)	32 (5.0)	0.676‡	<0.001‖
Prior malignancy, N (%)[d]	25 (3.1)	24 (8.8)	44 (6.9)	0.001*†	<0.001*
Any thyroid disorder, N (%)[e]	11 (1.3)	5 (1.8)	12 (1.8)	0.459	0.300
Prior bone fracture, N (%)[f]	322 (41.4)	108 (42.9)	219 (38.2)	0.244	0.611
Recent acute infection, N (%)[g]	160 (22.1)	49 (20.8)	151 (25.5)	0.155	0.053
Polypharmacy, N (%)[h]	53 (6.5)	36 (13.1)	322 (48.8)	<0.001*†‡	<0.001*†‡
Hearing loss, N (%)[i]	103 (12.8)	54 (19.7)	156 (24.0)	<0.001*†	0.005†
Cognitive impairment, N (%)[j]	111 (13.9)	39 (14.4)	141 (22.5)	<0.001†‡	<0.001†‡
Current depression, N (%)[k]	19 (2.4)	7 (2.6)	32 (5.2)	0.006†	0.001†
Anxiety disorder, N (%)[l]	22 (3.0)	8 (3.3)	26 (4.7)	0.112	0.012†‡
Atrial fibrillation, N (%)[m]	8 (1.0)	4 (1.5)	19 (2.9)	0.006†	0.138
Ocular hypertension, N (%)[n]	29 (5.0)	15 (8.0)	43 (9.3)	0.006†	0.032†
Anaemia, N (%)[o]	23 (2.9)	9 (3.3)	71 (11.3)	<0.001†‡	<0.001†‡
Non-alcoholic fatty liver disease, N (%)[p]	129 (21.5)	70 (36.6)	184 (52.7)	<0.001*†‡	<0.001*†‡
Obstructive sleep apnoea, N (%)[q]	168 (27.0)	100 (49.8)	237 (55.0)	<0.001*†	<0.001*†
**Classical complications**					
Prior coronary heart disease, N (%)r	84 (11.1)	40 (15.6)	178 (29.1)	<0.001†‡	<0.001†‡
Prior cerebrovascular disease, N (%)[s]	29 (3.5)	9 (3.3)	38 (5.9)	0.035†	0.347
Prior peripheral artery disease, N (%)[t]				0.016§†	
Ankle-brachial index <0.9 or >1.30 or intermittent claudication complaints or medical history of leg angioplasty/ surgery	185 (22.6)	60(21.9)	170 (25.8)		0.558
Medical history of amputation	1 (0.1)	2 (0.7)	9 (1.4)		0.009†

Diabetic retinopathy, N (%)[u]	0 (0.0)	1 (0.5)	31 (5.2)	<0.001[§†‡]	0.002[N/A]
Chronic kidney disease, N (%)[v]				<0.001[§*†‡]	
eGFR < 60 ml/min/1.73m2 or albuminuria	49 (6.1)	29 (10.7)	156 (24.1)		
Both, or a history of kidney transplantation or haemodialysis	9 (1.1)	4 (1.5)	24 (3.7)		<0.001[†‡]
Diabetic sensory neuropathy, N (%)[w]				<0.001[§†‡ǀ]	0.004[†]
Neuropathic pain or disturbed bilateral peripheral vibration perception	85 (12.0)	38 (16.7)	178 (31.9)		<0.001[†‡ǀ]
Both	8 (1.1)	3 (1.3)	35 (6.3)		<0.001[†‡]
**Cardiometabolic risk factors**					
Smoking[x]				<0.001[*†]	
Never, N (%)	311 (38.2)	68 (25.0)	154 (24.0)		
Former, N (%)	393 (48.2)	171 (62.9)	385 (60.1)		<0.001[*†]
Current, N (%)	111 (13.6)	33 (12.1)	102 (15.9)		<0.001[†]
Alcohol use[y]				<0.001[†‡]	
None, N (%)	69 (8.5)	24 (8.8)	135 (21.1)		
Low, N (%)	548 (67.3)	165 (60.7)	367 (57.3)		<0.001[†‡]
High, N (%)	197 (24.2)	83 (30.5)	138 (21.6)		<0.001[†‡]
Obesity, N (%)[z]	87 (10.6)	68 (24.7)	277 (42.1)	<0.001[*†‡]	<0.001[*†‡]
Hypertension, N (%)[aa]	419 (51.1)	189 (69.2)	563 (85.3)	<0.001[*†‡]	<0.001[*†‡]
Non-compliance with physical activity guidelines, N (%)[ab]	331 (55.5)	149 (68.0)	429 (80.2)	<0.001[*†‡]	<0.001[*†‡]
Sedentary behaviour, N (%)[ac]	58 (9.7)	41 (18.7)	130 (24.3)	<0.001[*†]	<0.001[*†]
Subclinical atherosclerosis, N (%) of CVD-[ad]	274 (73.7)	89 (84.8)	149 (73.8)	0.748[*‡]	0.394[*‡]
Aortic stiffness, N (%) of CVD- [ae]	34 (9.1)	7 (6.7)	42 (21.2)	<0.001[†‡]	<0.001[†‡]
Dyslipidaemia, N (%)[af]	753 (91.7)	265 (96.7)	634 (96.2)	<0.001[*†]	0.001[*†]
Hyperuricaemia, N (%)[ag]	385 (46.9)	179 (65.1)	385 (58.5)	<0.001[*†]	<0.001[*†]

Data are presented as mean ± SD or frequencies (in %) as appropriate. Linear trend was tested with an ANOVA or a chi-square test as appropriate. Differences among groups of glucose metabolism status were tested with an independent t-test, or chi-square test or [§]Fisher exact test, as appropriate. Age-adjusted linear trend and differences among groups of glucose metabolism status were tested with a (multinomial) logistic regression analyses (with normal glucose metabolism or prediabetes, and the lowest category as reference group).

[*]Prediabetes versus normal glucose metabolism,P<0.05; [†]type 2 diabetes versus normal glucose metabolism P<0.05; [‡]type 2 diabetes versus prediabetes P<0.05. Other p-values >0.05. [N/A]Testing differences between groups of glucose metabolism status is not applicable, due to zero number of cases in individuals with normal glucose metabolism.

Numbers for the specific variables (normal glucose metabolism/ prediabetes/ type 2 diabetes) are [a]dyspnoea (815/271/632); [b]limitations in mobility (808/270/628); [c]prior skin malignancy (811/271/636); [d]prior malignancy (814/272/637); [e]any thyroid disorder (820/274/660); [f]prior bone fracture (777/252/574); [g]recent acute infection (723/236/591); [h]polypharmacy (820/274/660); [i]hearing loss (802/274/649); [j]cognitive impairment (798/270/628); [k]current depression (789/269/620); [l]anxiety disorder (735/243/554); [m]atrial fibrillation (804/267/646); [n]ocular hypertension (584/187/464); [o]anaemia (800/270/630); [p]non-alcoholic fatty liver disease (599/191/349); [q]obstructive sleep apnoea (623/201/431); [r]prior coronary heart disease (757/257/611); [s]prior cerebrovascular disease (817/273/647); [t]prior peripheral artery disease (820/274/659); [u]diabetic retinopathy (636/220/597); [v]chronic kidney disease (809/272/647); [w]diabetic sensory neuropathy (711/227/558); [x]smoking (815/272/641); [y]alcohol use (1814/272/640);[z]obesity (820/275/658); [aa]hypertension (820/273/660); [ab]non-compliance with physical activity guidelines (596/219/535); [ac]sedentary behaviour (596/219/535); [ad]subclinical atherosclerosis, n of population without

cardiovascular disease (N of CVD-; 372/105/202); [ae]aortic stiffness, n of population without cardiovascular disease (N of CVD-; 373/104/198); [af]dyslipidaemia (821/274/659); [ag]hyperuricaemia (821/275/658).

Table S5. Prevalence of classical complications, comorbidities and cardiometabolic risk factors according to glucose metabolism status in women

	Normal glucose metabolism (N=1,103)	Prediabetes (N=236)	Type 2 diabetes (N=315)	P-linear	P-linear adjusted for age
**Descriptive Variables**					
Age, mean years (SD)	57.3 (8.0)	60.9 (7.9)	62.0 (8.1)	<0.001[$†]	
**Comorbidities**					
Dyspnoea, N (%)[a]				<0.001[*†‡]	
Dyspnoea complaints	234 (21.7)	76 (33.0)	133 (44.3)		<0.001[*†‡]
Dyspnoea complaints treated by doctor	58 (5.4)	6 (2.6)	34 (11.3)		<0.001[†‡|]
Limitations in mobility, N (%)[b]				<0.001[*†‡]	
Mild difficulties	161 (14.9)	55 (24.0)	107 (36.1)		<0.001[*†‡]
Severe difficulties	11 (1.0)	7 (3.1)	32 (10.8)		<0.001[*†‡]
Prior skin malignancy, N (%)[c]	56 (5.2)	13 (5.6)	18 (6.0)	0.567	0.531
Prior malignancy, N (%)[d]	64 (5.9)	18 (7.8)	28 (9.3)	0.030[†]	0.280
Any thyroid disorder, N (%)[e]	47 (4.3)	14 (5.9)	31 (9.9)	<0.001[†]	0.002[†]
Prior bone fracture, N (%)[f]	366 (35.1)	86 (38.7)	100 (37.9)	0.293	0.274
Recent acute infection, N (%)[g]	248 (25.2)	48 (23.0)	90 (33.2)	0.024[†‡]	0.018[†‡]
Polypharmacy, N (%)[h]	85 (7.7)	35 (14.8)	163 (51.9)	<0.001[*†‡]	<0.001[*†‡]
Hearing loss, N (%)[i]	67 (6.1)	29 (12.6)	44 (14.2)	<0.001[*†]	<0.001
Cognitive impairment, N (%)[j]	128 (12.0)	33 (14.5)	72 (24.2)	<0.001[†‡]	<0.001[†‡]
Current depression, N (%)[k]	31 (2.9)	6 (2.7)	26 (8.7)	<0.001[†‡]	<0.001[†‡]
Anxiety disorder, N (%)[l]	53 (5.3)	9 (4.2)	23 (8.5)	0.088[†]	0.072[†‡]
Atrial fibrillation, N (%)[m]	3 (0.3)	0 (0.0)	2 (0.7)	0.425	0.561
Ocular hypertension, N(%)[n]	32 (3.9)	10 (5.7)	16 (7.0)	0.038[†]	0.157
Anaemia, N (%)[o]	59 (5.5)	9 (3.9)	32 (10.6)	0.006[†‡]	<0.001[†‡]
Non-alcoholic fatty liver disease, N (%)[p]	95 (12.6)	57 (37.7)	78 (49.4)	<0.001[*†‡]	<0.001[*†‡]
Obstructive sleep apnoea, N (%)[q]	105 (12.7)	51 (32.7)	85 (52.8)	<0.001[*†‡]	<0.001[*†‡]
**Classical complications**					
Prior coronary heart disease, N (%)r	75 (7.3)	27 (12.7)	62 (21.3)	<0.001[*†‡|]	<0.001[*†‡]
Prior cerebrovascular disease, N (%)[s]	24 (2.2)	13 (5.6)	17 (5.5)	0.001[*†]	0.014[*†]
Prior peripheral artery disease, N (%)[t]				0.388[§*†]	
Ankle-brachial index <0.9 or >1.30 or intermittent claudication complaints or medical history of leg angioplasty/ surgery	180 (16.3)	24 (10.2)	59 (18.8)		0.763[*†|]
Medical history of amputation	6 (0.5)	1 (0.4)	4 (1.3)		0.400

Diabetic retinopathy, N (%)[u]	1 (0.1)	0 (0.0)	7 (2.5)	<0.001§†‡	0.006[N/A]
Chronic kidney disease, N (%)[v]				<0.001§†‡	
eGFR < 60 ml/min/1.73m2 or albuminuria	41 (3.8)	13 (5.6)	50 (16.4)		<0.001†‡
Both, or a history of kidney transplantation or haemodialysis	5 (0.5)	2 (0.9)	10 (3.3)		0.003†
Diabetic sensory neuropathy, N (%)[w]				<0.001§*†‡	
Neuropathic pain or disturbed bilateral peripheral vibration perception	115 (12.2)	45 (22.4)	72 (26.4)		<0.001[N/A]
Both	6 (0.6)	0 (0.0)	12 (4.4)		0.001[N/A]
**Cardiometabolic risk factors**					
Smoking[x]				0.129	
Never, N (%)	432 (39.7)	81 (34.9)	114 (37.9)		
Former, N (%)	522 (48.0)	120 (51.7)	138 (45.8)		0.850
Current, N (%)	134 (12.3)	31 (13.4)	49 (16.3)		0.009†
Alcohol use[y]				<0.001*†‡	
None, N (%)	191 (17.6)	55 (23.8)	150 (49.7)		
Low, N (%)	563 (51.9)	103 (44.6)	108 (35.8)		<0.001*†‡
High, N (%)	330 (30.4)	73 (31.6)	44 (14.6)		<0.001*†‡
Obesity, N (%)[z]	124 (11.2)	60 (25.4)	152 (48.3)	<0.001*†‡	<0.001*†‡
Hypertension, N (%)[aa]	362 (32.9)	134 (56.8)	248 (79.0)	<0.001*†‡	<0.001*†‡
Non-compliance with physical activity guidelines, N (%)[ab]	346 (40.9)	92 (50.8)	171 (74.0)	<0.001*†‡	<0.001†‡
Sedentary behaviour, N (%)[ac]	84 (9.9)	21 (11.6)	61 (26.4)	<0.001*†‡	<0.001†‡
Subclinical atherosclerosis, N (%) of CVD-[ad]	437 (79.3)	82 (78.1)	74 (76.3)	0.490	0.886
Aortic stiffness, N (%) of CVD- [ae]	33 (6.0)	15 (14.3)	13 (13.5)	0.001*†	<0.001*†
Dyslipidaemia, N (%)[af]	974 (88.5)	216 (91.5)	305 (96.8)	<0.001†‡	0.008†‡ǀ
Hyperuricaemia, N (%)[ag]	118 (10.7)	58 (24.6)	120 (38.2)	<0.001*†‡	<0.001*†‡

Data are presented as mean ± SD or frequencies (in %) as appropriate. Linear trend was tested with an ANOVA or a chi-square test as appropriate. Differences among groups of glucose metabolism status were tested with an independent t-test, or chi-square test or §Fisher exact test, as appropriate. Age-adjusted linear trend and differences among groups of glucose metabolism status were tested with a (multinomial) logistic regression analyses (with normal glucose metabolism or prediabetes, and the lowest category as reference group). *Prediabetes versus normal glucose metabolism,P<0.05; †type 2 diabetes versus normal glucose metabolism P<0.05; ‡type 2 diabetes versus prediabetes P<0.05. Other p-values >0.05. [N/A]Testing differences between groups of glucose metabolism status is not applicable, due to zero number of cases in individuals with normal glucose metabolism. Numbers for the specific variables (study population/normal glucose metabolism/ prediabetes/ type 2 diabetes) are [a]dyspnoea (1,706/230/300); [b]limitations in mobility (1,077/229/296); [c]prior skin malignancy (1,083/232/301); [d]prior malignancy (1,082/230/300); [e]any thyroid disorder (1,102/236/314); [f]prior bone fracture (1,043/222/264); [g]recent acute infection (984/209/271); [h]polypharmacy 1,102/236/314); [i]hearing loss (1,090/231/310); [j]cognitive impairment (1,069/228/298); [k]current depression (1,065/226/298); [l]anxiety disorder (1,008/214/271); [m]atrial fibrillation (1,080/229/303); [n]ocular hypertension (828/176/229); [o]anaemia (1,066/231/301); [p]non-alcoholic fatty liver disease (754/151/158); [q]obstructive sleep apnoea (827/156/161); [r]prior coronary heart disease (1,031/213/291); [s]prior cerebrovascular disease (1,099/234/310); [t]prior peripheral artery disease (1,102/236/313); [u]diabetic retinopathy (895/196/279); [v]chronic kidney disease (1,076/232/304); [w]diabetic sensory neuropathy (944/201/273); [x]smoking (1,088/232/301); [y]alcohol use (1,084/231/302);[z]obesity (1,103/236/315); [aa]hypertension (1,101/236/314); [ab]non-compliance with physical activity guidelines (845/181/231); [ac]sedentary behaviour

(845/181/231); [ad]subclinical atherosclerosis, n of population without cardiovascular disease (N of CVD-; 551/105/97); [ae]aortic stiffness, n of population without cardiovascular disease (N of CVD-; 554/105/96); [af]dyslipidaemia (1,100/236/315); [ag]hyperuricaemia (1,100/236/314).

Table S6. Prevalences of comorbidities classical complications, and cardiometabolic risk factors in according to glucose metabolism status with prediabetes as impaired fasting glucose and impaired glucose tolerance

	Normal glucose metabolism (N=1,924)	Impaired fasting glucose (N=143)	Impaired glucose tolerance (N=368)	Type 2 diabetes (N=975)	P-linear	P-linear adjusted for age and sex
**Descriptive Variables**						
Age, mean years (SD)	57.9 (8.2)	60.2 (7.4)	62.1 (7.6)	62.7 (7.7)	<0.001[*^†]	
**Comorbidities**						
Dyspnoea, N (%)[a]					<0.001[^†]	
Dyspnoea complaints	364 (19.2)	30 (21.3)	111 (30.8)	325 (34.9)		<0.001[^†]
Dyspnoea complaints treated by doctor	91 (4.8)	6 (4.3)	12 (3.3)	83 (8.9)		<0.001[†]
Limitations in mobility, N (%)[b]					<0.001[^†]	
Mild difficulties	242 (12.8)	16 (11.3)	86 (24.0)	269 (29.1)		<0.001[^†]
Severe difficulties	17 (0.9)	1 (0.7)	12 (3.4)	75 (8.1)		<0.001[^†]
Prior skin malignancy, N (%)[c]	102 (5.4)	9 (6.4)	27 (7.5)	50 (5.3)	0.748	0.078
Prior malignancy, N (%)[d]	89 (4.7)	14 (10.0)	28 (7.7)	72 (7.7)	0.001[*^†]	0.058[*]
Any thyroid disorder, N (%)[e]	58 (3.0)	5 (3.5)	14 (3.8)	43 (4.4)	0.052	0.002[†]
Prior bone fracture, N (%)[f]	688 (37.8)	53 (40.5)	141 (41.1)	319 (38.1)	0.660	0.692
Recent acute infection, N (%)[g]	408 (23.9)	26 (21.3)	71 (22.0)	241 (28.0)	0.056	0.004[†]
Polypharmacy, N (%)[h]	138 (7.2)	12 (8.5)	59 (16.0)	485 (49.8)	<0.001[^†]	<0.001[^†]
Hearing loss, N (%)[i]	170 (9.0)	22 (15.5)	61 (16.8)	200 (20.9)	<0.001[*^†]	0.001[†]
Cognitive impairment, N (%)[j]	239 (12.8)	16 (11.5)	56 (15.6)	213 (23.0)	<0.001[†]	<0.001[†]
Current depression, N (%)[k]	50 (2.7)	2 (1.4)	11 (3.1)	58 (6.3)	<0.001[†]	<0.001[†]
Anxiety disorder, N (%)[l]	75 (4.3)	5 (4.1)	12 (3.6)	49 (5.9)	0.125	0.005[†]
Atrial fibrillation, N (%)[m]	11 (0.6)	0 (0.0)	4 (1.1)	21 (2.2)	<0.001[†]	0.102
Ocular hypertension, N (%)[n]	61 (4.3)	8 (8.3)	17 (6.4)	59 (8.5)	<0.001[†]	0.014[†]
Anaemia, N (%)[o]	82 (4.4)	5 (3.6)	13 (3.6)	103 (11.1)	<0.001[†]	<0.001[†]
Non-alcoholic fatty liver disease, N (%)[p]	224 (16.6)	30 (30.0)	97 (40.1)	262 (51.7)	<0.001[*^†]	<0.001[*^†]
Obstructive sleep apnoea, N (%)[q]	273 (18.8)	36 (34.3)	115 (45.6)	322 (54.4)	<0.001[*^†]	<0.001[*^†]
**Classical complications**						
Prior coronary heart disease, N (%)[r]	159 (8.9)	14 (10.6)	53 (15.7)	240 (26.6)	<0.001[^†]	<0.001[^†]
Prior cerebrovascular disease, N (%)[s]	53 (2.8)	3 (2.1)	19 (5.2)	55 (5.7)	<0.001[^†]	0.016[†]
Prior peripheral artery disease, N (%)[t]					<0.001[§†]	
Ankle-brachial index <0.9 or >1.30 or intermittent claudication complaints or medical history of leg angioplasty/ surgery	365 (19.0)	18 (12.6)	66 (18.0)	229 (23.6)		0.737[*]
Medical history of amputation	7 (0.4)	1 (0.7)	2 (0.5)	13 (1.3)		0.009[†]
Diabetic retinopathy, N (%)[u]	1 (0.1)	1 (0.9)	0 (0.0)	38 (4.3)	<0.001[§†]	<0.001[†]

Chronic kidney disease, N (%)[v]					<0.001[§^†]	
eGFR < 60 ml/min/1.73m2 or albuminuria	90 (4.8)	12 (8.6)	30 (8.2)	206 (21.7)		<0.001[†]
Both, or a history of kidney transplantation or haemodialysis	14 (0.7)	1 (0.7)	5 (1.4)	34 (3.6)		<0.001[†]
Diabetic sensory neuropathy, N (%)[w]					<0.001[§^‡]	
Neuropathic pain or disturbed bilateral peripheral vibration perception	200 (12.1)	17 (14.5)	66 (21.2)	250 (8.6)		<0.001[^†]
Both	14 (0.8)	1 (0.9)	2 (0.6)	47 (5.7)		<0.001[†]
**Cardiometabolic risk factors**						
Smoking[x]					<0.001[*^†]	
Never, N (%)	743 (39.0)	36 (25.5)	113 (31.1)	268 (28.5)		
Former, N (%)	915 (48.1)	83 (58.9)	208 (57.3)	523 (55.5)		0.006[*^†]
Current, N (%)	245 (12.9)	22 (15.6)	42 (11.6)	151 (16.0)		<0.001[*†]
Alcohol use[y]					<0.001[†]	
None, N (%)	260 (13.7)	18 (12.8)	61 (16.9)	285 (30.3)		
Low, N (%)	1111 (58.5)	78 (55.3)	190 (52.5)	475 (50.4)		<0.001[^†]
High, N (%)	527 (27.8)	45 (31.9)	111 (30.7)	182 (19.3)		<0.001[†]
Obesity, N (%)[z]	211 (11.0)	35 (24.5)	93 (25.3)	429 (44.1)	<0.001[*^†]	<0.001[*^†]
Hypertension, N (%)[aa]	781 (40.7)	81 (57.0)	242 (65.9)	811 (83.3)	<0.001[*^†]	<0.001[*^†]
Non-compliance with physical activity guidelines, N (%)[ab]	677 (47.0)	59 (55.7)	182 (61.9)	600 (78.3)	<0.001[^†]	<0.001[^†]
Sedentary behaviour, N (%)[ac]	142 (9.9)	18 (17.0)	44 (15.0)	191 (24.9)	<0.001[*^†]	<0.001[*^†]
Subclinical atherosclerosis, N (%) of CVD-[ad]	711 (77.0)	57 (85.1)	114 (79.7)	223 (74.6)	0.595	0.567
Aortic stiffness, N (%) of CVD- [ae]	67 (7.2)	4 (6.1)	18 (12.6)	55 (18.7)	<0.001[^†]	<0.001[^†]
Dyslipidaemia, N (%)[af]	1727 (89.9)	137 (96.5)	344 (93.5)	939 (96.4)	<0.001[*^†]	0.001[†]
Hyperuricaemia, N (%)[ag]	503 (26.2)	76 (53.1)	161 (43.8)	505 (52.0)	<0.001[*^†]	<0.001[*^†]

Data are presented as mean ± SD or frequencies (in %) as appropriate. Linear trend was tested with an ANOVA or a chi-square test as appropriate. Differences among groups of glucose metabolism status were tested with an independent t-test, or chi-square test or [§]Fisher exact test, as appropriate. Age-adjusted linear trend and differences among groups of glucose metabolism status were tested with a (multinomial) logistic regression analyses (with normal glucose metabolism or prediabetes, and the lowest category as reference group). [*]Impaired fasting glucose versus normal glucose metabolism,P<0.05; [^]impaired glucose tolerance versus normal glucose metabolism P<0.05; [†]type 2 diabetes versus normal glucose metabolism P<0.05; . Other p-values >0.05. Numbers for the specific variables (normal glucose metabolism/ prediabetes/ type 2 diabetes) are [a]dyspnoea (1,891/141/360/932); [b]limitations in mobility (1,885/141/358/924); [c]prior skin malignancy (1,894/141/362/937); [d]prior malignancy (1,896/140/362/937); [e]any thyroid disorder (1,922/142/368/974); [f]prior bone fracture (1,820/131/343/838); [g]recent acute infection (1,708/122/323/864); [h]polypharmacy (1,922/142/368/974); [i]hearing loss (1,892/142/363/959); [j]cognitive impairment (1,867/139/359/926); [k]current depression (1,854/139/356/918); [l]anxiety disorder (1,743/121/336/825); [m]atrial fibrillation (1,884/139/3576/949); [n]ocular hypertension (1,412/96/267/693); [o]anaemia (1,864/140/361/920); [p]non-alcoholic fatty liver disease (1,353/100/242/507); [q]obstructive sleep apnea (1,450/105/252/592); [r]prior coronary heart disease (1,788/132/338/902); [s]prior cerebrovascular disease (1,916/143/364/957); [t]prior peripheral artery disease (1,922 /143/367/972); [u]diabetic retinopathy (1,531/113/303/876); [v]chronic kidney disease (1,885/140/364/951); [w]diabetic sensory neuropathy (1,655/117/311/831); [x]smoking (1,903/141/363/942); [y]alcohol use (1,898/141/362/942); [z]obesity (1,923/143/368/973); [aa]hypertension (1,921/142/367/974); [ab]non-compliance with physical activity guidelines (1,441/106/294/766); [ac]sedentary behaviour (1,441/106/294/766); [ad]subclinical atherosclerosis, n of population without cardiovascular disease (N of CVD-; 923/67/143/299); [ae]aortic stiffness, n of population without cardiovascular disease (N of CVD-; 927/66/143/294); [af]dyslipidaemia (1,921/142/368//974); [ag]hyperuricaemia (1,921/143/368/972).

Table S7. Prevalences of comorbidities classical complications, and cardiometabolic risk factors in type 2 diabetes according to diabetes duration

	Type 2 diabetes (N=975)	Newly diagnosed type 2 diabetes (N=133)	Type 2 diabetes < 6 years (N=293)	Type 2 diabetes 6-12 years (N=189)	Type 2 diabetes ≥ 12 years (N=183)	P-linear	P-linear adjusted for age and sex
**Descriptive Variables**							
Age, mean years (SD)	62.7 (7.7)	62.9 (7.6)	60.9 (8.1)	62.6 (7.3)	64.9 (6.7)	<0.001	
HbA1c, %							
**Comorbidities**							
Dyspnoea, N (%)[a]						0.297	
Dyspnoea complaints	325 (34.9)	44 (33.8)	109 (37.3)	59 (32.2)	68 (38.0)		0.392
Dyspnoea complaints treated by doctor	83 (8.9)	8 (6.2)	26 (8.9)	11 (6.0)	19 (10.6)		0.240
Limitations in mobility, N (%)[b]						0.001	
Mild difficulties	269 (29.1)	32 (24.6)	85 (29.7)	54 (29.2)	56 (31.6)		0.046
Severe difficulties	75 (8.1)	4 (3.1)	15 (5.2)	17 (9.2)	19 (10.7)		0.001
Prior skin malignancy, N (%)[c]	50 (5.3)	9 (6.8)	13 (4.5)	10 (5.4)	9 (5.0)	0.696	0.450
Prior malignancy, N (%)[d]	72 (7.7)	10 (7.7)	27 (9.2)	11 (5.9)	13 (7.2)	0.490	0.378
Any thyroid disorder, N (%)[e]	43 (4.4)	9 (6.8)	16 (5.5)	7 (3.7)	3 (1.6)	0.015	0.360
Prior bone fracture, N (%)[f]	319 (38.1)	54 (42.9)	94 (35.7)	65 (39.2)	53 (36.1)	0.463	0.643
Recent acute infection, N (%)[g]	241 (28.0)	32 (27.6)	70 (26.6)	44 (27.0)	53 (32.7)	0.281	0.135
Polypharmacy, N (%)[h]	485 (49.8)	31 (23.3)	124 (42.3)	100 (53.2)	124 (68.1)	<0.001	<0.001
Hearing loss, N (%)[i]	200 (20.9)	23 (17.7)	50 (17.4)	30 (16.0)	47 (26.1)	0.059	0.614
Cognitive impairment, N (%)[j]	213 (23.0)	21 (16.4)	62 (22.1)	46 (25.3)	41 (23.4)	0.140	0.188
Current depression, N (%)[k]	58 (6.3)	5 (3.9)	17 (6.0)	14 (7.7)	10 (5.9)	0.435	0.239
Anxiety disorder, N (%)[l]	49 (5.9)	2 (1.7)	15 (6.0)	8 (5.0)	11 (7.2)	0.104	0.046
Atrial fibrillation, N (%)[m]	21 (2.2)	1 (0.8)	5 (1.7)	5 (2.7)	3 (1.7)	0.466	0.908
Ocular hypertension, N (%)[n]	59 (8.5)	0 (0.0)	20 (9.5)	13 (10.3)	15 (12.2)	0.007	0.011
Anaemia, N (%)[o]	103 (11.1)	7 (5.5)	28 (10.0)	23 (13.1)	22 (12.9)	0.031	0.036
Non-alcoholic fatty liver disease, N (%)[p]	262 (51.7)	38 (48.1)	86 (58.1)	58 (56.9)	37 (39.4)	0.152	0.205
Obstructive sleep apnea, N (%)[q]	322 (54.4)	37 (44.0)	99 (54.1)	68 (56.2)	62 (60.2)	0.036	0.071
**Classical complications**							
Prior coronary heart disease, N (%)[r]	240 (26.6)	29 (23.4)	67 (24.5)	50 (27.9)	55 (34.6)	0.017	0.037
Prior cerebrovascular disease, N (%)[s]	55 (5.7)	4 (3.0)	21 (7.2)	12 (6.4)	12 (6.6)	0.377	0.586
Prior peripheral artery disease, N (%)[t]						0.213[§]	
Ankle-brachial index <0.9 or >1.30 or intermittent claudication complaints or	229 (23.6)	26 (19.5)	69 (23.5)	40 (21.2)	53 (29.1)		0.222

medical history of leg angioplasty/ surgery							
Medical history of amputation	13 (1.3)	3 (2.3)	2 (0.7)	2 (1.1)	4 (2.2)		0.640
Diabetic retinopathy, N (%)[u]	38 (4.3)	1 (0.9)	4 (1.5)	5 (3.0)	22 (13.2)	<0.001	<0.001
Chronic kidney disease, N (%)[v]						<0.001[§]	
eGFR < 60 ml/min/1.73m2 or albuminuria	206 (21.7)	17 (13.1)	51 (17.8)	43 (23.4)	56 (31.6)		<0.001
Both, or a history of kidney transplantation or haemodialysis	34 (3.6)	4 (3.1)	5 (1.7)	5 (2.7)	12 (6.8)		0.047
Diabetic sensory neuropathy, N (%)[w]						<0.001	
Neuropathic pain or disturbed bilateral peripheral vibration perception	250 (8.6)	23 (20.5)	75 (29.2)	40 (24.1)	61 (38.9)		0.006
Both	47 (5.7)	0 (0.0)	12 (4.7)	9 (5.4)	15 (9.6)		0.001
**Cardiometabolic risk factors**							
Smoking[x]						0.229	
Never, N (%)	268 (28.5)	43 (32.8)	79 (27.0)	58 (31.0)	47 (26.0)		
Former, N (%)	523 (55.5)	74 (56.5)	166 (56.7)	99 (52.9)	104 (57.5)		0.783
Current, N (%)	151 (16.0)	14 (10.7)	48 (16.4)	30 (16.0)	30 (16.6)		0.234
Alcohol use[y]						0.002	
None, N (%)	285 (30.3)	30 (22.9)	80 (27.4)	65 (34.6)	56 (30.8)		
Low, N (%)	475 (50.4)	56 (42.7)	161 (55.1)	91 (48.4)	96 (52.7)		0.046
High, N (%)	182 (19.3)	45 (34.4)	51 (17.5)	32 (17.0)	30 (16.5)		<0.001
Obesity, N (%)[z]	429 (44.1)	45 (33.8)	130 (44.4)	90 (47.9)	84 (46.2)	0.040	0.016
Hypertension, N (%)[aa]	811 (83.3)	103 (77.4)	226 (77.1)	168 (89.4)	168 (92.3)	<0.001	<0.001
Non-compliance with physical activity guidelines, N (%)[ab]	600 (78.3)	63 (65.6)	175 (75.8)	107 (77.0)	119 (83.2)	0.003	0.014
Sedentary behaviour, N (%)[ac]	191 (24.9)	14 (14.6)	56 (24.2)	39 (28.1)	50 (35.0)	<0.001	<0.001
Subclinical atherosclerosis, N (%) of CVD-[ad]	223 (74.6)	38 (80.9)	65 (76.5)	50 (79.4)	28 (66.7)	0.200	0.199
Aortic stiffness, N (%) of CVD- [ae]	55 (18.7)	5 (10.9)	13 (15.5)	16 (25.8)	9 (20.9)	0.082	0.082
Dyslipidaemia, N (%)[af]	939 (96.4)	124 (93.9)	284 (96.9)	182 (96.3)	176 (96.7)	0.372	0.383
Hyperuricaemia, N (%)[ag]	505 (52.0)	69 (52.3)	165 (56.3)	91 (48.4)	89 (49.2)	0.211	0.032

Diabetes duration was available in 797 out of 975 individuals with type 2 diabetes. Data are presented as mean ± SD or frequencies (in %) as appropriate. Linear trend was tested with an ANOVA or a chi-square test as appropriate or [§]Fisher exact test as appropriate. Age-adjusted linear trend and differences among groups of type 2 diabetes duration were tested with a (multinomial) logistic regression analyses (with newly diagnosed type 2 diabetes and the lowest category as reference group).

Numbers for the specific variables (type 2 diabetes/newly diagnosed type 2 diabetes/diabetes duration < 6 years/ diabetes duration 6-12 years/diabetes duration >= 12 years) are [a]dyspnoea (932/130/292/183/179); [b]limitations in mobility (924/130/286/185/177); [c]prior skin malignancy (937/132/291/185/180); [d]prior malignancy (937/130/292/185/181); [e]any thyroid disorder (974/133/293/188/182); [f]prior bone fracture (838/126/263/166/147); [g]recent acute infection (864/116/263/163/162); [h]polypharmacy (974/133/293/188/182); [i]hearing loss (959/130/288/188/180); [j]cognitive impairment (926/128/281/182/175); [k]current depression (918/127/281/181/169); [l]anxiety disorder (825/119/248/159/152); [m]atrial

fibrillation (949/130/288/184/174); [n]ocular hypertension (693/87/211/126/123); [o]anaemia (920/128/279/175/171); [p]non-alcoholic fatty liver disease (507/79/148/102/94); [q]obstructive sleep apnea (592/84/183/121/103); [r]prior coronary heart disease (902/124/274/179/159); [s]prior cerebrovascular disease (957/132/293/188/182); [t]prior peripheral artery disease (972/133/293/189/182); [u]diabetic retinopathy (876/112/269/168/167); [v]chronic kidney disease (951/130/287/184/177); [w]diabetic sensory neuropathy (831/112/257/166/157); [x]smoking (942/131/293/187/181); [y]alcohol use (942/131/292/188/182); [z]obesity (973/133/293/188/182); [aa]hypertension (974/133/293/188/182); [ab]non-compliance with physical activity guidelines (766/96/231/139/143); [ac]sedentary behaviour (766/96/231/139/143); [ad]subclinical atherosclerosis, n of population without cardiovascular disease (N of CVD-; 299/47/85/63/42); [ae]aortic stiffness, n of population without cardiovascular disease (N of CVD-; 294/46/84/62/43); [af]dyslipidaemia (974/132/293/189/182); [ag]hyperuricaemia (972/132/293/188/181

Table S8. Sum scores of comorbidities, classical complications, cardiometabolic risk factors, and health burden according to glucose metabolism status with use of age-independent cut-offs in the definition of health burden variables

	Normal glucose metabolism	Prediabetes	Type 2 diabetes	P-linear
**Cardiometabolic risk factors sum score (0-10)**				
Mean (SD)	3.77 (1.45)	4.65 (1.54)	5.17 (1.54)	<0.001*†‡
Mean (SE), adjusted for age and sex	3.92 (0.03)	4.43 (0.06)	4.55 (0.04)	<0.001*†‡
**Health burden sum score (0-33)**				
Mean (SD)	5.99 (2.52)	7.69 (2.84)	9.65 (3.21)	<0.001*†‡
Mean (SE), adjusted for age and sex	6.25 (0.06)	7.51 (0.12)	9.24 (0.09)	<0.001*†‡

N=3,410; 1,924/511/975. Data are presented as mean (standard deviation) or adjusted mean (standard error). Crude linear trends and differences in sum scores among groups of glucose metabolism status were tested with an analysis of variance or an independent t-test as appropriate. Age-and sex-adjusted mean values (standard error) were calculated with an analysis of covariance. Age- and sex-adjusted linear trends and differences in sum scores among groups of glucose metabolism status were tested with linear regression analyses. *Prediabetes versus normal glucose metabolism P<0.005; †type 2 diabetes versus normal glucose metabolism P<0.001; ‡type 2 diabetes versus prediabetes P<0.05. Other p-values >0.05.
The health burden variables sedentary behaviour, subclinical atherosclerosis and aortic stiffness were replaced by age-independent definitions of sedentary behaviour, subclinical atherosclerosis and aortic stiffness. In these analyses, we did not replace the (age-, sex- and educational level-adjusted) scores used in the definition of cognitive impairment, because the cut-offs for these scores were determined in a healthy reference population[40,41] and have been generally accepted for use in the definition of cognitive impairment.
The comorbidities and classical complications sum score were not presented here because these scores did not contain an age-specified variable.

Table S9. Associations of type 2 diabetes and prediabetes with sum scores of health burden and cardiometabolic risk factors, as compared to normal glucose metabolism

	Prediabetes versus normal glucose metabolism			Type 2 diabetes versus normal glucose metabolism			
**Use of described definitions of health burden variables**							
	B	95% CI	P	B	95% CI	P	P-trend
**Cardiometabolic risk factors sum score**							
Crude	0.82	(0.68;0.96)	<0.001	1.26	(1.15;1.37)	<0.001	<0.001
Adjusted for sex, age	0.65	(0.52;0.79)	<0.001	0.96	(0.85;1.07)	<0.001	<0.001
**Health burden sum score***							
Crude	1.64	(1.38;1.90)	<0.001	3.50	(3.30;3.71)	<0.001	<0.001
Adjusted for sex, age	1.27	(1.02;1.53)	<0.001	2.94	(2.73;3.14)	<0.001	<0.001
**Use of age-independent cut-offs in the definition of health burden variables**							
	**Prediabetes versus normal glucose metabolism**			**Type 2 diabetes versus normal glucose metabolism**			
	B	95% CI	P	B	95% CI	P	P-trend
**Cardiometabolic risk factors sum score**							
Crude	0.88	(0.74;1.03)	<0.001	1.41	(1.29;1.52)	<0.001	<0.001
Adjusted for sex, age	0.65	(0.51;0.78)	<0.001	1.01	(0.90;1.12)	<0.001	<0.001
**Health burden sum score†**							
Crude	1.70	(1.43;1.97)	<0.001	3.65	(3.44;3.89)	<0.001	<0.001
Adjusted for sex, age	1.26	(1.00;1.52)	<0.001	2.99	(2.77;3.20)	<0.001	<0.001

N= 3,410; 1,924/511/975. Data are presented as unstandardized regression coefficients (B) with 95% confidence interval (CI), which represent the difference in sum scores between individuals with prediabetes or type 2 diabetes, as compared to normal glucose metabolism, respectively. P for trend was tested with linear regression analyses.

The health burden variables sedentary behaviour, subclinical atherosclerosis and aortic stiffness were replaced by age-independent definitions of sedentary behaviour, subclinical atherosclerosis and aortic stiffness. In these analyses, we did not replace the (age-, sex- and educational level-adjusted) scores used in the definition of cognitive impairment, because the cut-offs for these scores were determined in a healthy reference population[40,41] and have been generally accepted for use in the definition of cognitive impairment. The comorbidities and classical complications sum score were not presented here because these scores did not contain an age-specified variable.

*The regression coefficient (B (95% confidence interval)) for age in the association between type 2 diabetes and prediabetes, as compared to normal glucose metabolism, and health burden was 0.07 (0.06;0.09). †The regression coefficient (B (95% confidence interval)) for age in the association between type 2 diabetes and prediabetes, as compared to normal glucose metabolism, and health burden was 0.09 (0.08;0.10).

**Table S10. Additional adjustments of sum scores of cardiometabolic risk factors, and health burden according to glucose metabolism status**				
	**Normal glucose metabolism**	**Prediabetes**	**Type 2 diabetes**	**P-linear**
**Cardiometabolic risk factors sum score (0-10)**				
Mean (SD)	3.96 (1.39)	4.78 (1.49)	5.21 (1.50)	<0.001[*†‡|]
Mean (SE), adjusted for age and sex	4.07 (0.03)	4.72 (0.06)	5.03 (0.05)	<0.001[*†‡|]
Mean (SE), adjusted for age, sex, mean arterial pressure, heart rate[§]	4.18 (0.03)	4.78 (0.07)	5.06 (0.05)	<0.001[*†‡|]
**Health burden sum score (0-33)**				
Mean (SD)	6.19 (2.40)	7.82 (2.74)	9.69 (3.14)	<0.001[*†‡|]
Mean (SE), adjusted for age and sex	6.40 (0.06)	7.67 (0.11)	9.34 (0.08)	<0.001[*†‡]
Mean (SE), adjusted for age, sex, mean arterial pressure, heart rate[§]	6.52 (0.06)	7.74 (0.12)	9.37 (0.09)	<0.001[*†‡]

N= 3,410; 1,924/511/975. [§]Vascular ultrasound data was available in N=2,959; 1,664/435/860. Data are presented as mean (standard deviation) or adjusted mean (standard error). Crude linear trends and differences in sum scores among groups of glucose metabolism status were tested with an analysis of variance or an independent t-test as appropriate. Age-and sex-adjusted mean values (standard error) were calculated with an analysis of covariance. Age- and sex-adjusted linear trends and differences in sum scores among groups of glucose metabolism status were tested with linear regression analyses. *Prediabetes versus normal glucose metabolism P<0.005; †type 2 diabetes versus normal glucose metabolism P<0.001; [‡]type 2 diabetes versus prediabetes P<0.05. Other p-values >0.05.

**Table S11. HbA1c and oral glucose tolerance test values according to glucose metabolism status (including in individuals with impaired fasting glucose and impaired glucose tolerance) to describe diabetes control**					
	**Normal glucose metabolism (N=1,924)**	**Prediabetes (N=511)**	**Impaired fasting glucose (N=143)**	**Impaired glucose tolerance (N=368)**	**Type 2 diabetes (N=975)**
HbA1c, in %[a]	5.5 (0.3)	5.7 (0.4)	5.8 (0.4)	5.7 (0.4)	6.9 (1.1)
OGTT baseline, t=0min, mmol/L[b]	5.2 (0.4)	5.9 (0.6)	6.3 (0.2)	5.7 (0.6)	7.9 (2.0)
OGTT t=15min, mmol/L[c]	6.7 (0.9)	7.6 (0.9)	7.8 (0.8)	7.5 (1.0)	9.5 (1.6)
OGTT t=30min, mmol/L[d]	7.8 (1.4)	9.4 (1.4)	9.5 (1.4)	9.4 (1.4)	12.0 (2.1)
OGTT t=45min, mmol/L[e]	7.8 (1.9)	10.4 (1.9)	10.0 (2.0)	10.5 (1.8)	14.1 (2.6)
OGTT t=60min, mmol/L[f]	7.2 (2.0)	10.5 (2.2)	9.3 (2.2)	10.9 (2.0)	15.3 (2.9)
OGTT t=90min, mmol/L[g]	6.1 (1.6)	9.4 (2.2)	7.3 (1.8)	10.2 (1.7)	15.8 (3.6)
OGTT t=120min, mmol/L[h]	5.4 (1.1)	8.1 (1.7)	5.8 (1.1)	9.0 (0.9)	14.3 (3.9)

Data are presented as mean (SD). Numbers for variables according to glucose metabolism status (normal glucose metabolism/prediabetes(impaired fasting glucose/impaired glucose tolerance)/type 2 diabetes) are [a]1917/508 (143/365)/972; [b]1922/511(143/368)/973; [c]1820/490(141/349)/687; [d]1809/484(140/344)/687; [e]1807/479(139/340)/678; [f]1791/471(136/335)/679; [g]1770/471(137/334)/676; [h]1918/508(142/366)/724.

SUPPLEMENTARY FIGURES

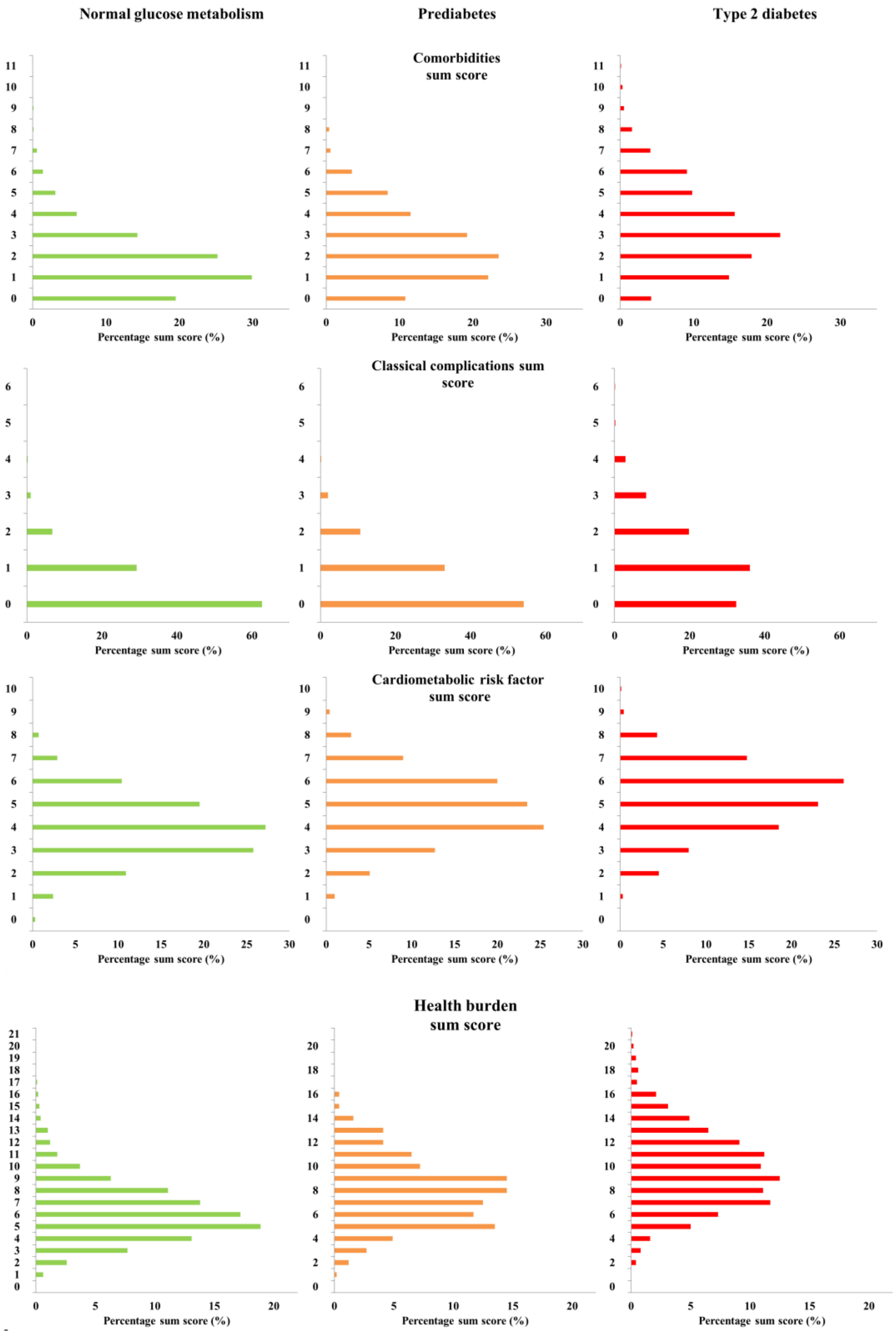

Figure S1. Distribution of the sum scores of comorbidities, classical complications, cardiometabolic risk factors, and health burden according to glucose metabolism status. The health burden sum score (0-33) is the total of the number of comorbidities (0-17), classical complications (0-6), cardiometabolic risk factors (0-10). Normal glucose metabolism (N=1,924), prediabetes (N=511), type 2 diabetes (N=975).

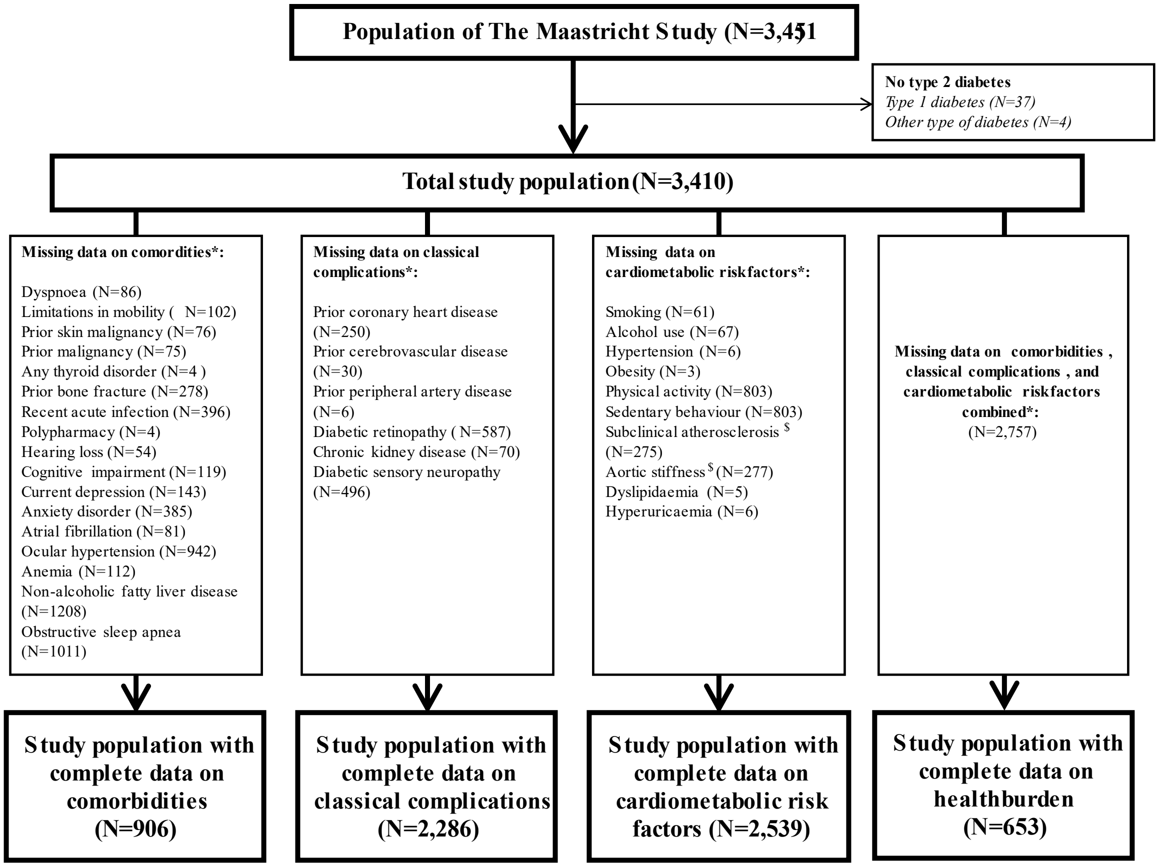

Figure S2. Overview of study populations and missing values. *Categories of missing data were not mutually exclusive. †Number of missings in individuals without prior cardiovascular disease. Prior cardiovascular disease was defined as prior coronary heart disease, prior cerebrovascular disease, prior peripheral artery disease, and(or) presence of dyspnoea.

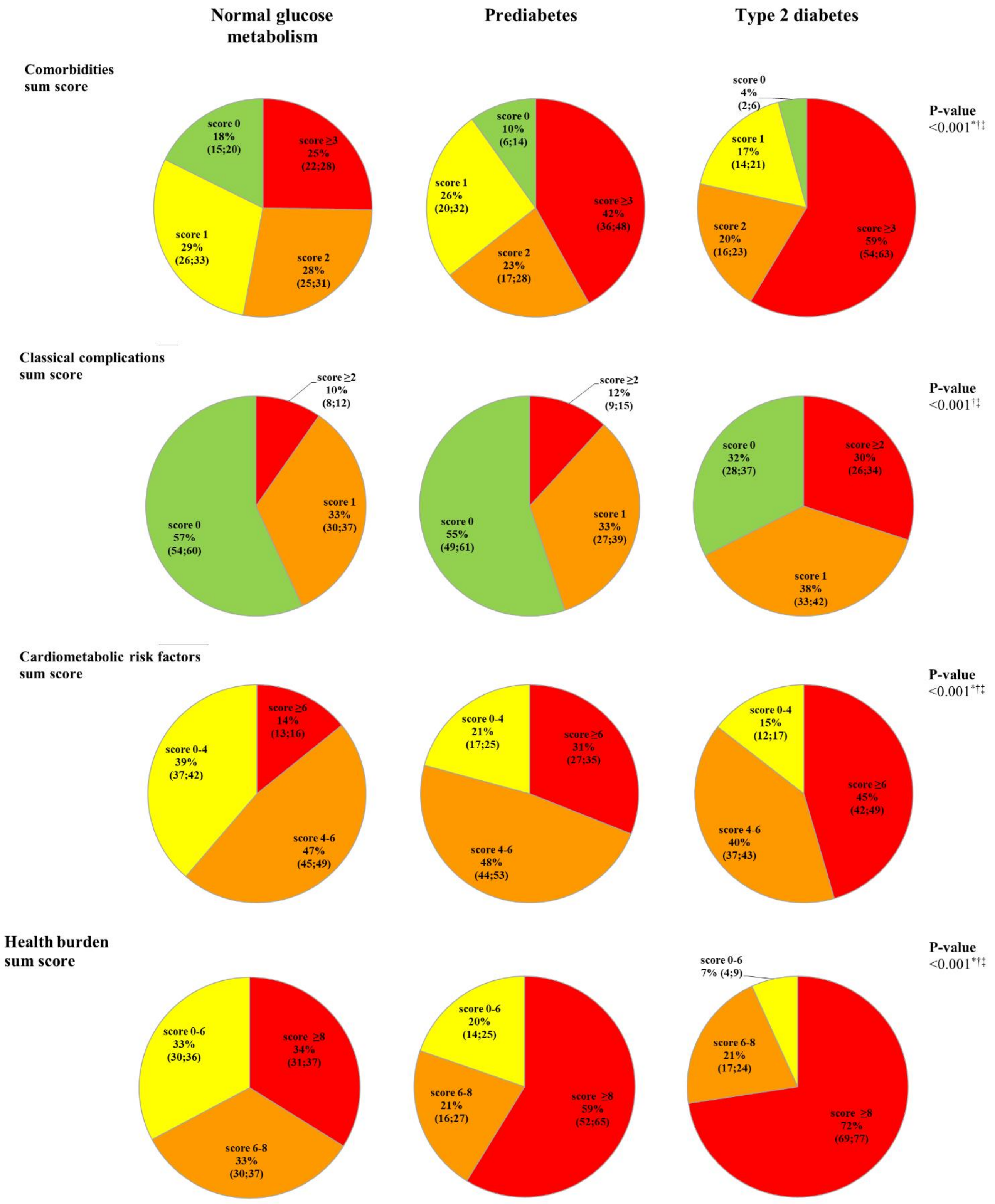

Figure S3. Age-adjusted sum scores of comorbidities, classical complications, cardiometabolic risk factors, and health burden according to glucose metabolism status in men. The health burden sum score (0-33) is the total of the number of comorbidities (0-17), classical complications (0-6), and cardiometabolic risk factors (0-10). The numeric categories of the

comorbidities, classical complications, cardiometabolic risk factors and health burden sum scores are presented as age- and sex-adjusted frequencies (percentages with their 95% confidence intervals ; with normal glucose metabolism as reference category). Normal glucose metabolism (N=821), prediabetes (N=275), type 2 diabetes (N=660). Age- and sex-adjusted linear trend and differences in categories of sum scores among groups of glucose metabolism status were tested with multinomial logistic regression analyses with normal glucose metabolism or prediabetes and the lowest sum score category as reference group. P-linear represents the trend with deteriorating glucose metabolism status for the highest sum score category, and [*, †, ‡] represent the differences between groups of glucose metabolism status per highest sum score category. [*]Prediabetes versus normal glucose metabolism P<0.001; [†]type 2 diabetes versus normal glucose metabolism P<0.005; [‡]type 2 diabetes versus prediabetes P<0.001. Other P-values >0.05. One individual with normal glucose metabolism had a cardiometabolic risk factors sum score of zero. No individual had a health burden sum score of zero.

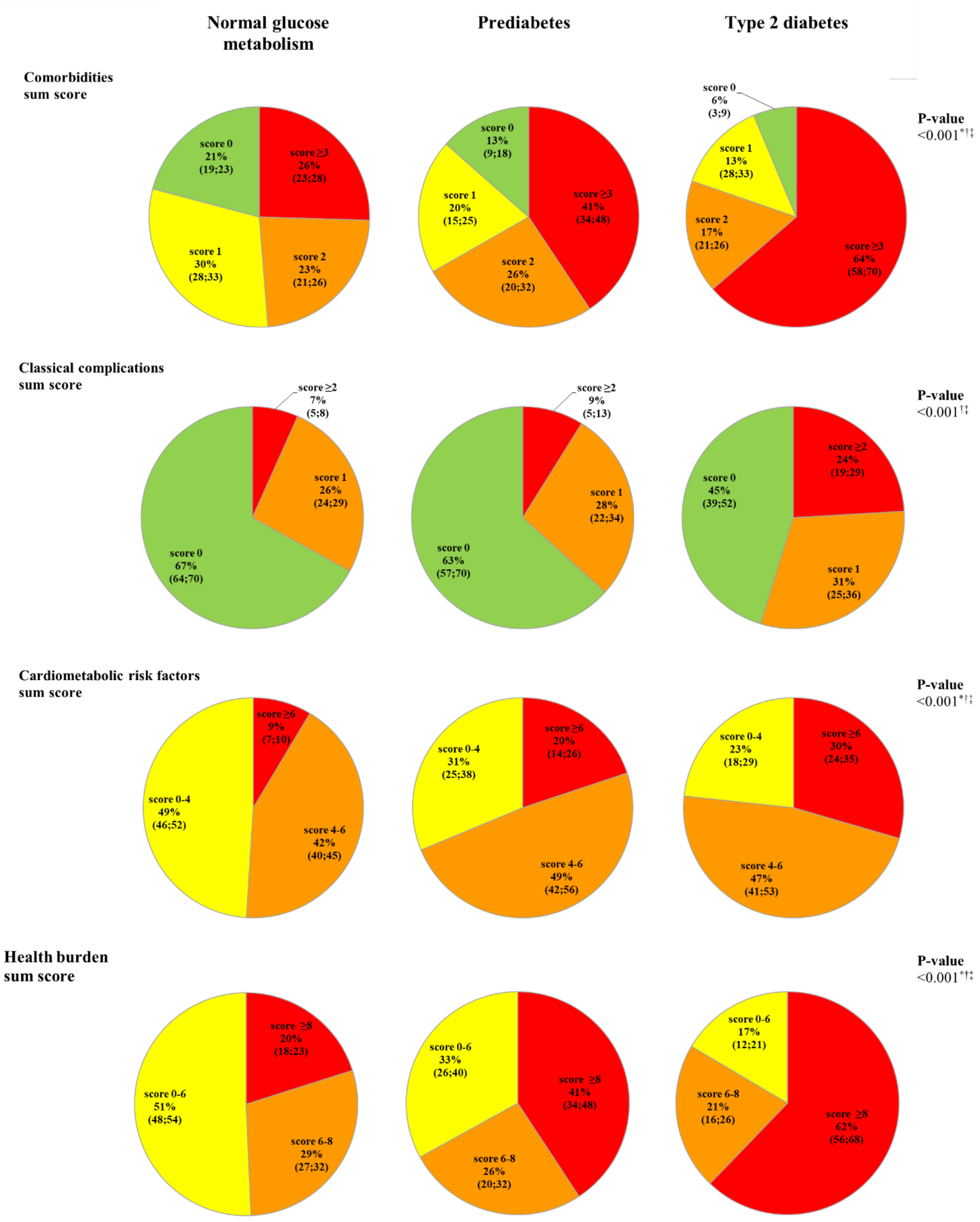

Figure S4. Age-adjusted sum scores of comorbidities, classical complications, cardiometabolic risk factors, and health burden according to glucose metabolism status in women. The health burden sum score (0-33) is the total of the number of comorbidities (0-17), classical complications (0-6), and cardiometabolic risk factors (0-10). The numeric categories of the

comorbidities, classical complications, cardiometabolic risk factors and health burden sum scores are presented as age- and sex-adjusted frequencies (percentages with their 95% confidence intervals ; with normal glucose metabolism as reference category). Normal glucose metabolism (N=1,103), prediabetes (N=236), type 2 diabetes (N=315). Age- and sex-adjusted linear trend and differences in categories of sum scores among groups of glucose metabolism status were tested with multinomial logistic regression analyses with normal glucose metabolism or prediabetes and the lowest sum score category as reference group. P-linear represents the trend with deteriorating glucose metabolism status for the highest sum score category, and [*, †, ‡] represent the differences between groups of glucose metabolism status per highest sum score category. [$]Prediabetes versus normal glucose metabolism $P<0.05$; [†]type 2 diabetes versus normal glucose metabolism $P<0.005$; [‡]type 2 diabetes versus prediabetes $P<0.005$. Other P-values >0.05. Four individuals with normal glucose metabolism had a cardiometabolic risk factors sum score of zero. No individual had a health burden sum score of zero.
